# Crystal structures of fourteen halochalcogenylphos­pho­nium tetra­halogenidoaurates(III)

**DOI:** 10.1107/S2056989024002780

**Published:** 2024-04-23

**Authors:** Daniel Upmann, Dirk Bockfeld, Peter G. Jones, Eliza Târcoveanu

**Affiliations:** a Institut für Anorganische und Analytische Chemie, Technische Universität Braunschweig, Hagenring 30, D-38106 Braunschweig, Germany; Universität Greifswald, Germany

**Keywords:** crystal structure, gold, halochalcogenyl­phospho­nium, secondary inter­actions

## Abstract

The structures of fourteen halochalcogenyl­phospho­nium tetra­halogen­ido­aurates(III), phosphane chalcogenide derivatives with general formula [*R* ^1^_3–_ *_n_R* ^2^_*n*_P*EX*][Au*X*_4_] (*R* ^1^ = *t*-butyl; *R* ^2^ = isopropyl; *n* = 0 to 3; *E* = S or Se; *X* = Cl or Br) are presented. The eight possible chlorido derivatives are: **17** ***a***, *n* = 3, *E* = S; **18** ***a***, *n* = 2, *E* = S; **19** ***a***, *n* = 1, *E* = S; **20** ***a***, *n* = 0, *E* = S; **21** ***a***, *n* = 3, *E* = Se; **22** ***a***, *n* = 2, *E* = Se; **23** ***a***, *n* = 1, *E* = Se; and **24** ***a***, *n* = 0, *E* = Se, and the corresponding bromido derivatives are **17** ***b***–**24** ***b*** in the same order. Structures were obtained for all compounds except for the tri-*t*-butyl derivatives **24** ***a*** and **24** ***b***. Isotypy is observed for **18** ***a***/**18** ***b***/**22** ***a***/**22** ***b***, **19** ***a***/**23** ***a***, **17** ***b***/**21** ***b*** and **19** ***b***/**23** ***b***. In eleven of the compounds, *X*⋯*X* contacts (mostly very short) are observed between the cation and anion, whereby the *E*—*X*⋯*X* groups are approximately linear and the *X*⋯*X*—Au angles approximately 90°. The exceptions are **17** ***a***, **19** ***a*** and **23** ***a***, which instead display short *E*⋯*X* contacts. Bond lengths in the cations correspond to single bonds P—*E* and *E*—*X*. For each group with constant *E* and *X*, the P—*E*—*X* bond-angle values increase monotonically with the steric bulk of the alkyl groups. The packing is analysed in terms of *E*⋯*X*, *X*⋯*X* (some between anions alone), H⋯*X* and H⋯Au contacts. Even for isotypic compounds, some significant differences can be discerned.

## Abstract 2

The structures of fourteen halochalcogenyl­phospho­nium tetra­halogenidoaurates(III) of general formula [*R* ^1^_3-_ *_n_R* ^2^_*n*_P*EX*][Au*X*_4_] (*R* ^1^ = *t*-butyl; *R* ^2^ = *i*-propyl; *n* = 0 to 3; *E* = S or Se; *X* = Cl or Br) are presented and compared.

## 1. Chemical context

Some years ago, in Part 3 of this series (Taouss *et al.*, 2015 ▸), we investigated the oxidation of the phosphane chalcogenide gold(I) complexes Ph_3_P*E*Au*X* (*E* = S or Se, *X* = Cl or Br) with iodo­benzene dichloride PhICl_2_ (as a more controllable substitute for elemental chlorine) or elemental bromine. We were expecting to obtain the corresponding Ph_3_P*E*Au*X*_3_ complexes, and these were indeed formed, but excess halogen led to the unexpected doubly oxidized ionic products [Ph_3_P*EX*][Au*X*_4_] (for *E* = S, *X* = Cl and Br and *E* = Se, *X* = Br), involving halochalcogenyl­phospho­nium cations, previously unknown for *X* = Cl or Br (but known for *X* = I; du Mont *et al.* (2008 ▸) and references therein). The combination *E* = Se, *X* = Cl led instead to [Ph_3_PSeCl]_2_[Au_4_Se_2_Cl_10_]. We also obtained one structure of a similar compound with [2.2]para­cyclo­phanyldiiso­propyl­phosphane and *E* = Se, *X* = Cl (Upmann *et al.*, 2019 ▸).

We then extended our studies to phosphane chalcogenides involving alkyl groups. In Part 6 of this series (Upmann *et al.*, 2024*a* ▸) we presented the structures of sixteen halogenido-gold(I) complexes of various tri­alkyl­phosphane chalcogenides, and in Part 7 (Upmann *et al.*, 2024*b* ▸) the structures of ten corresponding trihalogenido-gold(III) complexes. Further background material, including a more extensive summary of our previous results, can be found in Part 6 and is not repeated here. 
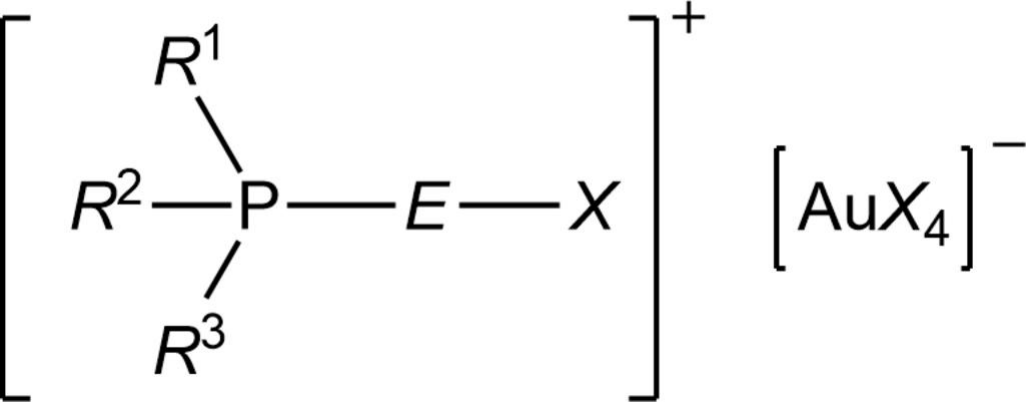


In this paper, we report the structures of fourteen (halochalcogenyl)tri­alkyl­phospho­nium tetra­halogenido­aurates(III) of general formula [^*t*^Bu*_3–n_^i^* Pr_*n*_P*EX*][Au*X*_4_]. In total there are sixteen possible permutations of *n* (0–3), the chalcogenide *E* (S or Se) and the halogen *X* (Cl or Br), but the two compounds with *n* = 0 and *E* = Se were not obtained. The chlorine derivatives are numbered **17a**–**23a** and the bromine derivatives **17b**–**23b**, following on from the gold(I) complexes **1**–**8** and the gold(III) complexes **9**–**16** in the previous papers (Upmann *et al.*, 2024*a* ▸,*b* ▸). Details of the composition of each compound studied are given in Table 1 ▸. The structures of **18a**, **19a**, **22a** and **23a** were briefly presented in a preliminary communication (Upmann & Jones, 2013 ▸), but have been re-refined using a much more recent version of *SHELXL* (2019 rather than 1997; Sheldrick, 2015 ▸) and are discussed in more detail here. We abstain from giving the systematic names for all these compounds; compound **21a**, for instance, is (chloro­selan­yl)triiso­propyl­phospho­nium tetra­chlorido­aurate(III), **18b** is (bromo­sulfanyl)(*tert*-but­yl)diiso­propyl­phospho­nium tetra­chlorido­aurate(III), and the others may be named analogously.

## 2. Structural commentary

**Figure 1:**
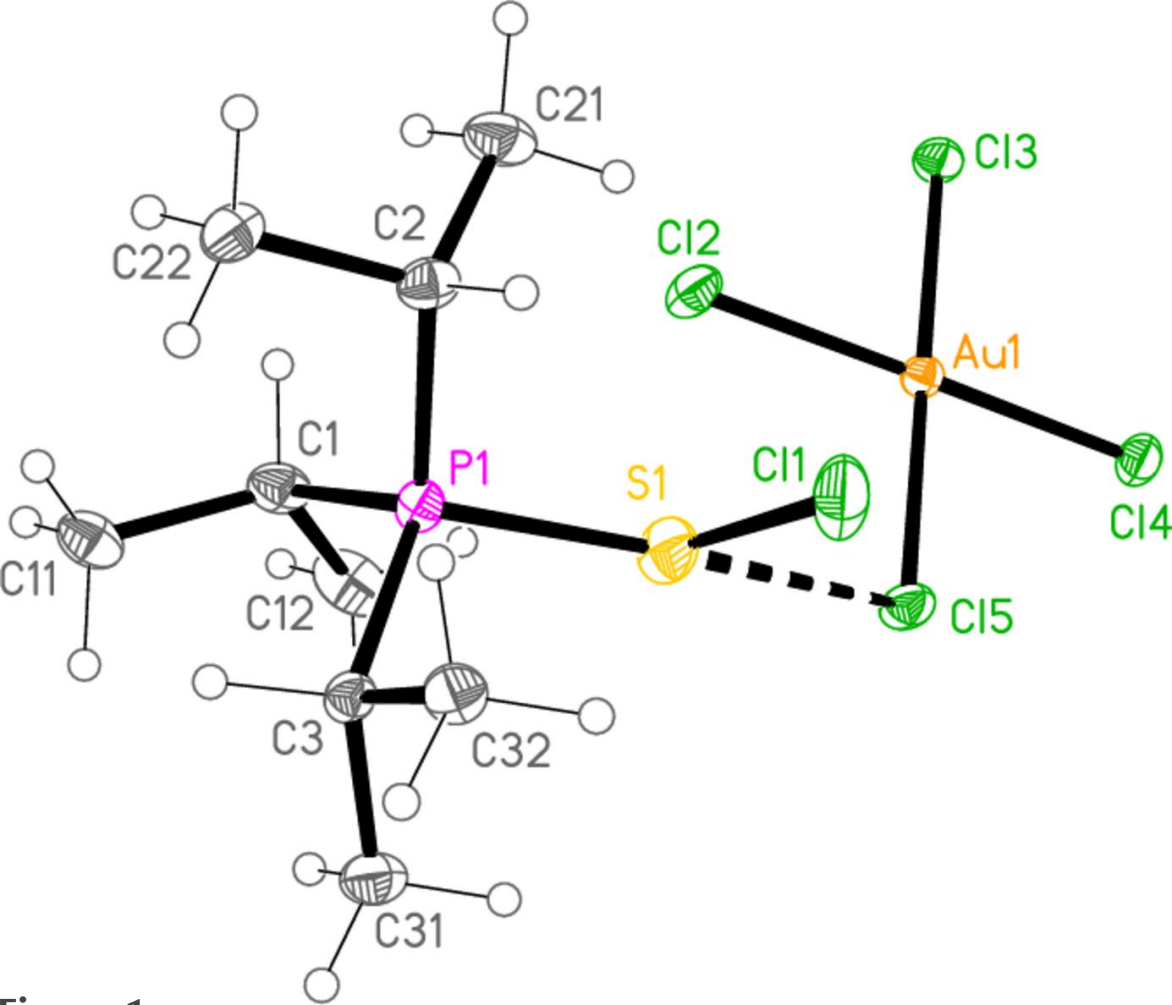
The structure of compound **17** ***a*** in the crystal. Ellipsoids represent 30% probability levels.

*General comments*: All compounds crystallized solvent-free and with *Z*′ = 1 (although all structures except **17a** contain two independent [Au*X*_4_]^−^ anions, each with crystallographic symmetry). The mol­ecular structures are shown in Figs. 1 ▸–14 ▸ ▸ ▸ ▸ ▸ ▸ ▸ ▸ ▸ ▸ ▸ ▸ ▸, with selected bond lengths and angles in Tables 2 ▸–15 ▸ ▸ ▸ ▸ ▸ ▸ ▸ ▸ ▸ ▸ ▸ ▸ ▸.

*Isotypy*: The four structures **18a**, **22a**, **18b** and **22b** form an isotypic set; the pairs **19a**/**23a**, **17b**/**21b**, and **19b**/**23b** are also isotypic. Within each set of isotypic structures, the *n* value is the same.

*Bond lengths and angles (1). [Au*X*_4_]^−^ anions*: For **17a**, the anion lies on a general position. For **19a** and **23a**, Au1 lies on a twofold axis and Au2 on an inversion centre, and the chlorine atoms at Au1 are disordered (with the atoms Cl3 and Cl4 being slightly displaced from the ideal positions on the twofold axis). All other anions lie with the gold atoms on inversion centres. The local symmetry approximates closely to the ideal 4/*mmm*, and there are no clear trends in the influence of the short *X*⋯*X* contacts on the Au—*X* bond lengths (see *Supra­molecular features*).

*Bond lengths and angles (2). (Halochalcogenyl)tri­alkyl­phospho­nium cations*: The P—S bond lengths lie in the range 2.0852–2.1035 Å, av. 2.0952 Å; the P—Se bond lengths are 2.2364–2.2557 Å, av. 2.2477 Å. These averages are slightly larger than in the trihalogenido-gold(III) complexes (2.0602 and 2.2183 Å), and may reasonably be regarded as corresponding to essentially single P—*E* bonds (for the mild controversy about the P—*E* bond order in phosphane chalcogenides, see *e.g*. Schmøkel *et al.*, 2012 ▸). The *E*—*X* bond lengths are: S—Cl = 2.023–2.0316 Å, av. 2.0291 Å; Se—Cl = 2.1645–2.1736 Å, av. 2.1678 Å; S—Br = 2.1934–2.2077 Å, av. 2.2004 Å; Se—Br = 2.3179–2.3255 Å, av. 2.3224 Å. For CCDC results on related bond lengths, see the *Database survey* below.

For each group of P—*E*—*X* bond angles, the values increase monotonically (with one extremely slight exception) with the steric bulk of the alkyl groups (P—S—Cl = 99.25–104.74°, P—Se—Cl = 98.38–100.40°, P—S—Br = 99.46–105.70°, P—Se—Br = 96.32–101.91°). For analogous S/Se pairs, the angles at Se are consistently smaller than those at S. For each cation, one of the alkyl groups (always a *t*-butyl group, if present) lies approximately anti­periplanar (‘*trans*’) to *X* in the atom sequence C—P—*E*—*X*; the central carbon atom of this group is consistently labelled C1. This behaviour, for which we see no clear explanation, contrasts with that of the gold(I) derivatives in Part 6, where the isopropyl group (where present, with two exceptions) occupied the *trans* position, and also with that of the gold(III) derivatives in Part 7, where the tendency of the isopropyl group to lie *trans* to *X* was overridden in all the ^*t*^Bu_2_^*i*^PrP*E* derivatives; we inter­preted this in terms of a greater importance of intra­molecular hydrogen bonds C—H_methine_⋯*X*. Selected dimensions of previously published halochalcogenyl­phospho­nium cations are shown in Table 16 ▸. There are some differences, *e.g.* the somewhat shorter Se—Cl bonds in this paper compared to the previous values, for which we also see no obvious explanation.

Contacts between the anion and cation of the asymmetric unit, as shown in Figs. 1 ▸–14 ▸ ▸ ▸ ▸ ▸ ▸ ▸ ▸ ▸ ▸ ▸ ▸ ▸, are discussed in the next section.

## 3. Supra­molecular features

For general aspects of packing and types of secondary inter­action, as applied to these compounds, a series of general articles are cited in our previous paper (Upmann *et al.*, 2024*a* ▸). Details of contacts are given in Tables 16 ▸–18 ▸ ▸, while hydrogen bonds are given in Tables 19 ▸–32 ▸ ▸ ▸ ▸ ▸ ▸ ▸ ▸ ▸ ▸ ▸ ▸ ▸; these include intra­molecular contacts (see above) and several borderline cases, and not all of them will be discussed. The corresponding symmetry operators, not given explicitly in the following discussion, may also be found in those Tables. In all packing diagrams presented here, hydrogen atoms not involved in hydrogen bonding are omitted for clarity, and the atom labels indicate the asymmetric unit. It is worth repeating the caveat that X-ray methods reveal short inter­molecular contacts, but not the corresponding energies, so that descriptions of mol­ecular packing in terms of particular secondary contacts must to some extent be subjective. Furthermore, there is no clear objective judgement, on the basis of contact lengths and angles, as to which contacts should be regarded as more important or less important for the packing. Finally, the exposed nature of the one-coordinate halogen atoms, taken together with the large number of hydrogen atoms, means that some short H⋯*X* contacts are inevitable. Nevertheless, it is possible to provide informative packing diagrams.

The most striking secondary inter­actions are the short halogen⋯halogen contacts between cation and anion; these are shown explicitly in Figs. 1 ▸–14 ▸ ▸ ▸ ▸ ▸ ▸ ▸ ▸ ▸ ▸ ▸ ▸ ▸ (except for compounds **17a**, **19a** and **23a**, for which instead chalcogen⋯halogen contacts are observed; see below). From our previous experiences, we expected this type of inter­action to be ubiquitous for these compounds, but this proved to be an erroneous assumption; only eleven of the fourteen structures display such *X*⋯*X* contacts, and we discuss these first. Selected contact dimensions are given in Table 17 ▸. The common features (exceptions are discussed below) are: (1) The *X*⋯*X* distance is generally shorter than the double van der Waals radius and in some cases extremely short, *e.g.* the Br⋯Br distance of 3.1509 (7) Å in [Ph_3_PSBr][AuBr_4_]. Being ‘softer’ than chlorine, bromine would be expected to form stronger (and thus shorter compared to the van der Waals distance) contacts. (2) The *E*—*X*⋯*X* grouping is approximately linear. (3) The *X*⋯*X*—Au angle is approximately 90°. (4) The relative orientation of the anion and cation is described by the torsion angle *X*⋯*X*—Au—*X*_cis_; this too is approximately 90° (positive for one *X_cis_* and negative for the other).

The combination of linear *E*—*X*⋯*X* and right-angled *X*⋯*X*—Au parallels the model for short contacts C—*X*⋯*X*—C in organic compounds, and both may be regarded as a form of ‘halogen bonding’ (see *e.g.* Metrangolo *et al.*, 2008 ▸). The strongest C—*X*⋯*X*—C inter­actions are termed ‘type II’ according to the classification of Pedireddi *et al.* (1994 ▸) and are characterized by C—*X*⋯*X* angles of approximately 180 and 90°. The simple model is that (as shown by calculation) there is a small region of positive charge δ+ in the direction extending one C—*X* vector, while the overall δ- charge of the other *X* atom (because of its higher electronegativity compared to carbon) is distributed perpendicular to the C—*X* bond (Legon, 2010 ▸). We are, however, not aware of similar calculations for atoms other than carbon.

The main exceptions to the above generalities involve the tri-*t*-butyl derivatives **20a** and **20b**, which show the narrowest *E*—*X*⋯*X* angles [159.83 (6) and 157.33 (4)°], the widest *X*⋯*X*—Au angles [115.19 (4) and 112.62 (2)°] and completely different absolute torsion angles *X*⋯*X*—Au—*X_cis_* of 8.88 (5) and 9.80 (2)° [paired with 171.12 (2) and 170.20 (2)° for the other *X_cis_* atom], so that these four atoms form a synperiplanar grouping. The compound [Ph_3_PSeBr][AuBr_4_] is also an outlier, with *X*⋯*X*—Au—*X*_cis_ values of 48.32 (1) and −131.68 (1)°.

The other three structures (**17a**, **19a** and **23a**) involve *E*⋯Cl contacts between the cation and the anion, as we had previously observed for [Ph_3_PSeCl]_2_[Au_4_Se_2_Cl_10_] (Upmann *et al.*, 2019 ▸), rather than halogen⋯halogen contacts. Details are given in Table 18 ▸. The P—*E*⋯Cl angles are approximately linear and the *E*⋯Cl—Au angles are somewhat larger than right angles, but there are no clear trends for the torsion angles *E*⋯Cl—Au—Cl_*cis*_. Since the *E* atoms presumably carry a partial positive charge, and the Cl atoms a partial negative charge, these *E*⋯Cl contacts may be termed as ‘chalcogen bonds’, a class of secondary contact named in analogy to ‘halogen bonds’. Other criteria also fulfil the requirements given by Aakeroy *et al.* (2019 ▸). The topic of chalcogen bonds has been reviewed by Vogel *et al*. (2019 ▸).

Of course other types of inter­molecular contact are also involved in the overall packing, which we now describe. The decision as to which contacts are more or less important is, as remarked above, to some extent subjective; a packing pattern is more assimilable to the human eye if it involves a small number of contacts, and we have tended to choose heavy-atom contacts rather than possible hydrogen bonds. Where the latter are considered, we tend to concentrate on the inter­actions of the methine rather than the methyl hydrogens; we established in the series of *R* ^1^ *R* ^2^ *R* ^3^P*E*Au*X*_3_ complexes that the methine hydrogen atoms have a greater tendency than methyl hydrogens to form ‘weak’ hydrogen bonds, thereby influencing the mol­ecular conformation by forming intra­molecular H⋯*X* hydrogen bonds.

Dance (2003 ▸) has pointed out (our paraphrasing) that packing energies are probably determined in many cases by a large number of slightly favourable inter­actions, such as H⋯H van der Waals contacts, rather than a small number of very short contacts (of which the shortest may even be unfav­ourable in energy terms). Despite this, it is probably inevit­able that one will (over)emphasize the contacts between heavy atoms in order to make the diagrams more inter­pretable. Packing diagrams based on the extremely numerous weakly attractive H⋯H inter­actions would be neither easily drawn nor easily inter­preted.

For compound **17a**, the S⋯Cl inter­action combines with two hydrogen bonds from the methine hydrogens H1 and H3 to form a layer structure parallel to ( 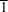 01) (Fig. 15 ▸). The atom H2 makes a short intra­cationic contact to Cl1, but the angle is necessarily very narrow.

**Figure 2:**
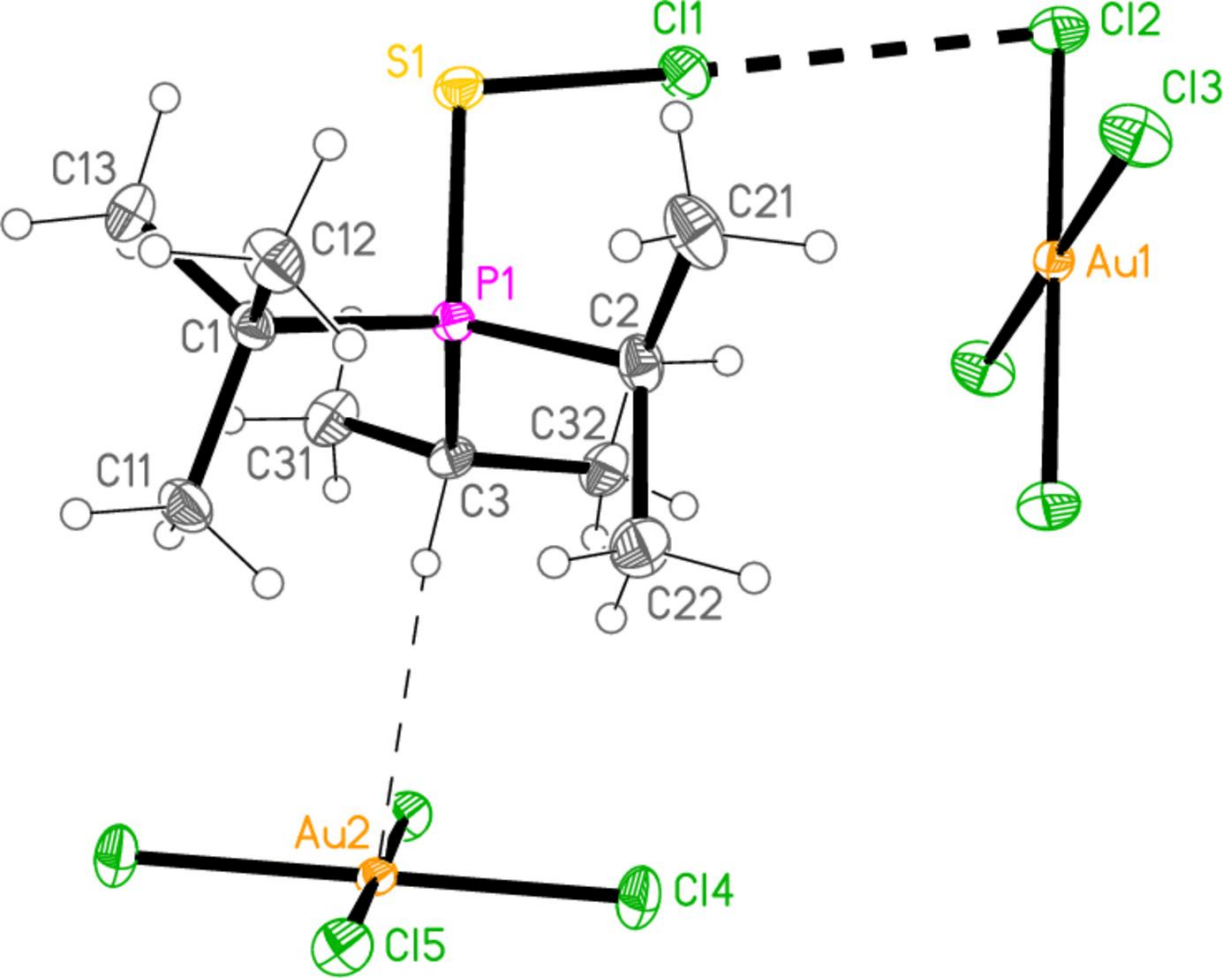
The structure of compound **18** ***a*** in the crystal. Ellipsoids represent 50% probability levels.

The packing of compound **18a** may be described in terms of three secondary inter­actions: the short Cl1⋯Cl2 contact, an unusually short contact C3—H3⋯Au2 (H⋯Au = 2.75 Å) that could be classified as a hydrogen bond (Schmidbaur *et al.*, 2014 ▸ and Schmidbaur, 2019 ▸) and another strikingly short contact, Cl5⋯Cl5(2 − *x*, 1 − *y*, −*z*) = 3.2632 (10) Å, between anions. The angle Au2—Cl5⋯Cl5’ is 149.86 (3)°. We have drawn attention to such short contacts between [Au*X*_4_]^−^ anions elsewhere (Döring & Jones, 2016 ▸). The first two contacts lead to the formation of zigzag chains with overall direction parallel to [10 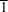 ] (Fig. 16 ▸), further linked by the contact S1⋯Cl2(−*x*, −*y*, 1 − *z*) = 3.5768 (8) Å; the second and third contacts link cations and Au2 anions parallel to the *a* axis (Fig. 17 ▸). The contact H2⋯Cl2′ between the cation and a symmetry-extended anion is rather long at 2.93 Å; it can be recognized in Fig. 2 ▸, but is not drawn explicitly there, and is not included in the packing diagram.

Compounds **18a**, **18b**, **22a** and **22b**, with all possible permutations of *E* = S/Se and *X* = Cl/Br, are isotypic, and so it should not be necessary to provide separate packing diagrams. However, this set of compounds provides a good opportunity to consider the definition of ‘isotypic’ and the subjectivity of packing diagrams. For **18a**, we considered the S1⋯Cl2 contact to be significant, but did not include the significantly longer contact S1⋯Cl3(1 − *x*, −*y*, 1 − *z)*, 3.7012 (8) Å, regarding it (arbitrarily) as too long. The corresponding contacts (in Å; same operator as above) for the other compounds are: **18b**, S1⋯Br2 = 4.0635 (10) and S1⋯Br3 = 3.6779 (11); **22a**, Se1⋯Cl2 = 3.4602 (7) and Se1⋯Cl3 = 3.7004 (8); **22b**, Se1⋯Br2 = 3.7704 (6) and Se1⋯Br3 = 3.7484 (6). We draw attention to the considerable variations in the lengths of these contacts; thus S1⋯Br2 might well be ignored for **18b**, while S1⋯Br3 is the shorter and thus more significant contact. For **22a**, Se1⋯Cl2 is the much shorter inter­action, whereas for **22b** the lengths are almost equal. For **22b**, one can then draw an alternative packing diagram excluding the anion based on Au2, showing both Se⋯Br contacts (Fig. 18 ▸). The residues are linked by these contacts and by Br1⋯Br2 to provide crosslinked chains of anions and cations parallel to [1 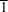 0], forming a layer structure parallel to the *ab* plane, in which ten-membered rings Au_2_Se_2_Br_6_ can be recognized.

The potential hydrogen bond H2⋯Cl2′, ignored for **18a** (as discussed above) as being ‘too long’ at 2.93 Å, also behaves somewhat differently for the other three isotypic structures: for **18b** H2⋯Cl2′ is shorter at 2.87 Å, whereas for the two bromo derivatives **18b** and **22b** the H2⋯Br2′ contacts are very long at 3.28 and 3.17 Å, respectively. This again draws attention to possible significant differences between isotypic structures and to the arbitrary nature of decisions to include or exclude particular contacts in the discussion of the packing. During the preparation of this paper, Bombicz (2024 ▸) published a commentary ‘What is isostructurality?’, in which she raised similar questions, commenting for instance that ‘The extent of the difference between corresponding crystal structures referred to as isostructural is not limited’, and that the IUCr definition of ‘isostructural/isotypic’ (regarded as synonymous terms) is vague on this point.

**Figure 3:**
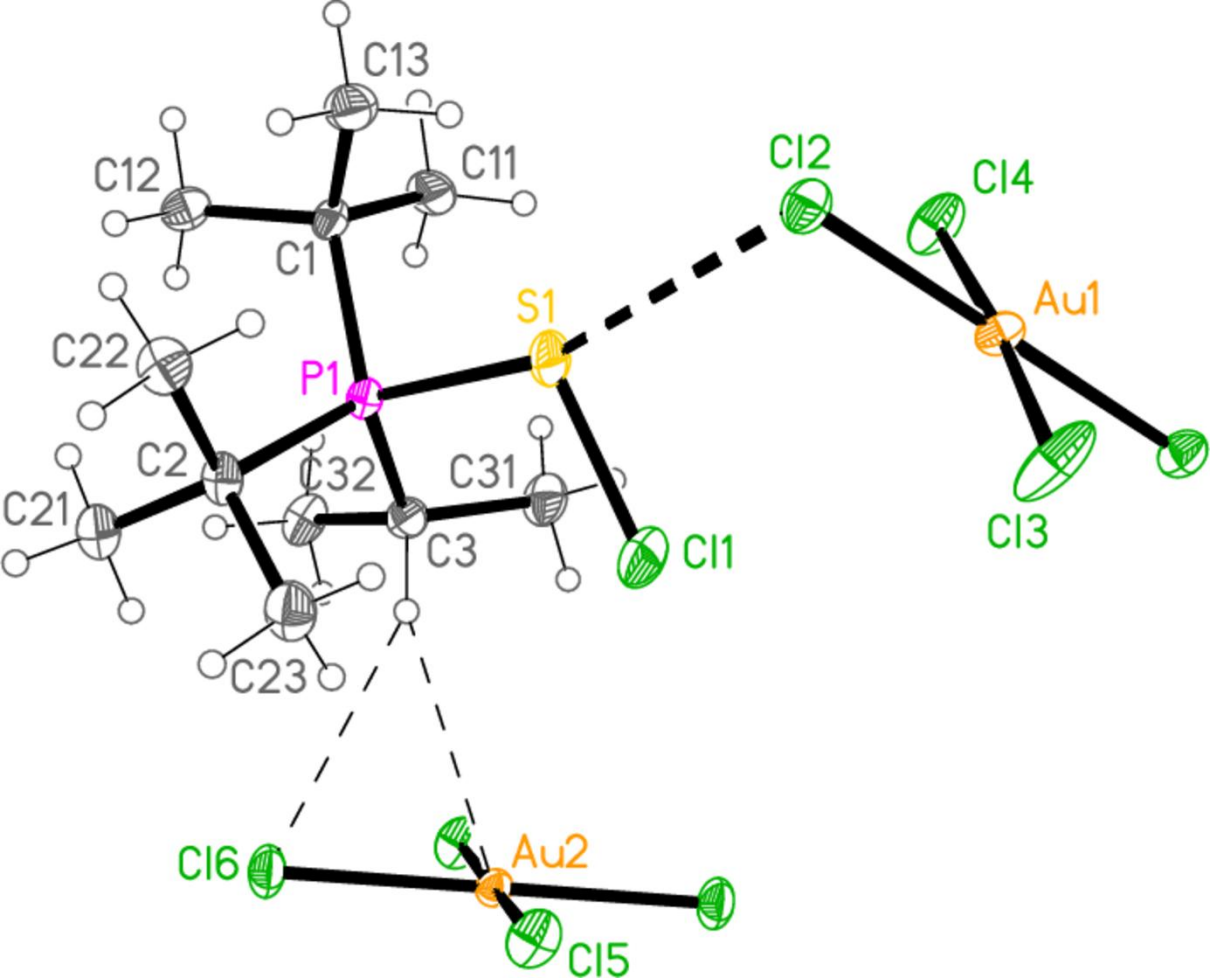
The structure of compound **19** ***a*** in the crystal. Ellipsoids represent 50% probability levels. Only one position of the disordered atoms Cl3 and Cl4 is shown.

Compound **19a** and the isotypic **23a** (as discussed above, see Figs. 3 ▸ and 7 ▸) involve *E*⋯Cl rather than Cl⋯Cl inter­actions between the cation and one anion (based on Au1); the other anion (based on Au2) is close to the methine hydrogen H3, with H3⋯Au2 and H3⋯Cl6 distances of 2.93, 2.90 Å for **19a** and 2.95, 2.88 Å for **23a** that might be inter­preted as three-centre hydrogen bonds. The main feature of the packing (as shown in Fig. 19 ▸ for **23a**) is the formation of almost square networks of [AuCl_4_]^−^ anions, involving Au1, parallel to the *bc* plane in the regions *x* ≃ 0, 0.5, within which the cations are linked *via E*⋯Cl contacts. The contact distances (Å) are S1⋯Cl4(1 − *x*, *y*, 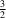 − *z*) = 3.80, Cl3⋯Cl4(*x*, 1 + *y*, *z*) = 3.30, Cl2⋯Cl2(1 − *x*, 2 − *y*, 1 − *z*) = 3.633 (2) for **19a** and Se1⋯Cl4 = 3.77, Cl3⋯Cl4 = 3.28, Cl2⋯Cl2 = 3.6717 (12) for **23a** (note however that the disorder of Cl3 and Cl4 make these values uncertain; in Fig. 19 ▸, the idealized positions on the twofold axis are used for clarity, and these are the basis for the calculated distances). The [AuCl_4_]^−^ anions involving Au2 occupy the regions at *x* ≃ 0.25, 0.75.

Compound **20a** forms winding chains parallel to the *c* axis (Fig. 20 ▸); residues are linked by the inter­ionic Cl1⋯Cl2 contact and a double contact from S1 to two chlorine atoms of a neighbouring anion [S1⋯Cl4 = 3.6661 (14), S1⋯Cl5 = 3.5471 (14) Å], whereby the corresponding S1⋯Au2 distance is necessarily short at 3.7113 (9) Å. Chains are linked by several H⋯Cl contacts.

In the packing of compound **21a**, two Se⋯Cl contacts [Se1⋯Cl5 = 3.3504 (6) and Se1⋯Cl5(2 − *x*, 1 − *y*, 1 − *z*) = 3.3728 (5) Å], which form striking Se_2_Cl_2_ quadrilaterals, combine with the long Cl1⋯Cl2 contact, 3.6031 (7) Å, and two H_methine_⋯Cl hydrogen bonds to symmetry-extended anions to form a layer structure parallel to (10 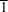 ) (Fig. 21 ▸). The quadrilaterals are thereby linked directly by [AuCl_4_]^−^ anions involving Au2, parallel to the *b* axis, and indirectly *via* [AuCl_4_]^−^ anions involving Au1, parallel to [111]. The methine hydrogen H1 makes a short contact to Cl4′ (2.78 Å), but also a longer contact of 2.93 Å to the neighbouring Cl5 atom; this may be regarded as an asymmetric three-centre system, but the longer contact is omitted from the Figures. The third methine hydrogen, H3, also makes a longer contact of 2.94 Å to Cl2(*x*, 1 + *y*, *z*); this contact also lies in the layer but is omitted for clarity.

The packing of **17b** only has one strikingly short contact, Br1⋯Br2 = 3.3206 (5) Å between anion and cation, but there is also a three-centre grouping with Br4⋯Br1 = 3.8725 (5) and Br4⋯S1 = 3.7666 (8) Å, also within the asymmetric unit. Additionally, the borderline contacts Br1⋯Br2(2 − *x*, 1 − *y*, −*z*) = 3.8652 (5), Br3⋯Br4(−1 + *x*, *y*, *z*) = 3.8725 (5) and Br5⋯Br5(2 − *x*, −1 − *y*, 1 − *z*) = 3.8808 (6) Å link the residues to form layers parallel to the *ab* plane (Fig. 22 ▸). The two possible weak hydrogen bonds H1⋯Br5 and H3⋯Br3, quite long but reasonably linear, also lie within these layers, but are omitted from Fig. 22 ▸ for clarity. The isotypic structure **21b** has a much shorter corresponding Se1⋯Br4 contact of 3.6501 (5) Å.

**Figure 10:**
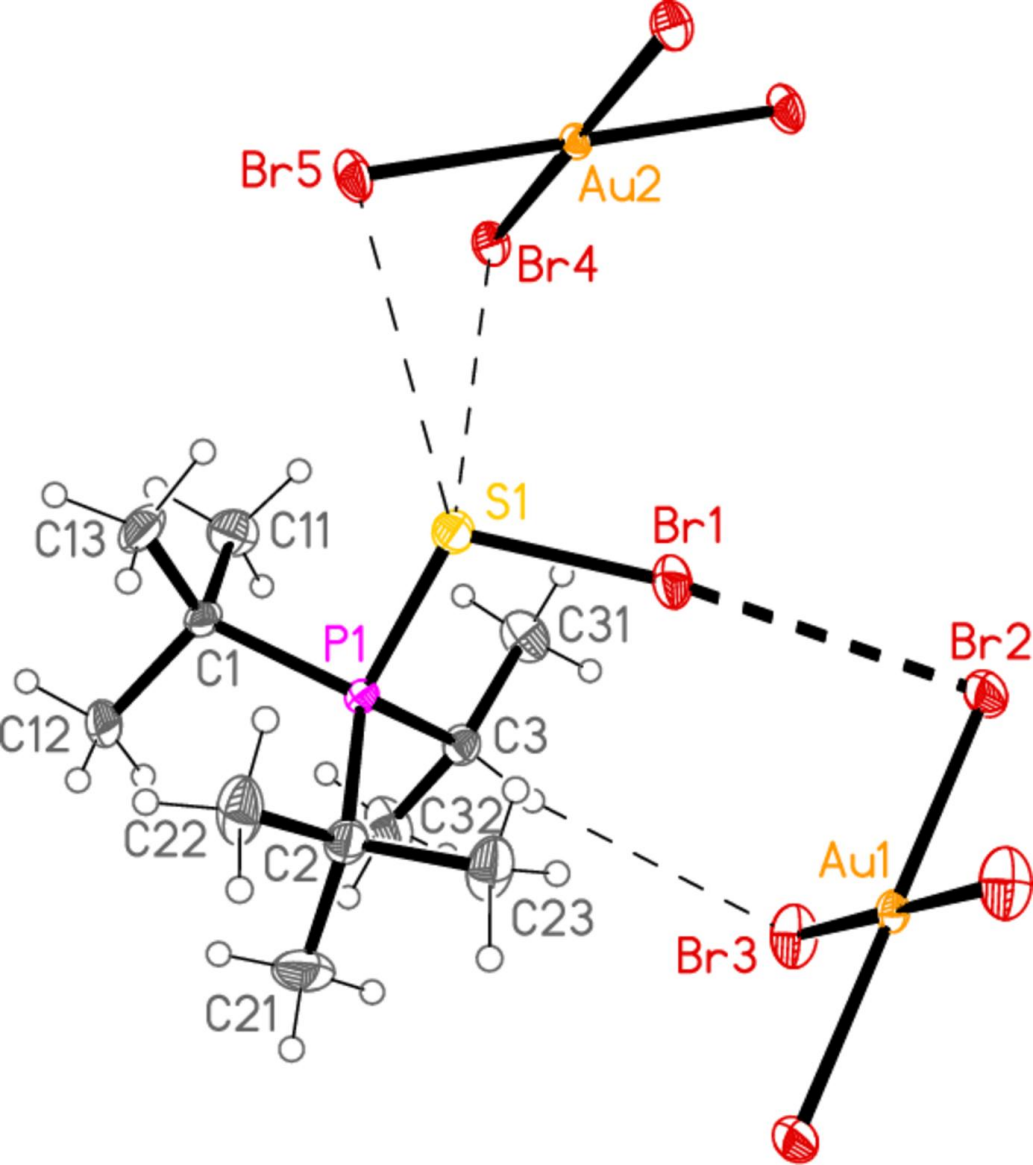
The structure of compound **19** ***b*** in the crystal. Ellipsoids represent 50% probability levels.

**Figure 15:**
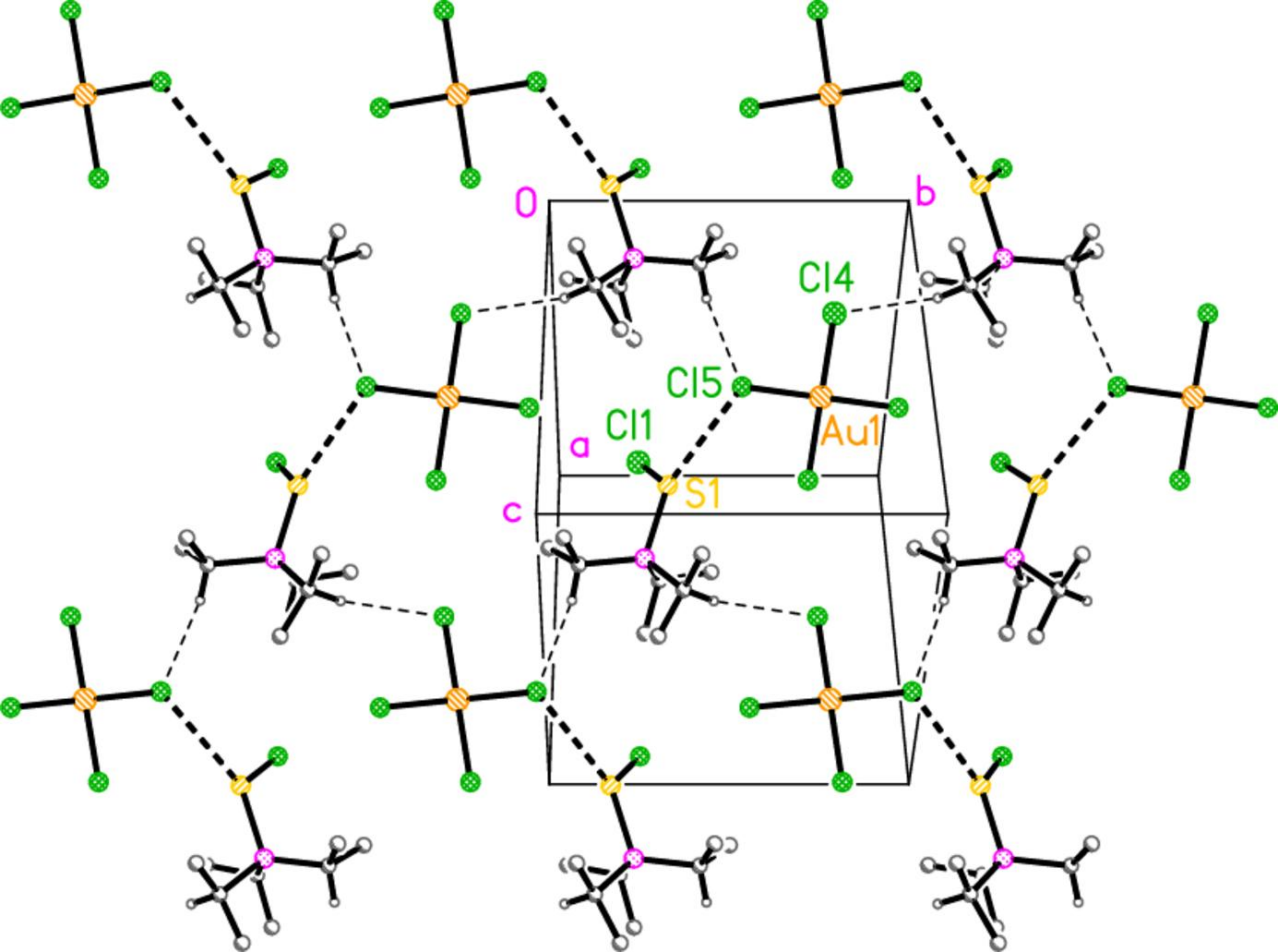
Packing diagram of **17** ***a***. Two H⋯Cl contacts combine with the S⋯Cl inter­actions to form a layer structure parallel to ( 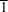 01); the viewing direction is perpendicular to this plane. The dashed lines indicate S⋯Cl inter­actions (thick) or H⋯Cl contacts (thin). For all packing diagrams, atom labels correspond to the asymmetric unit, and hydrogen atoms not involved in hydrogen bonds are omitted for clarity.

**Figure 16:**
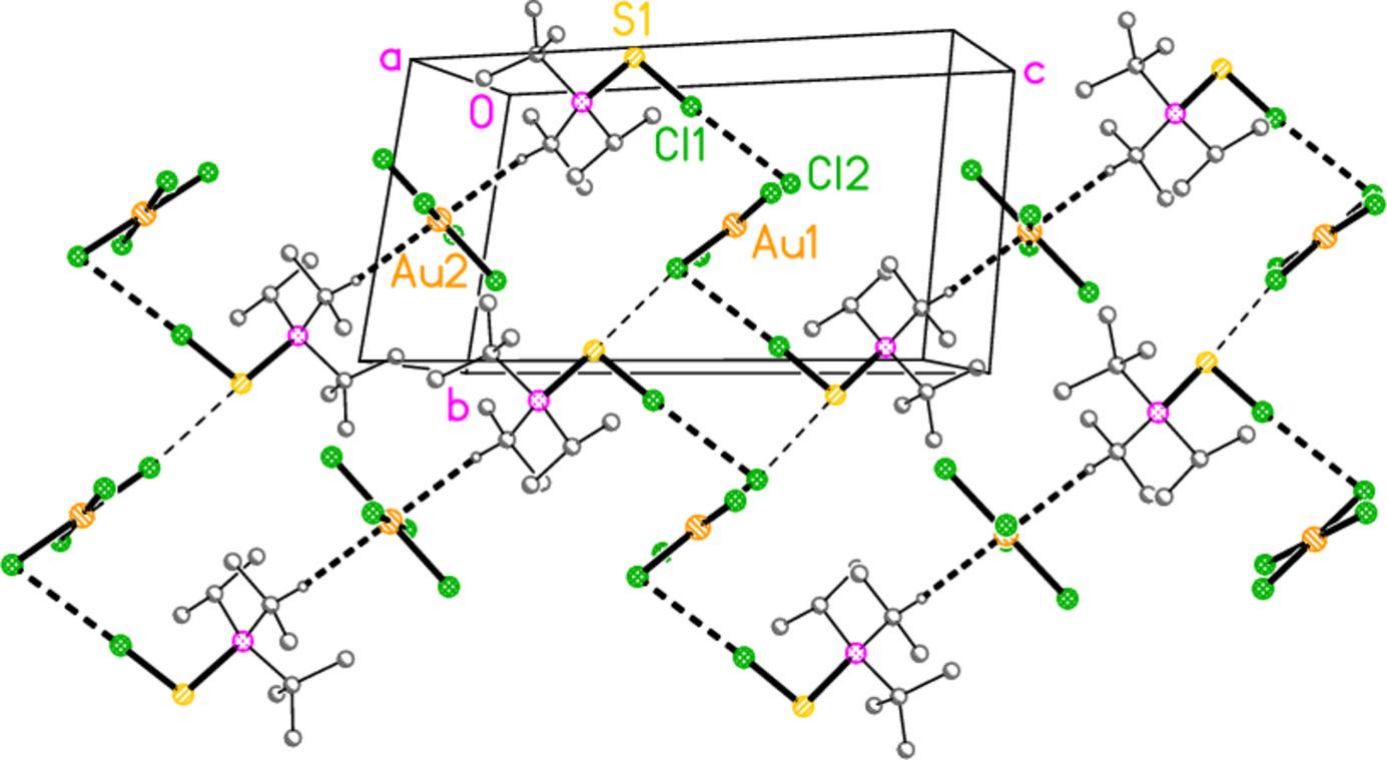
Packing diagram of **18** ***a*** viewed perpendicular to (212), showing two zigzag chains of residues (running horizontally). Dashed lines indicate Cl⋯Cl and H⋯Au contacts (thick) or S⋯Cl contacts (thin).

**Figure 17:**
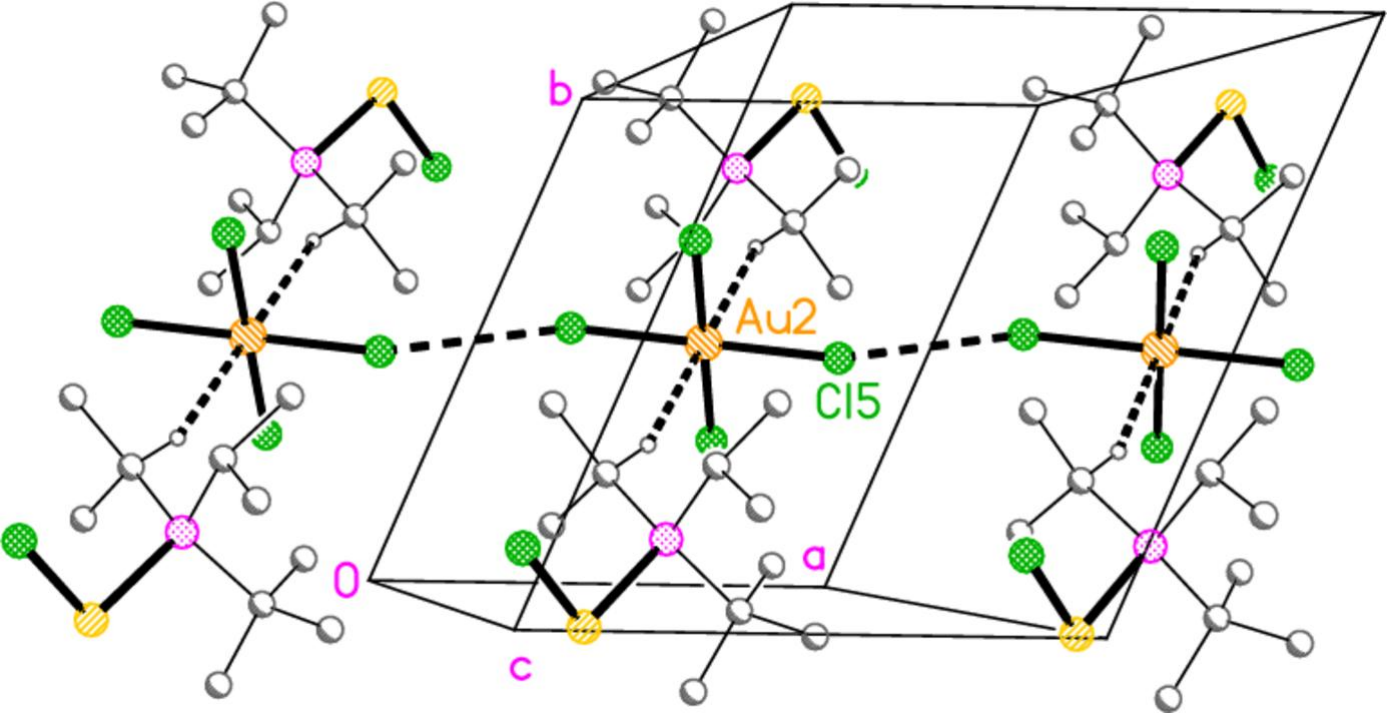
Packing diagram of **18** ***a*** viewed perpendicular to the *xy* plane in the region *z* ≃ 0. Dashed lines indicate Cl⋯Cl and H⋯Au contacts.

**Figure 18:**
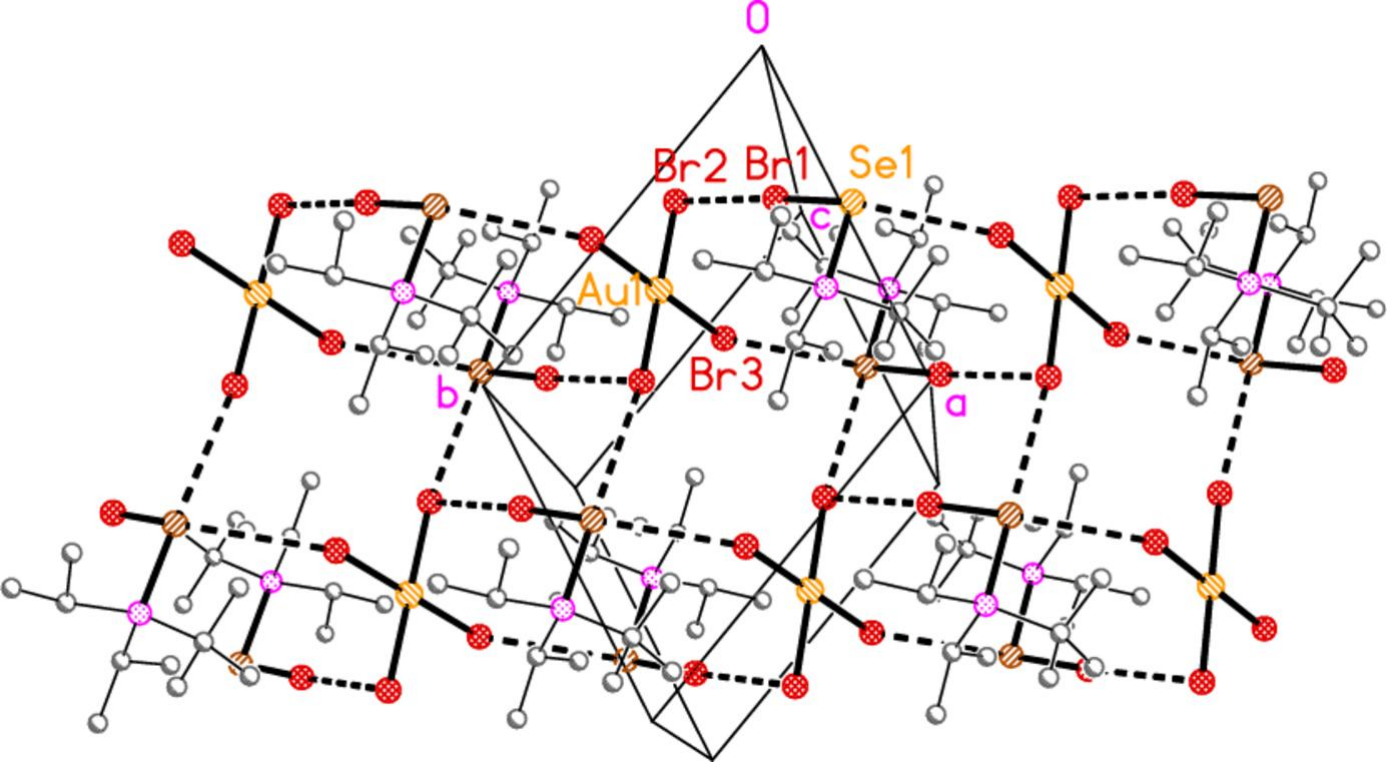
Packing diagram of **22** ***b*** viewed perpendicular to the *ab* plane. Hydrogen atoms and the anions based on Au2 are omitted. Residues are linked by Br⋯Br and Se⋯Br contacts (thick dashed lines) to form linear arrays (parallel to [1 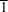 0], horizontal in the diagram), further crosslinked to form a layer.

**Figure 19:**
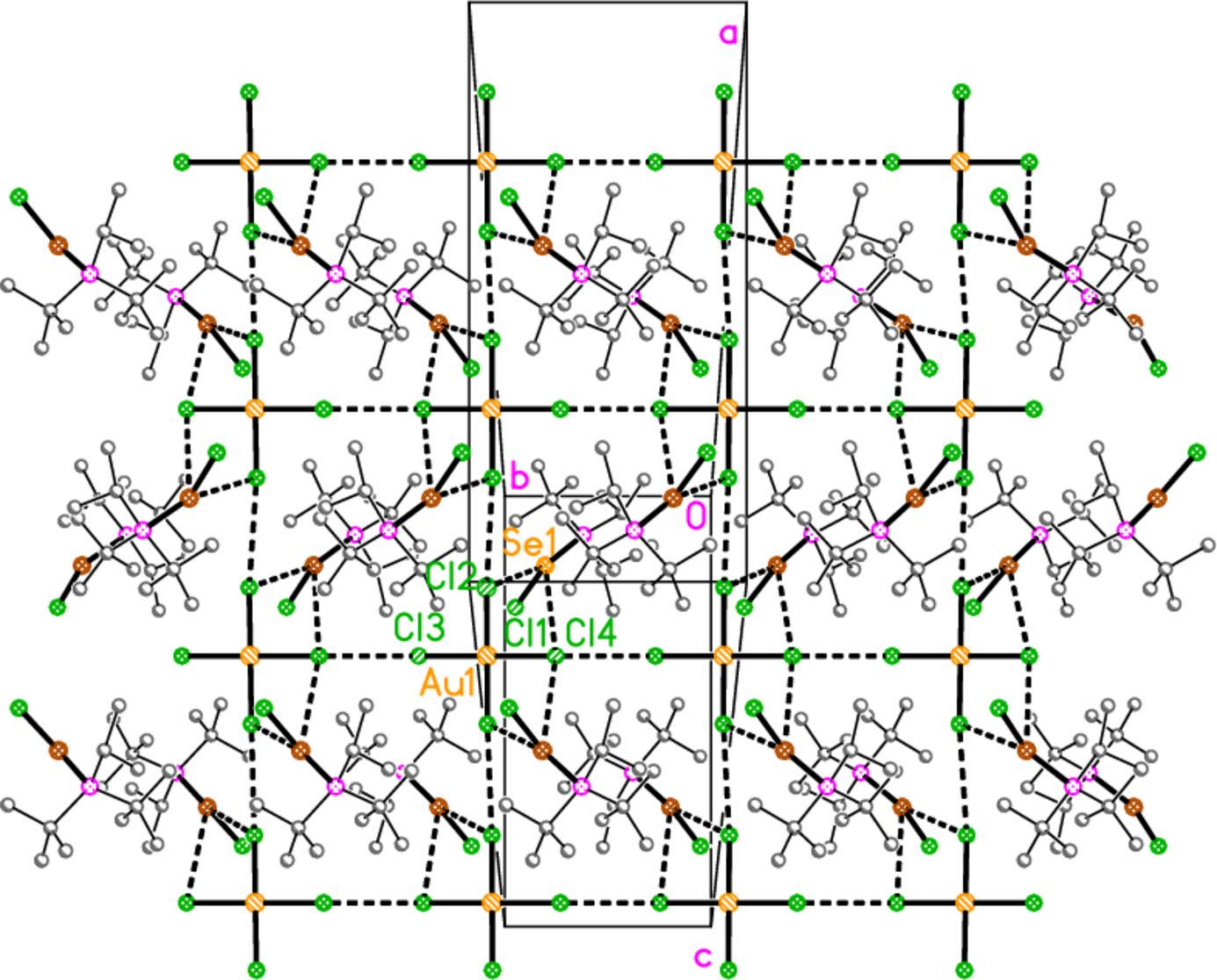
Packing diagram of **23** ***a*** viewed perpendicular to the *bc* plane in the region *x* ≃ 0.5. Hydrogen atoms and the anion based on Au2 are omitted. For clarity, the positions of the disordered atoms Cl3 and Cl4 are idealized to lie on the twofold axes. Thick dashed lines indicate Cl⋯Cl or Se⋯Cl contacts.

**Figure 20:**
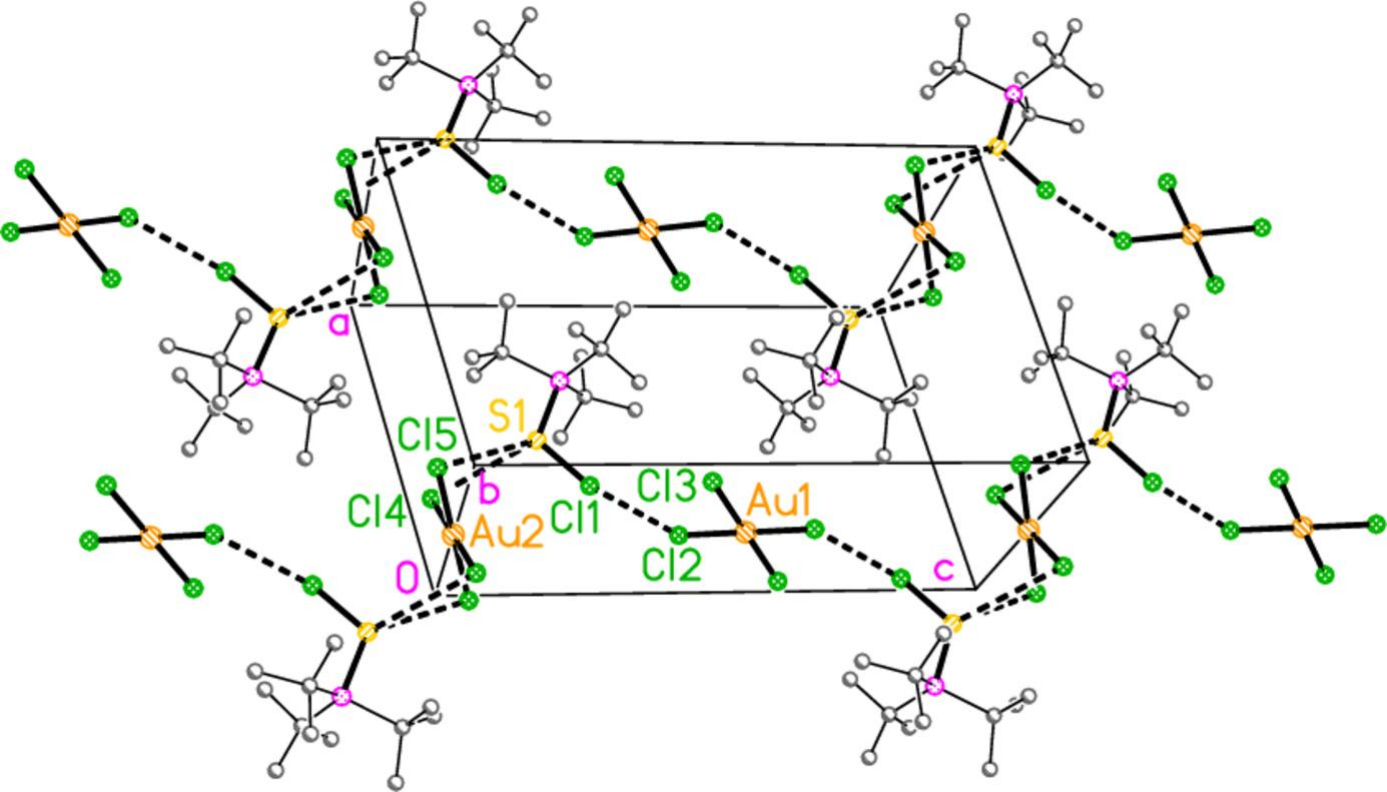
Packing diagram of **20** ***a***. The viewing direction has been rotated by *ca* 20° and 10° respectively around the horizontal and vertical directions from the direction parallel to the *b* axis (to avoid overlap of the two S⋯Cl contacts). Hydrogen atoms are omitted for clarity. Dashed lines indicate Cl⋯Cl and S⋯Cl contacts. The unusual orientation of the tetra­chloro­aurate(III) anion at Au1, with an approximately synperiplanar grouping Cl1⋯Cl2—Au1—Cl3, can be recognized.

**Figure 21:**
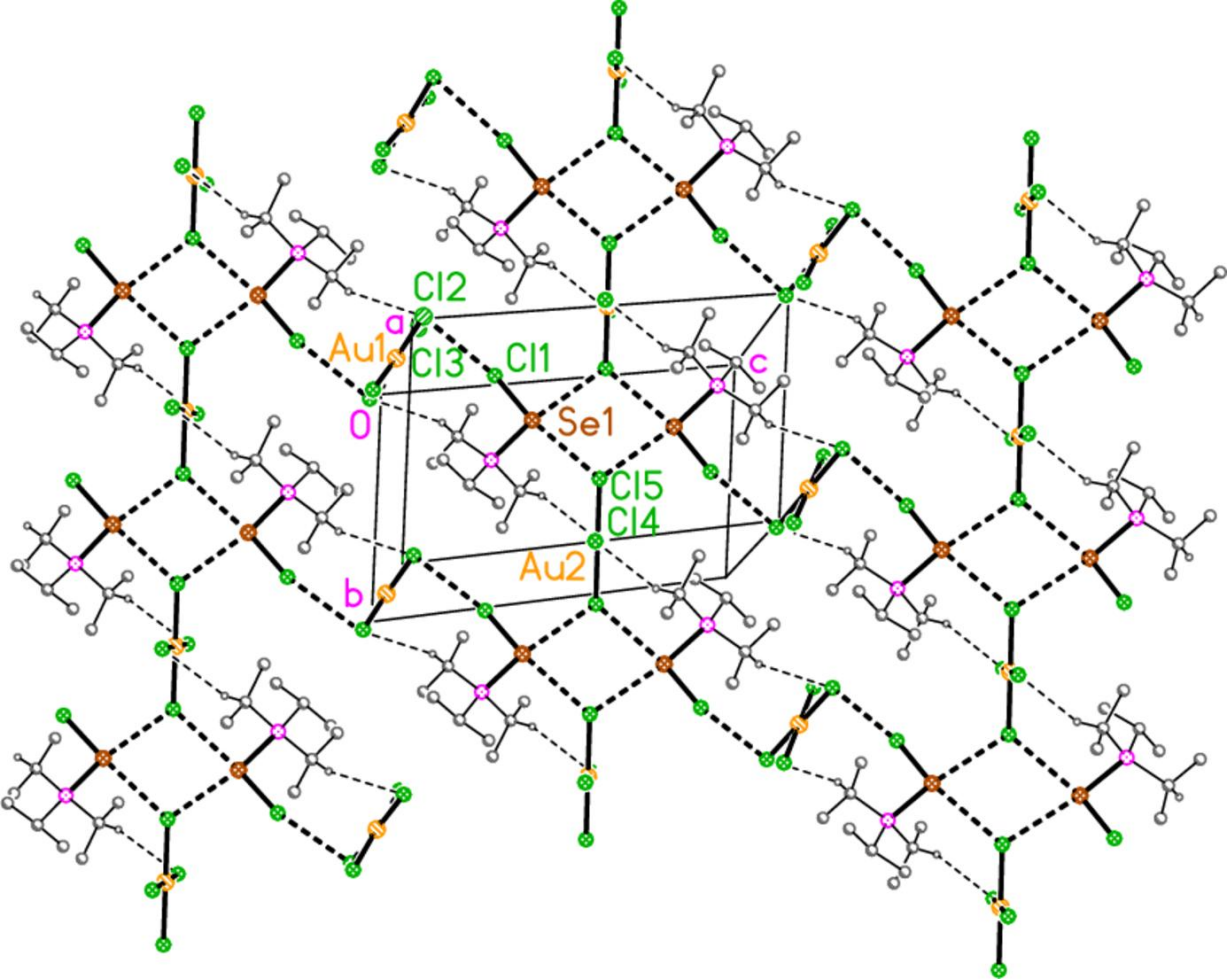
Packing diagram of **21** ***a***, viewed perpendicular to (10 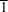 ). Dashed lines indicate Cl⋯Cl and Se⋯Cl contacts (thick) or H_methine_⋯Cl hydrogen bonds (thin). Note that the set of AuCl_4_^−^ anions based on Au2 is seen edge on, whereby the gold atoms are obscured.

**Figure 22:**
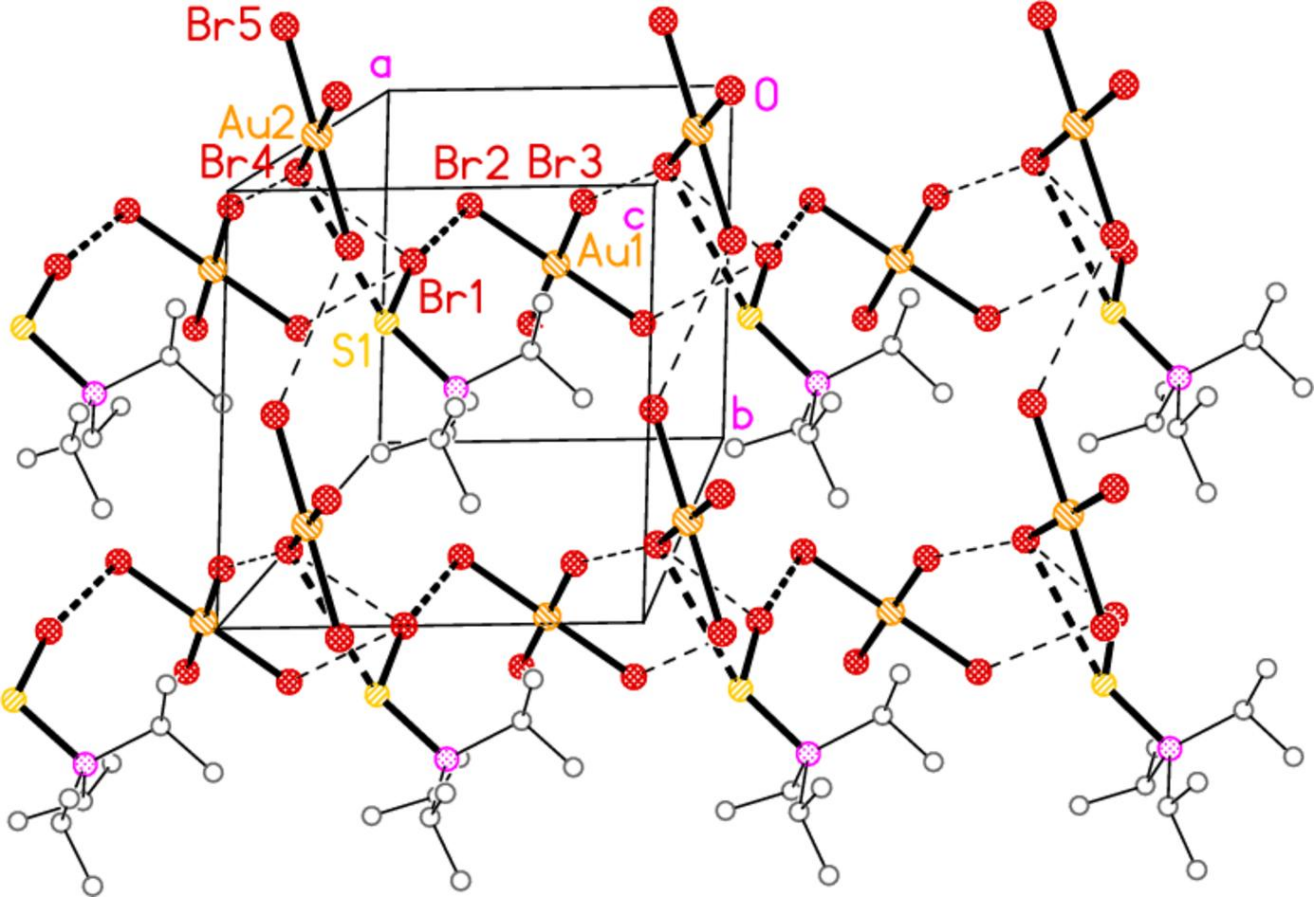
Layer structure of **17** ***b*** viewed perpendicular to the *ab* plane in the region *z* ≃ 0.25. Dashed lines indicate contacts S1⋯Br4 and Br1⋯Br2 (thick) or other appreciably longer Br⋯Br contacts (thin).

**Figure 23:**
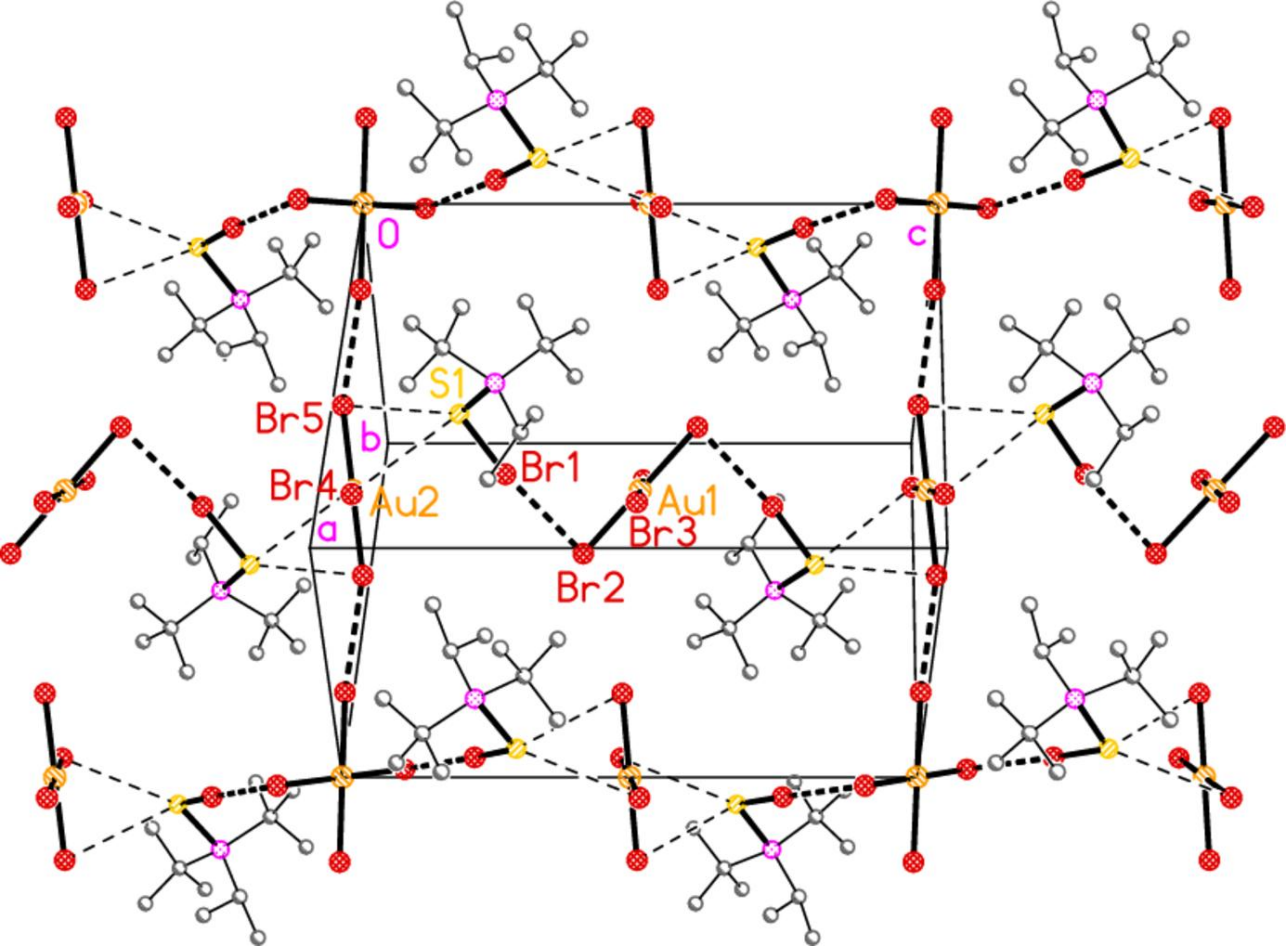
Layer structure of **19** ***b*** viewed perpendicular to (1 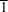 0). Dashed lines indicate short Br⋯Br contacts (thick) or rather longer S⋯Br contacts (thin). The anions based on Au2 are viewed edge-on, so that the atom Au2 is obscured by Br4. The corresponding anion at the right-hand edge of the cell is shown more clearly. The probable hydrogen bonds H3⋯Br3 (see text and Fig. 10 ▸) are omitted for clarity.

For compound **19b**, there are two very short Br⋯Br contacts, namely Br1⋯Br2 = 3.2686 (7) Å between cation and anion and Br3⋯Br5( 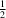 + *x*, 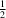 − *y*, 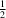 + *z*) = 3.3324 (6) Å between the two anions. These are accompanied by the three-centre association of S1⋯Br4 = 3.7797 (13) and S1⋯Br5 = 3.6326 (12) Å and the probable hydrogen bond H3⋯Br3 (Fig. 10 ▸), all within the asymmetric unit, to form a layer structure parallel to (1 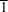 0) (Fig. 23 ▸). The corresponding distances for the isotypic **23b** are Br1⋯Br2 = 3.3416 (11), Br3⋯Br5 = 3.3205 (10), Se1⋯Br4 = 3.8310 (11) and Se1⋯Br5 = 3.5473 (10) Å.

**Figure 24:**
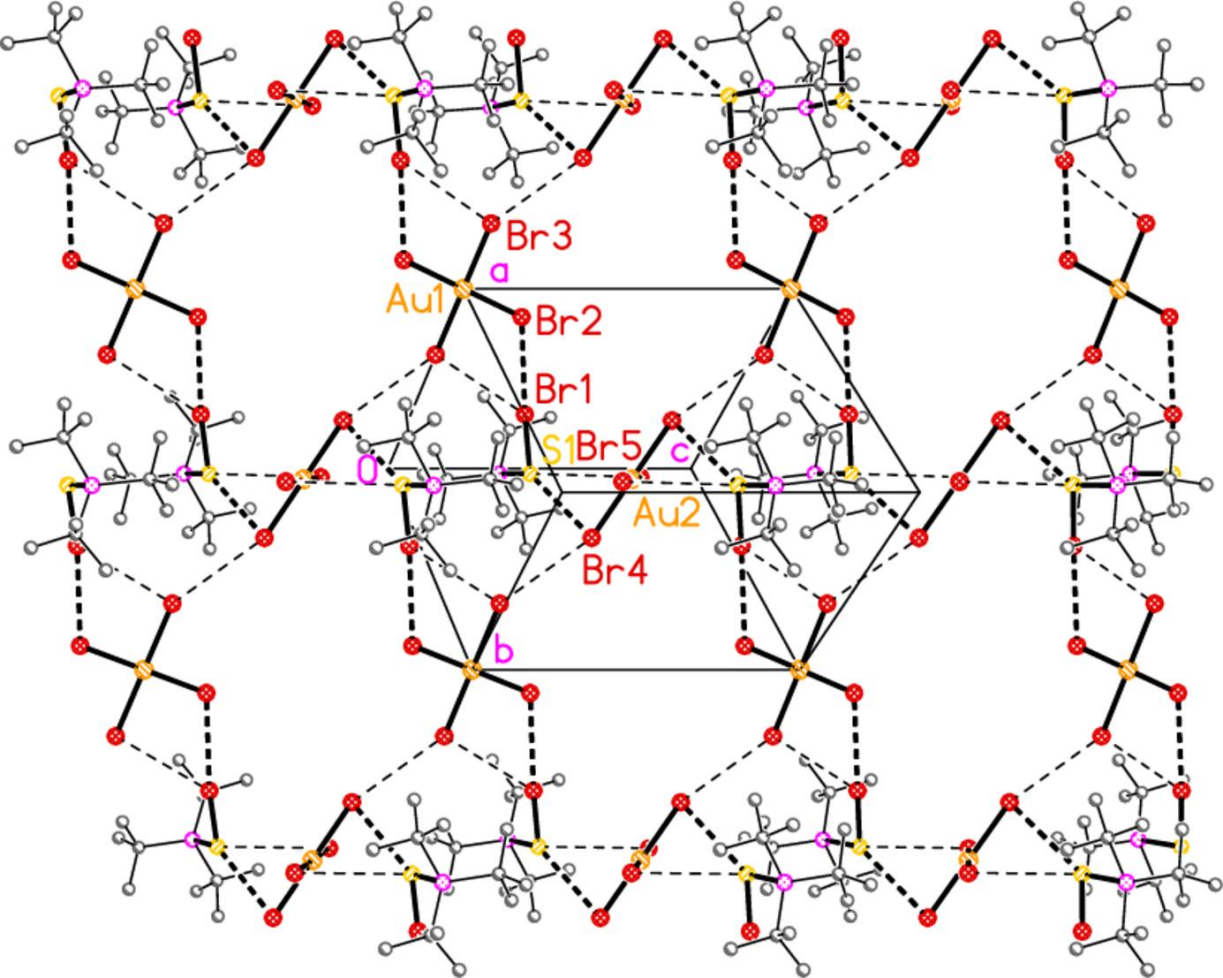
Layer structure of **20** ***b*** viewed perpendicular to (110). Dashed lines indicate short (thick) or long (thin) Br⋯Br and S⋯Br contacts. The horizontal direction is [001] and the vertical direction [1 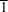 0]. The anions based on Au2 are viewed edge-on, so that the atom Au2 is obscured by Br5.

Compound **20b** has three Br⋯Br contacts: Br1⋯Br2 = 3.3465 (7) Å, between anion and cation, is very short, whereas the other two, Br1⋯Br3(2 − *x*, −*y*, −*z*) = 3.7995 (7) and Br3⋯Br4(1 + *x*, −1 + *y*, *z*) = 3.8361 (7) Å are long. There are also two S⋯Br contacts, one short and one long, namely S1⋯Br4 = 3.5208 (13) and S1⋯Br5(1 − *x*, 1 − *y*, 1 − *z*) = 3.8555 (13) Å. These combine to form a layer structure parallel to (110) (Fig. 24 ▸).

## 4. Database survey

The searches employed the routine ConQuest (Bruno *et al.*, 2002 ▸), part of Version 2022.3.0 of the Cambridge Structural Database (Groom *et al.*, 2016 ▸).

Except for the structures in our previous work, no hits were registered for the atom sequence P—(S or Se)—(Cl or Br) with coordination numbers restricted to 2 for S/Se and 1 for Cl/Br (but with unrestricted bond orders). To obtain usable values for the *E*—*X* single bond lengths, we therefore searched for the sequence C—(S or Se)—(Cl or Br), on the principle that phospho­rus and carbon are in many respects similar (see *e.g.* Dillon *et al.*, 1998 ▸). We obtained average values of 2.021, 2.224, 2.201 and 2.334 Å for S—Cl (20 values), S—Br (11 values), Se—Cl (12 values) and Se—Br (10 values) bonds, respectively. These are in reasonable agreement with our values given above. Care was, however, necessary in inter­preting the search output, because several hits with short intra­molecular contacts (some as short as 2.0 Å for Se⋯O, but not marked as bonds in the Database) involved three-coordinate selenium and, hence, needed to be removed. Thus the compound chloro­(8-(*N*,*N*-di­methyl­amino)­naphthyl­selenium(II) (Panda *et al.*, 1999 ▸; refcode LIWYIZ), with an intra­molecular Se—N distance of 2.174 (5) Å and a long Se—Cl bond *trans* to the N atom, was drawn as a formula without an Se—N bond (and therefore, not unreasonably, coded accordingly in the CCDC). The distance was described as ‘non-bonded’, but the displacement ellipsoid plot included this as a normal bond. There seems to be some confusion in the older literature as to what constitutes a bond in such systems.

Similarly, a search for the atom sequence C_3_P—S—C, with coordination numbers restricted to 4 for P and 2 for S, gave 19 hits and 21 P—S bond lengths, averaging to 2.076 Å. The analogous search using selenium gave 24 hits and 35 P—Se bond lengths, with an average of 2.233 Å. These are slightly shorter than our average values given above.

## 5. Synthesis and crystallization

For several of the compounds, the syntheses can be found in the PhD thesis of D. Upmann (Upmann, 2015 ▸). There was however a general problem of incomplete reactions, despite the use of excess oxidants PhICl_2_ or Br_2_. The following attempted, but not entirely successful, syntheses do not appear there:

Compound **17a**: 212 mg (0.5 mmol) of ^*i*^Pr_3_PSAuCl and 344 mg (1.25 mmol) of PhICl_2_ were each dissolved in 10 mL of di­chloro­methane. The solutions were combined and stirred for 30 min, during which time the solution changed from red to yellow; the solvents were then removed under vacuum. All attempts to isolate the product from this solution proved to be unsuccessful because intra­ctable oils or gums were formed; drying and recrystallization generally led to the separation of unidentified insoluble solids. ^31^P-NMR (81 MHz, CDCl_3_, 300 K): δ [ppm] 92.56, ^1^ *J*_P–Se_ 416.3 Hz. Despite the failure to prepare the substance in qu­antity, a few single crystals (coated with an adhesive gum) were obtained by overlaying a solution in di­chloro­methane with *n*-pentane (or diethyl ether) and storing it in a refrigerator (276 K) over the weekend.

Compound **20a**: Pilot experiments suggested that a large excess of PhICl_2_ was necessary for complete reaction. 91.8 mg (0.197 mmol) of ^*t*^Bu_3_PSAuCl and 541 mg (1.97 mmol) of PhICl_2_ were each dissolved in 3 mL of di­chloro­methane. The solutions were combined; the mixture was then overlayered with *n*-pentane and stored in a refrigerator (276 K) for 7 d. However, no crystals formed. The solvents were then removed under vacuum. The solid residue was washed with *n*-pentane and recrystallized from di­chloro­methane/*n*-pentane. Yield 77 mg (0.127 mmol, 64%). However, the elemental analysis was poor and some colourless crystals of PhICl_2_ were identified by their cell constants. ^31^P-NMR (81 MHz, CDCl_3_, 300 K): δ [ppm] 90.18.

Compound **21a**: 236 mg (0.5 mmol) of ^*i*^Pr_3_PSeAuCl and 344 mg (1.25 mmol) of PhICl_2_ were dissolved in 10/9 mL of di­chloro­methane, respectively. The solutions were combined and stirred for 30 min, during which time the solution changed from red to yellow; the solvents were then removed under vacuum. The product proved to be very sensitive to air and moisture, and decomposed rapidly in solution. ^31^P-NMR (81 MHz, CDCl_3_, 300 K): δ [ppm] 92.56, ^1^ *J*_P–Se_ 416 Hz. Despite the failure to prepare the substance in qu­antity, single crystals were obtained by overlaying a solution in di­chloro­methane with *n*-pentane and storing it in a refrigerator (276 K) over the weekend.

Compounds **17b** and **21b**: Treatment of solutions of ^*i*^Pr_3_P*E*AuBr_3_ in di­chloro­methane with excess elemental bromine led to solutions whose ^31^P-NMR spectra showed the presence of both starting material and product (**17b** δ 84.15; **21b** δ 80.47). Removal of the solvent, followed by crystallization attempts, led to some single crystals of the products. For **21b**, the NMR spectrum was of poor quality and no P–Se coupling was detected.

Compounds **20b** and **24b**: Treatment of solutions of [(^*t*^Bu_3_P*E*)_2_Au][AuBr_4_] (the syntheses and structures of these are still to be published) in di­chloro­methane with excess elemental bromine led to solutions whose ^31^P-NMR spectra showed the presence of both starting material and product (**20b** δ 91.16; **24b** δ 88.08). Removal of the solvent, followed by crystallization attempts, led to some single crystals of **20b** (but not of **24b**). For **24b**, the NMR spectrum was of poor quality and no P–Se coupling was detected.

## 6. Refinement

Details of the measurements and refinements are given in Table 33 ▸. Structures were refined anisotropically on *F* ^2^. Methine hydrogens were included at calculated positions and refined using a riding model with C—H = 1.00 Å and *U*(H) = 1.2 × *U*_eq_(C). Methyl groups were refined, using the command ‘AFIX 137’, as idealized rigid groups allowed to rotate but not to tip, with C—H = 0.98 Å, H—C—H = 109.5° and *U*(H) = 1.5 × *U*_eq_(C). This procedure is less reliable for heavy-atom structures, so that any postulated hydrogen bonds involving methyl hydrogen atoms should be inter­preted with caution.

*Special features*: For **17a**, there is a large difference peak of *ca* 5.6 e A^−3^ near the gold atom Au1. This peak may probably be attributed to residual absorption errors. It can be ‘removed’ by assuming a disorder of the AuCl_4_ anion (the minor site then has an occupation of *ca* 3%), but we do not find this strategy satisfactory. [*Note added during revision.* The referee asked for an explanation of ‘unsatisfactory’: Heavy-atom structures generally show significant residual electron density near the heavy atom, and this can be large if the data are not of high quality. These peaks can always be removed by the *ad hoc* method of refining them as an alternative heavy-atom position with low occupation. However, the lighter atom positions (here chlorines with occupation factor 3%) cannot be clearly identified, but only guessed and then refined with strict restraints. We therefore prefer to assume that the large peak is a result of imperfect data, and that it would not be justifiable to remove it for cosmetic reasons.] The *U* values of this structure are significantly higher than those of the other structures, and the corresponding figure shows 30% rather than 50% ellipsoids. For **19a** (and the isotypic **23a**), the anion centred on Au1 is disordered, with the atoms Cl3 and Cl4 being slightly displaced from the twofold axis. Dimensions of disordered groups should be inter­preted with caution. For **20a**, the data were affected by a small twinning component (rotated by 180° about the *c* axis) that was at first not detected, but which led to poor agreement of data with *l* = 4. Data reduction and refinement (‘HKLF 5’ method; Sheldrick, 2015 ▸) as a non-merohedral twin led to a BASF parameter (relative volume of the smaller twin component) of 0.0300 (3). As is often the case with ‘HKLF 5’ refinements, several reflections were severely in error; 20 of these were removed from the dataset. Because equivalent reflections are merged during the generation of the ‘HKLF 5’ intensity dataset, and because both overlapped and non-overlapped reflections are included in the refinement, the number of reflections should be inter­preted carefully. The low GOOF value may be associated with the difficulty of estimating s.u.’s for the intensities of the small twin component (and of the weak intensities not corresponding to the pseudo-*I* centring caused by the presence of two gold atoms on special positions). Similarly, the crystal of **23b** was a two-component non-merohedral twin (by 180° rotation about the a* axis). The relative volume of the smaller twin component refined to 0.0807 (6). The *U* values of the carbon atoms were restrained to be less anisotropic using the command ‘ISOR $C 0.005’. For several of the non-twinned structures, a few (1–3) poorly fitting reflections (Δ/σ *ca* 7–11) were omitted from the refinement.

## Supplementary Material

Crystal structure: contains datablock(s) 17a, 18a, 19a, 20a, 21a, 22a, 23a, 17b, 18b, 19b, 20b, 21b, 22b, 23b, global. DOI: 10.1107/S2056989024002780/yz2053sup1.cif

Structure factors: contains datablock(s) 17a. DOI: 10.1107/S2056989024002780/yz205317asup2.hkl

Structure factors: contains datablock(s) 18a. DOI: 10.1107/S2056989024002780/yz205318asup3.hkl

Structure factors: contains datablock(s) 19a. DOI: 10.1107/S2056989024002780/yz205319asup4.hkl

Structure factors: contains datablock(s) 20a. DOI: 10.1107/S2056989024002780/yz205320asup5.hkl

Structure factors: contains datablock(s) 21a. DOI: 10.1107/S2056989024002780/yz205321asup6.hkl

Structure factors: contains datablock(s) 22a. DOI: 10.1107/S2056989024002780/yz205322asup7.hkl

Structure factors: contains datablock(s) 23a. DOI: 10.1107/S2056989024002780/yz205323asup8.hkl

Structure factors: contains datablock(s) 17b. DOI: 10.1107/S2056989024002780/yz205317bsup9.hkl

Structure factors: contains datablock(s) 18b. DOI: 10.1107/S2056989024002780/yz205318bsup10.hkl

Structure factors: contains datablock(s) 19b. DOI: 10.1107/S2056989024002780/yz205319bsup11.hkl

Structure factors: contains datablock(s) 20b. DOI: 10.1107/S2056989024002780/yz205320bsup12.hkl

Structure factors: contains datablock(s) 21b. DOI: 10.1107/S2056989024002780/yz205321bsup13.hkl

Structure factors: contains datablock(s) 22b. DOI: 10.1107/S2056989024002780/yz205322bsup14.hkl

Structure factors: contains datablock(s) 23b. DOI: 10.1107/S2056989024002780/yz205323bsup15.hkl

CCDC references: 2156871, 2345294, 2345295, 2156781, 2156391, 2345296, 2345297, 2156880, 2156882, 2156884, 2156886, 2156881, 2156883, 2156885

Additional supporting information:  crystallographic information; 3D view; checkCIF report

## Figures and Tables

**Figure 4:**
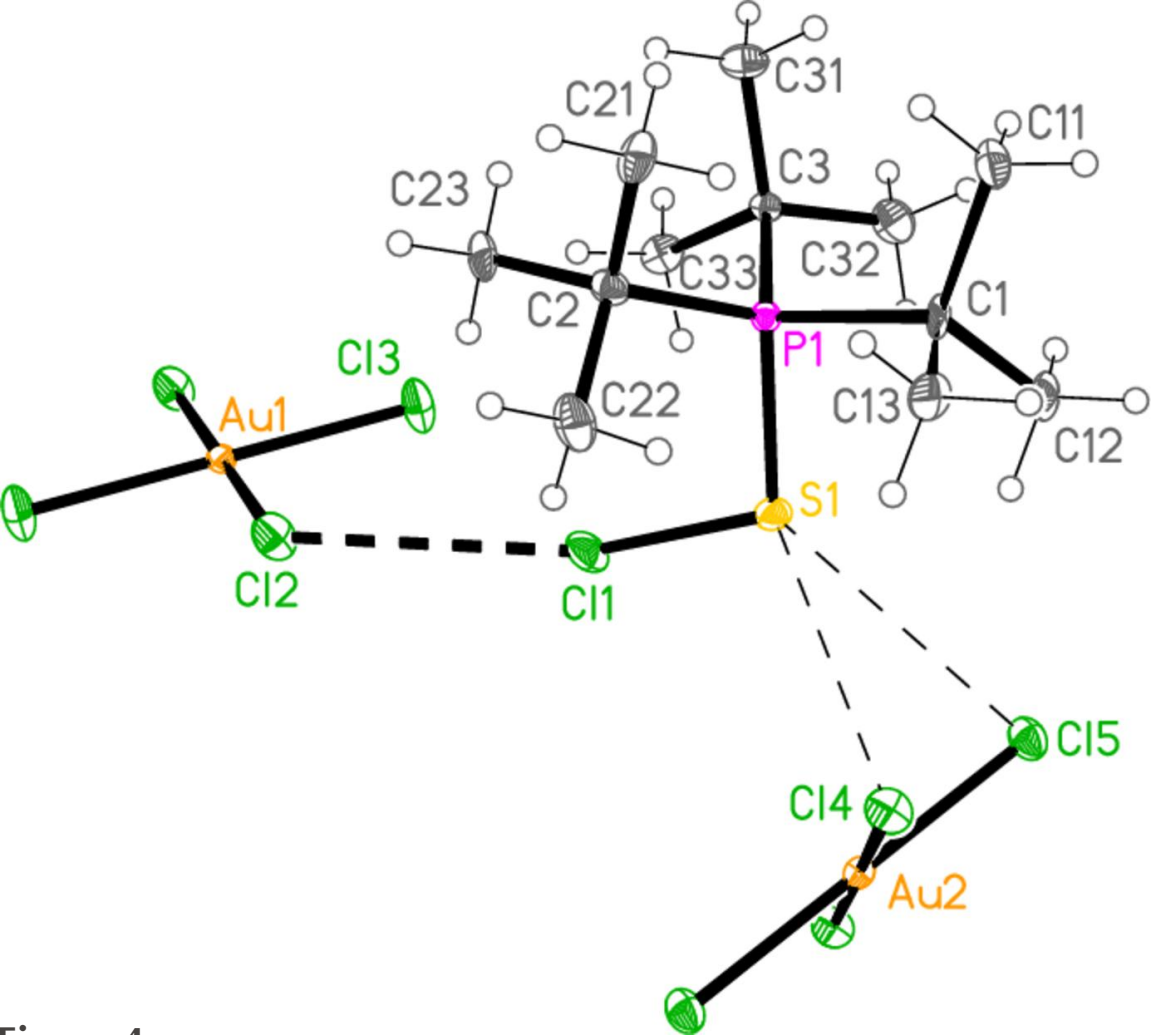
The structure of compound **20** ***a*** in the crystal. Ellipsoids represent 50% probability levels.

**Figure 5:**
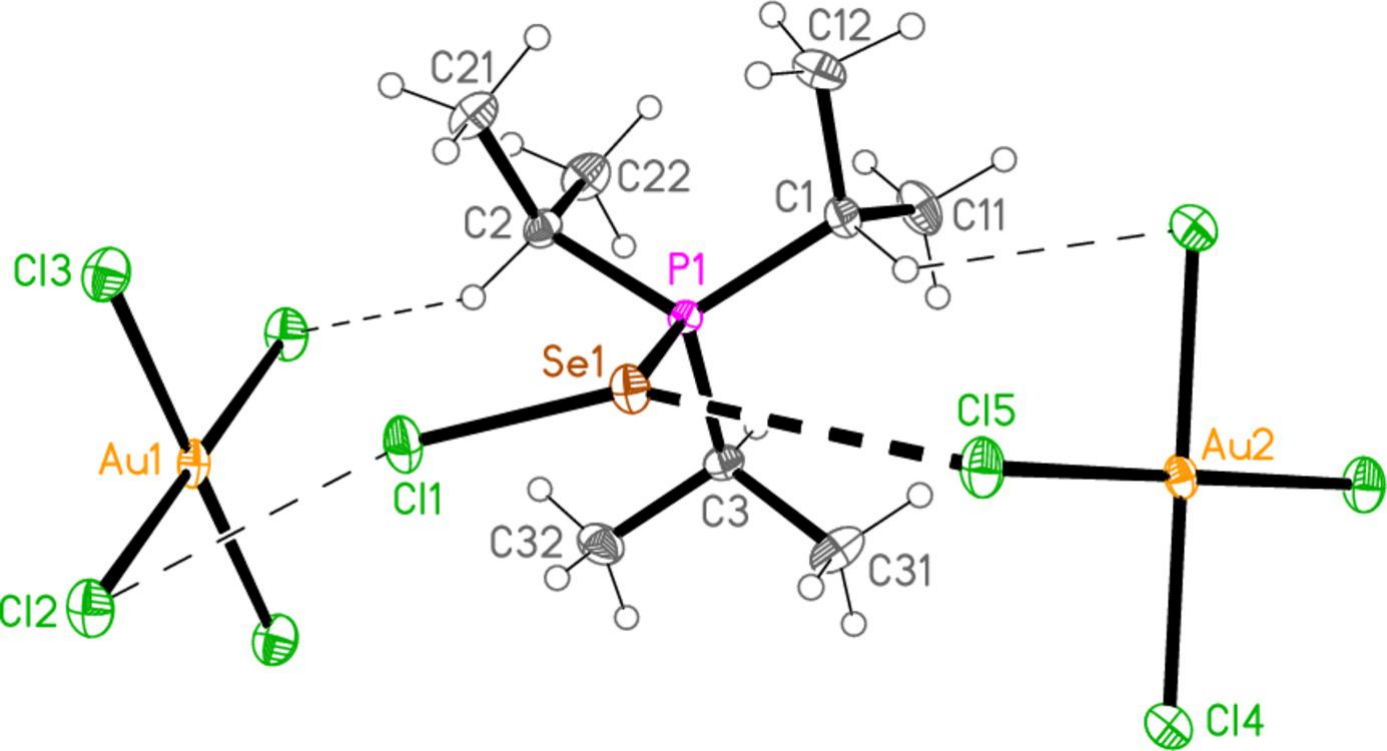
The structure of compound **21** ***a*** in the crystal. Ellipsoids represent 50% probability levels.

**Figure 6:**
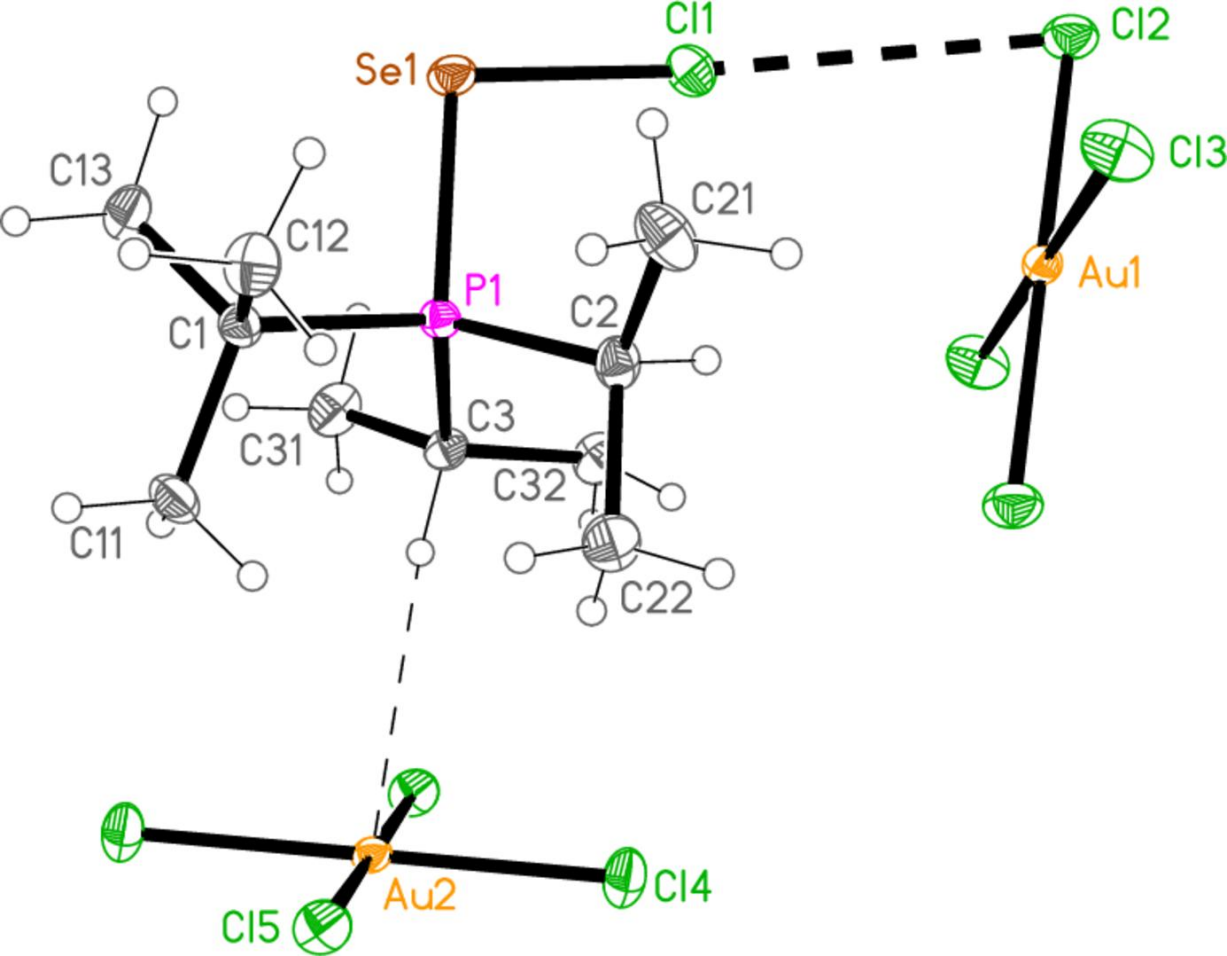
The structure of compound **22** ***a*** in the crystal. Ellipsoids represent 50% probability levels.

**Figure 7:**
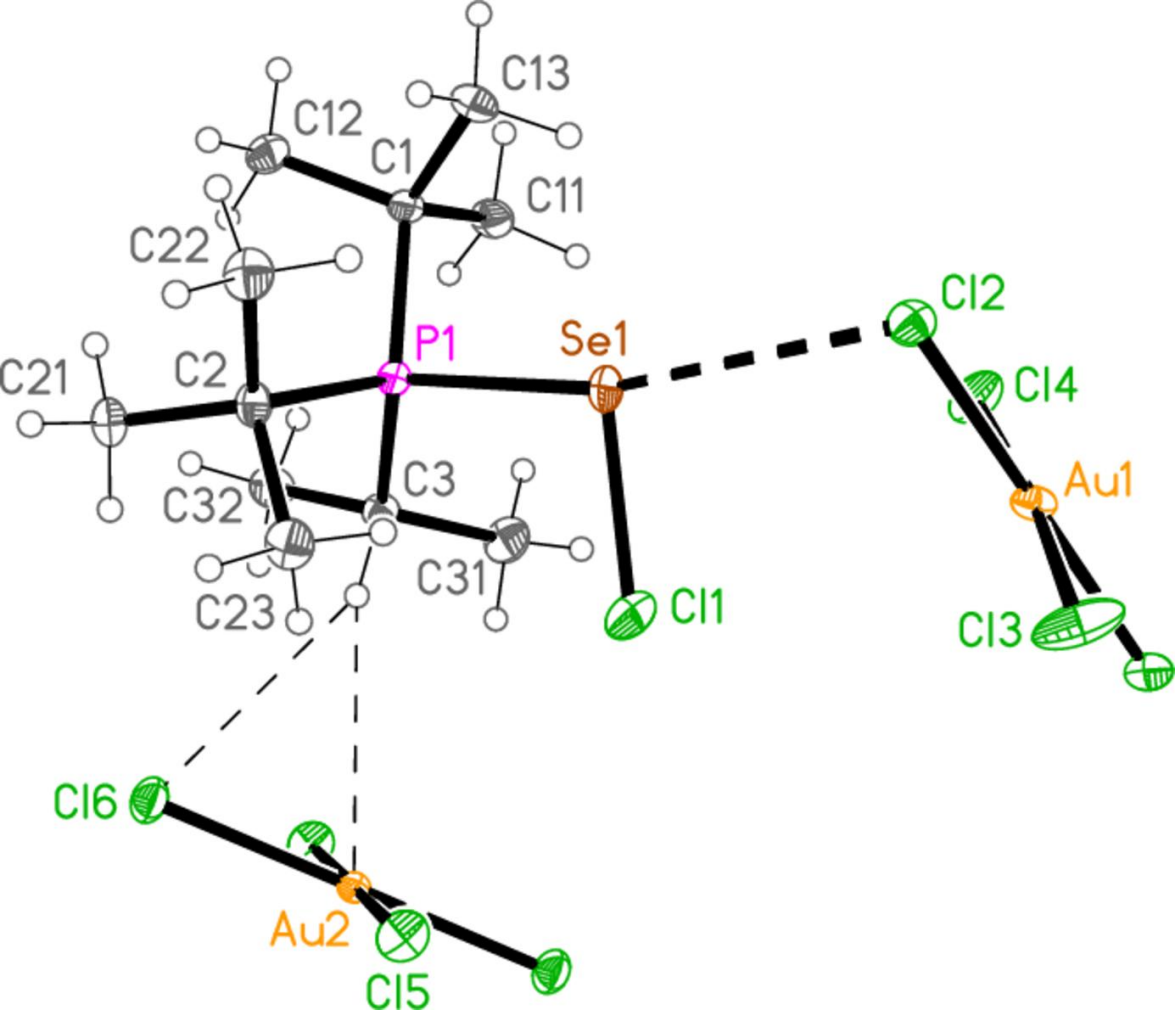
The structure of compound **23** ***a*** in the crystal. Ellipsoids represent 50% probability levels. Only one position of the disordered atoms Cl3 and Cl4 is shown.

**Figure 8:**
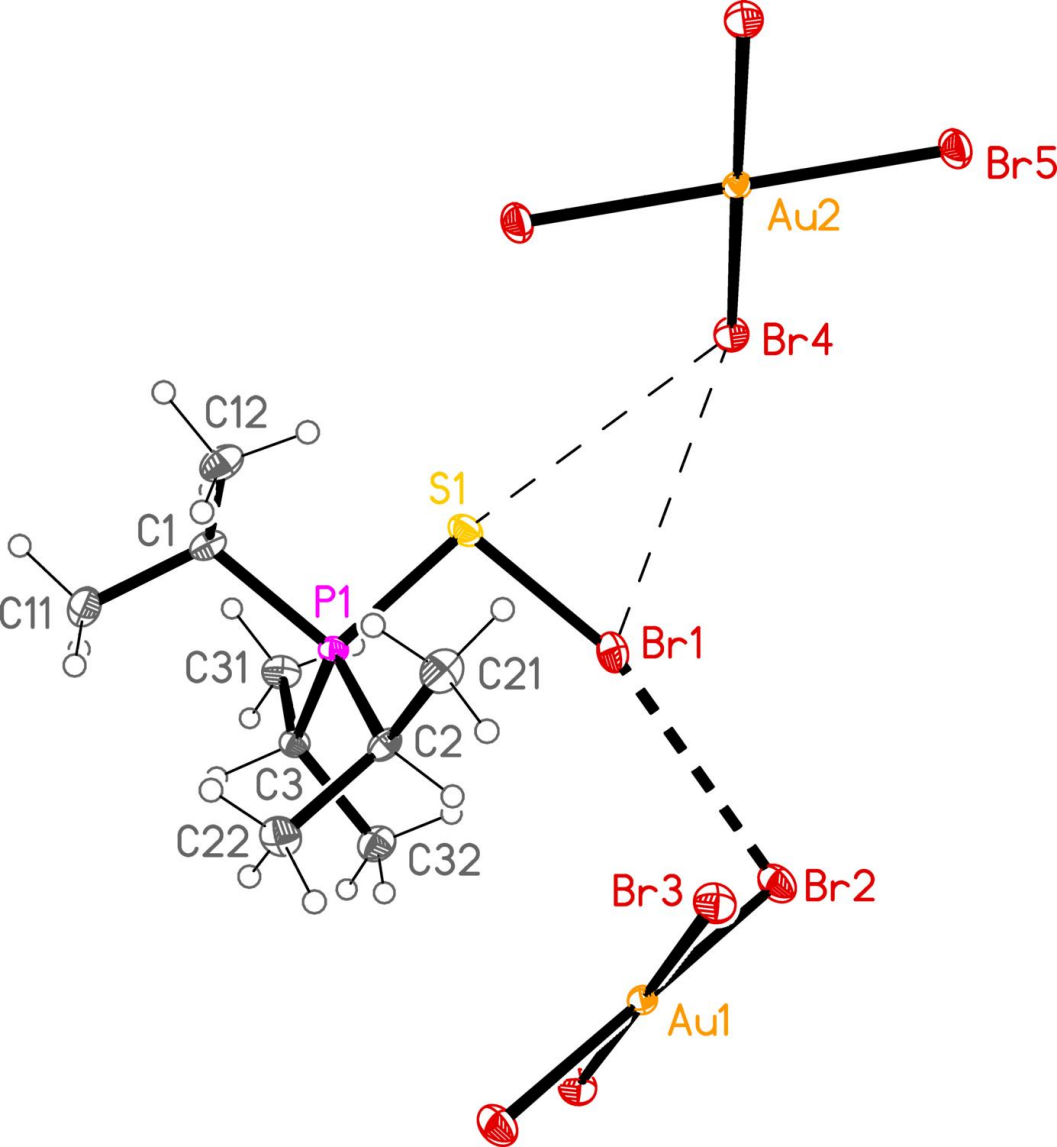
The structure of compound **17** ***b*** in the crystal. Ellipsoids represent 50% probability levels.

**Figure 9:**
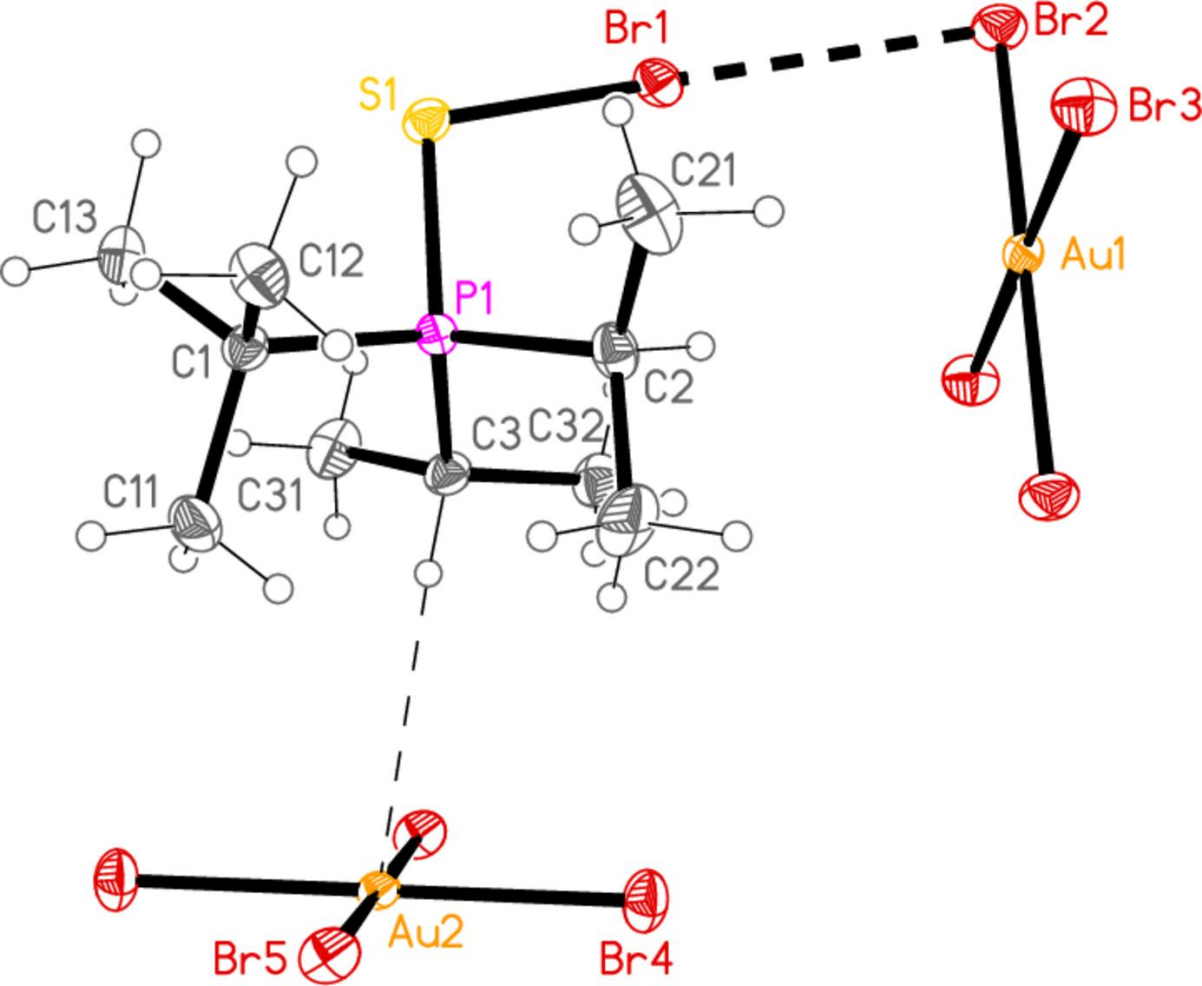
The structure of compound **18** ***b*** in the crystal. Ellipsoids represent 50% probability levels.

**Figure 11:**
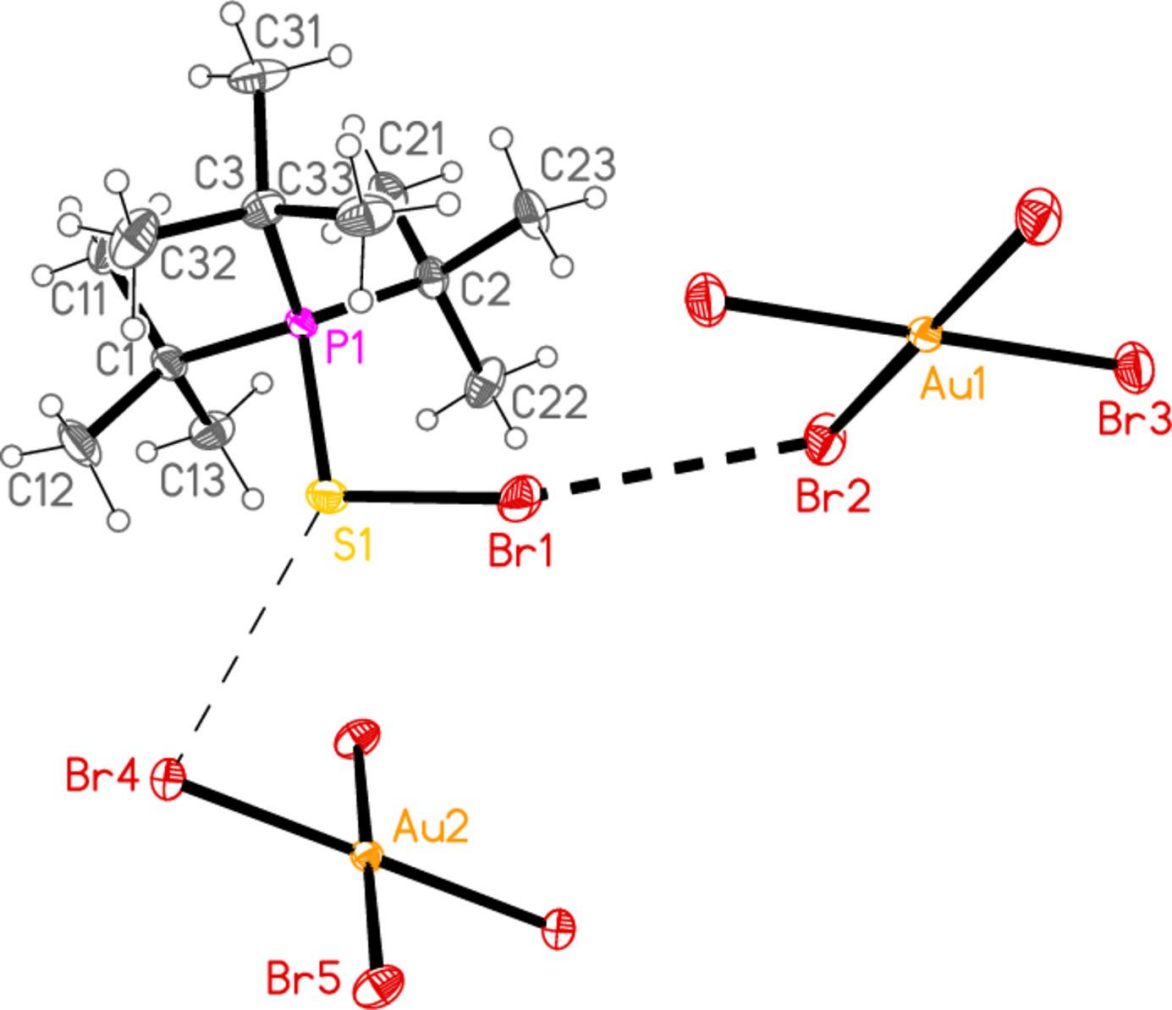
The structure of compound **20** ***b*** in the crystal. Ellipsoids represent 50% probability levels.

**Figure 12:**
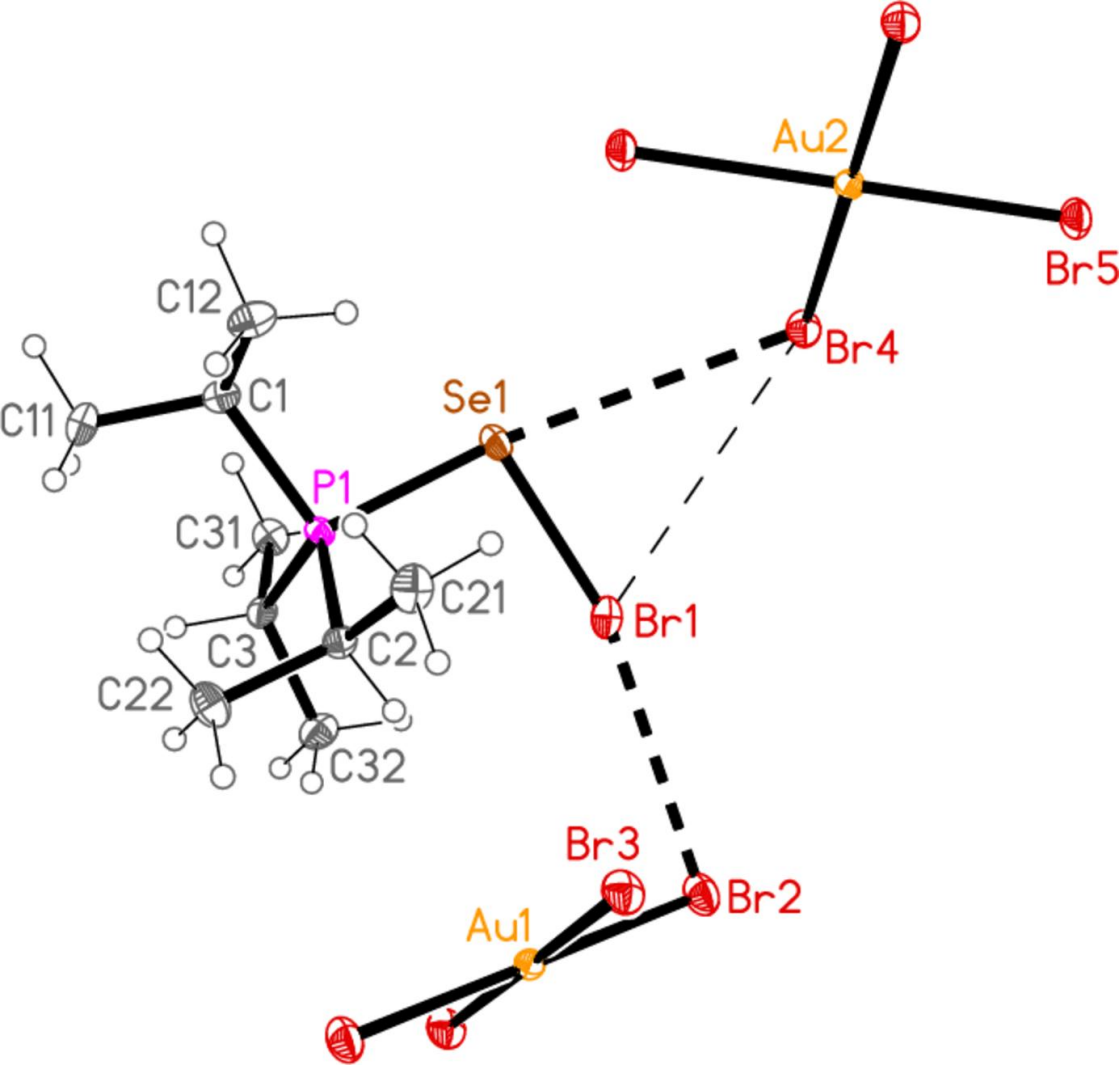
The structure of compound **21** ***b*** in the crystal. Ellipsoids represent 50% probability levels.

**Figure 13:**
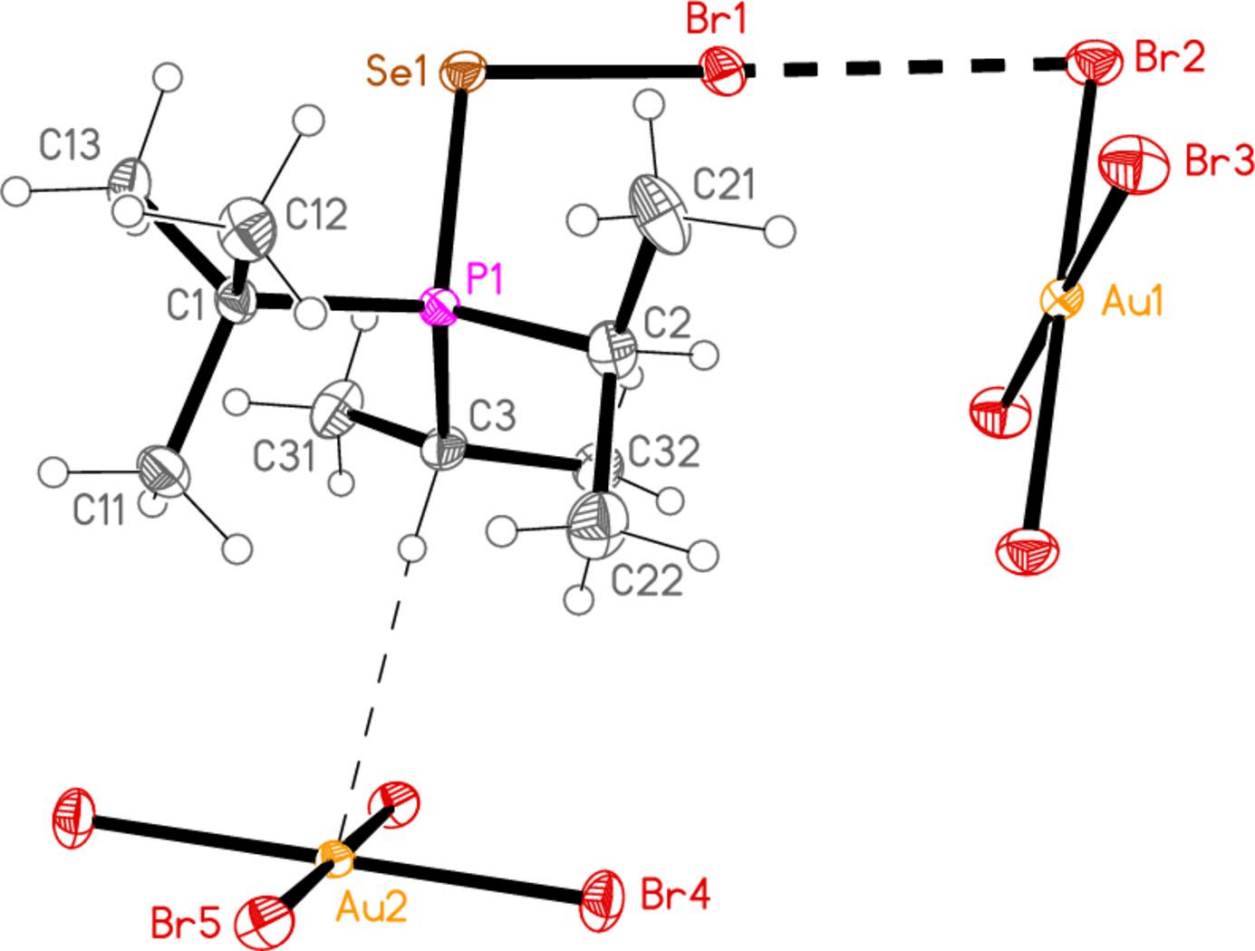
The structure of compound **22** ***b*** in the crystal. Ellipsoids represent 50% probability levels.

**Figure 14:**
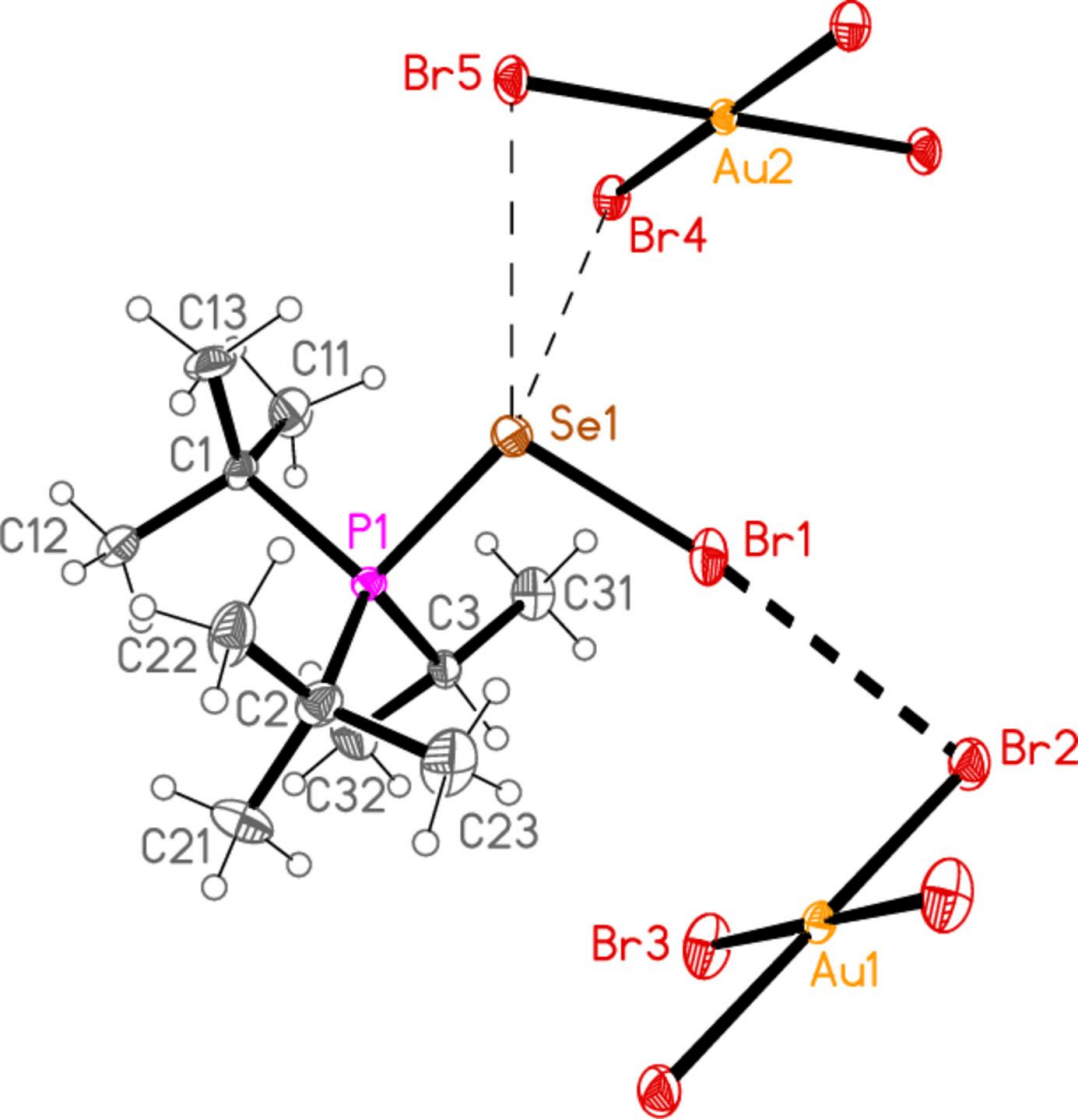
The structure of compound **23** ***c*** in the crystal. Ellipsoids represent 50% probability levels.

**Table 1:** Compositions of the [*R* ^1^ *R* ^2^ *R* ^3^P*EX*][Au*X*_4_] structures presented in this paper (see Scheme)

Compound	*R* ^1^	*R* ^2^	*R* ^3^	*E*	*X*
**17*a***	^*i*^Pr	^*i*^Pr	^*i*^Pr	S	Cl
**18*a***	^*i*^Pr	^*i*^Pr	^*t*^Bu	S	Cl
**19*a***	^*i*^Pr	^*t*^Bu	^*t*^Bu	S	Cl
**20*a***	^*t*^Bu	^*t*^Bu	^*t*^Bu	S	Cl
**21*a***	^*i*^Pr	^*i*^Pr	^*i*^Pr	Se	Cl
**22*a***	^*i*^Pr	^*i*^Pr	^*t*^Bu	Se	Cl
**23*a***	^*i*^Pr	^*t*^Bu	^*t*^Bu	Se	Cl
**17*b***	^*i*^Pr	^*i*^Pr	^*i*^Pr	S	Br
**18*b***	^*i*^Pr	^*i*^Pr	^*t*^Bu	S	Br
**19*b***	^*i*^Pr	^*t*^Bu	^*t*^Bu	S	Br
**20*b***	^*t*^Bu	^*t*^Bu	^*t*^Bu	S	Br
**21*b***	^*i*^Pr	^*i*^Pr	^*i*^Pr	Se	Br
**22*b***	^*i*^Pr	^*i*^Pr	^*t*^Bu	Se	Br
**23*b***	^*i*^Pr	^*t*^Bu	^*t*^Bu	Se	Br

**Table 2:** Selected geometric parameters (Å, °) for **17a** [Chem scheme1]

P1—S1	2.091 (2)	Au1—Cl3	2.2789 (16)
S1—Cl1	2.023 (3)	Au1—Cl5	2.2797 (16)
Au1—Cl2	2.2745 (17)	Au1—Cl4	2.2884 (17)
			
Cl1—S1—P1	99.25 (11)		
			
C1—P1—S1—Cl1	164.4 (2)		

**Table 3:** Selected geometric parameters (Å, °) for **18a** [Chem scheme1]

P1—S1	2.0970 (7)	Au1—Cl2	2.2847 (5)
S1—Cl1	2.0316 (7)	Au2—Cl4	2.2753 (5)
Au1—Cl3	2.2744 (5)	Au2—Cl5	2.2801 (5)
			
Cl1—S1—P1	101.57 (3)		
			
C1—P1—S1—Cl1	164.48 (7)		

**Table 4:** Selected geometric parameters (Å, °) for **19a** [Chem scheme1]

P1—S1	2.1035 (16)	Au1—Cl3	2.274 (2)
S1—Cl1	2.0310 (19)	Au2—Cl6	2.2738 (11)
Au1—Cl4	2.269 (2)	Au2—Cl5	2.2796 (12)
			
Cl1—S1—P1	103.06 (7)		
			
C1—P1—S1—Cl1	−160.17 (16)		

**Table 5:** Selected geometric parameters (Å, °) for **20a** [Chem scheme1]

P1—S1	2.0983 (13)	Au1—Cl2	2.2850 (10)
S1—Cl1	2.0307 (13)	Au2—Cl5	2.2821 (11)
Au1—Cl3	2.2818 (10)	Au2—Cl4	2.2860 (11)
			
Cl1—S1—P1	104.74 (5)		
			
C1—P1—S1—Cl1	163.38 (16)		

**Table 6:** Selected geometric parameters (Å, °) for **21a** [Chem scheme1]

P1—Se1	2.2488 (5)	Au1—Cl3	2.2838 (5)
Se1—Cl1	2.1736 (5)	Au2—Cl4	2.2795 (5)
Au1—Cl2	2.2778 (5)	Au2—Cl5	2.2836 (5)
			
Cl1—Se1—P1	98.381 (19)		
			
C1—P1—Se1—Cl1	161.60 (7)		

**Table 7:** Selected geometric parameters (Å, °) for **22a** [Chem scheme1]

P1—Se1	2.2467 (7)	Au1—Cl2	2.2842 (7)
Se1—Cl1	2.1654 (7)	Au2—Cl4	2.2781 (7)
Au1—Cl3	2.2736 (7)	Au2—Cl5	2.2823 (6)
			
Cl1—Se1—P1	98.35 (3)		
			
C1—P1—Se1—Cl1	163.52 (9)		

**Table 8:** Selected geometric parameters (Å, °) for **23a** [Chem scheme1]

P1—Se1	2.2557 (6)	Au1—Cl2	2.2833 (6)
Se1—Cl1	2.1645 (7)	Au2—Cl6	2.2735 (6)
Au1—Cl4	2.2681 (13)	Au2—Cl5	2.2784 (6)
Au1—Cl3	2.2765 (12)		
			
Cl1—Se1—P1	100.40 (3)		
			
C1—P1—Se1—Cl1	−159.50 (8)		

**Table 9:** Selected geometric parameters (Å, °) for **17b** [Chem scheme1]

P1—S1	2.0852 (10)	Au1—Br3	2.4308 (3)
S1—Br1	2.1977 (8)	Au2—Br4	2.4259 (3)
Au1—Br2	2.4201 (3)	Au2—Br5	2.4265 (3)
			
P1—S1—Br1	99.46 (4)		
			
C1—P1—S1—Br1	174.93 (9)		

**Table 10:** Selected geometric parameters (Å, °) for **18b** [Chem scheme1]

P1—S1	2.0902 (12)	Au1—Br2	2.4287 (4)
S1—Br1	2.2028 (10)	Au2—Br4	2.4154 (4)
Au1—Br3	2.4180 (4)	Au2—Br5	2.4199 (4)
			
P1—S1—Br1	102.51 (5)		
			
C1—P1—S1—Br1	162.05 (13)		

**Table 11:** Selected geometric parameters (Å, °) for **19b** [Chem scheme1]

P1—S1	2.0992 (16)	Au1—Br2	2.4247 (5)
S1—Br1	2.2077 (12)	Au2—Br5	2.4207 (4)
Au1—Br3	2.4179 (5)	Au2—Br4	2.4228 (4)
			
P1—S1—Br1	104.48 (6)		
			
C1—P1—S1—Br1	−160.33 (16)		

**Table 12:** Selected geometric parameters (Å, °) for **20b** [Chem scheme1]

S1—P1	2.0973 (16)	Au1—Br3	2.4257 (5)
Br1—S1	2.1934 (13)	Au2—Br4	2.4171 (5)
Au1—Br2	2.4178 (5)	Au2—Br5	2.4234 (5)
			
P1—S1—Br1	105.70 (6)		
			
Br1—S1—P1—C1	161.63 (16)		

**Table 13:** Selected geometric parameters (Å, °) for **21b** [Chem scheme1]

P1—Se1	2.2364 (9)	Au1—Br3	2.4264 (4)
Se1—Br1	2.3179 (5)	Au2—Br4	2.4230 (4)
Au1—Br2	2.4162 (4)	Au2—Br5	2.4258 (3)
			
P1—Se1—Br1	96.32 (3)		
			
C1—P1—Se1—Br1	174.59 (11)		

**Table 14:** Selected geometric parameters (Å, °) for **22b** [Chem scheme1]

P1—Se1	2.2453 (10)	Au1—Br2	2.4287 (4)
Se1—Br1	2.3237 (5)	Au2—Br4	2.4178 (4)
Au1—Br3	2.4162 (4)	Au2—Br5	2.4202 (4)
			
P1—Se1—Br1	99.52 (3)		
			
C1—P1—Se1—Br1	161.21 (13)		

**Table 15:** Selected geometric parameters (Å, °) for **23b** [Chem scheme1]

P1—Se1	2.2534 (19)	Au1—Br2	2.4249 (8)
Se1—Br1	2.3255 (10)	Au2—Br5	2.4201 (7)
Au1—Br3	2.4142 (8)	Au2—Br4	2.4221 (7)
			
P1—Se1—Br1	101.91 (6)		
			
C1—P1—Se1—Br1	−160.4 (2)		

**Table 16:** Selected dimensions (Å, °) of published structures of halochalcogenyl­phospho­nium cations

Compound	P—*E*	*E*—*X*	P—*E*—*X*	Reference
[Ph_3_PSCl][AuCl_4_]	2.0912 (12)	2.2278 (13)	99.87 (5)	Taouss *et al.* (2015 ▸)
[Ph_3_PSBr][AuBr_4_]	2.0854 (16)	2.2023 (14)	101.31 (6)	Taouss *et al.* (2015 ▸)
[Ph_3_PSeBr][AuBr_4_]	2.2250 (7)	2.3121 (4)	97.10 (2)	Taouss *et al.* (2015 ▸)
[Ph_3_PSeCl]_2_[Au_4_Se_2_Cl_10_]	2.216 (2)	2.222 (3)	96.83 (9)	Taouss *et al.* (2015 ▸)
[(PCP)^*i*^Pr_2_PSeCl][AuCl_4_]^*a*^	2.257 (2)	2.207 (2)	94.69 (9)	Upmann *et al.* (2019 ▸)

Note: (*a*) PCP = [2.2]para­cyclo­phanyl.

**Table 17:** Dimensions (Å, °) of halogen⋯halogen contacts between cations and anions The halogen atoms are numbered such that the contact is always *X*1⋯*X*2. For references, see Table 16 ▸.

Compound	*X*⋯*X*	*E*—*X*⋯*X*	*X*⋯*X*—Au	*X*⋯*X*—Au—*X*_*cis*_^*b*^
**18*a*** (*X* = Cl)	3.3964 (8)	171.74 (3)	75.21 (2)	86.61 (2)
**20*a*** (*X* = Cl)	3.2652 (14)	159.83 (6)	115.19 (4)	8.88 (5)
**21*a*** (*X* = Cl)	3.6071 (5)	164.28 (2)	72.72 (2)	74.65 (2)
**22*a*** (*X* = Cl)	3.4465 (10)	171.45 (3)	73.52 (2)	85.07 (3)
**17*b*** (*X* = Br)	3.3206 (5)	165.22 (2)	86.52 (1)	69.07 (1)
**18*b*** (*X* = Br)	3.2874 (6)	174.89 (3)	78.06 (1)	89.71 (1)
**19*b*** (*X* = Br)	3.2696 (7)	173.07 (4)	84.19 (2)	76.31 (2)
**20*b*** (*X* = Br)	3.3465 (7)	157.33 (3)	112.62 (2)	9.80 (2)
**21*b*** (*X* = Br)	3.3445 (6)	166.59 (2)	85.49 (1)	69.34 (1)
**22*b*** (*X* = Br)	3.3687 (6)	175.31 (2)	75.54 (1)	88.46 (1)
**23*b*** (*X* = Br)	3.3416 (11)	172.85 (4)	82.51 (3)	77.96 (3)
[Ph_3_PSCl][AuCl_4_]	3.2489 (13)	169.81 (5)	105.99 (4)	79.74 (4)
[(PCP)^*i*^Pr_2_PSeCl][AuCl_4_]^*a*^	3.696 (3)	162.71 (11)	72.40 (4)	87.76 (9)
[Ph_3_PSBr][AuBr_4_]	3.1509 (7)	174.79 (4)	99.41 (2)	80.04 (2)
[Ph_3_PSeBr][AuBr_4_]	3.4009 (5)	160.89 (1)	98.79 (1)	48.32 (1)

Notes: (*a*) PCP = [2.2]para­cyclo­phanyl; (*b*) the smaller absolute torsion angle (of two) is shown; the value to the other *X*_*cis*_ is the complementary angle (exactly or approximately, depending on the symmetry) with the opposite sign.

**Table 18:** Dimensions (Å, °) of chalcogen⋯chlorine contacts between cations and anions

Compound	*E*⋯Cl	P—*E*⋯Cl	*E*⋯Cl—Au	*E*⋯Cl—Au—Cl_*cis*_^*b*^
**17*a*** (*E* = S)	S1⋯Cl5 3.553 (3)	152.59 (11)	109.61 (7)	26.54 (8)
**19*a*** (*E* = S)^*a*^	S1⋯Cl2 3.3240 (17)	162.69 (7)	95.10 (4)	61.7 (2)
**23*a*** (*E* = Se)^*a*^	Se1⋯Cl2 3.3052 (6)	164.50 (2)	95.40 (6)	59.37 (2)
[Ph_3_PSeCl]_2_[Au_4_Se_2_Cl_10_]^*c*^	Se1⋯Cl6 3.308 (3)	158.99 (9)	116.86 (9)	55.06 (10)

Notes: (*a*) Compounds **19*a*** and **23*a*** are isotypic. In both structures, the *cis* chlorines are disordered. (*b*) The smaller absolute torsion angle (of two) is shown; the value to the other Cl_*cis*_ is the complementary angle (exactly or approximately, depending on the symmetry) with the opposite sign. (*c*) Upmann *et al.* (2019 ▸).

**Table 19:** Hydrogen-bond geometry (Å, °) for **17a** [Chem scheme1]

*D*—H⋯*A*	*D*—H	H⋯*A*	*D*⋯*A*	*D*—H⋯*A*
C2—H2⋯Cl1	1.00	2.86	3.343 (7)	110
C32—H32*C*⋯Cl1	0.98	2.83	3.527 (7)	129
C12—H12*B*⋯S1	0.98	2.57	3.069 (9)	111
C21—H21*B*⋯S1	0.98	2.88	3.415 (8)	115
C22—H22*C*⋯Cl3^i^	0.98	2.89	3.843 (7)	164
C1—H1⋯Cl4^i^	1.00	2.83	3.721 (7)	149
C32—H32*A*⋯Cl4^ii^	0.98	2.88	3.731 (7)	145
C3—H3⋯Cl5^ii^	1.00	2.72	3.701 (7)	167

Symmetry codes: (i) 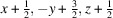 ; (ii) 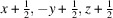 .

**Table 20:** Hydrogen-bond geometry (Å, °) for **18a** [Chem scheme1]

*D*—H⋯*A*	*D*—H	H⋯*A*	*D*⋯*A*	*D*—H⋯*A*
C13—H13*B*⋯S1	0.98	2.63	3.073 (2)	107
C2—H2⋯Cl1	1.00	2.95	3.383 (2)	107
C32—H32*C*⋯Cl1	0.98	2.90	3.635 (2)	133
C21—H21*C*⋯Cl3	0.98	2.93	3.705 (2)	137
C11—H11*A*⋯Cl5	0.98	2.97	3.742 (2)	136
C3—H3⋯Au2	1.00	2.75	3.693 (2)	158
C2—H2⋯Cl2^i^	1.00	2.93	3.688 (2)	133
C22—H22*A*⋯Cl3^ii^	0.98	2.82	3.642 (2)	142
C12—H12*B*⋯Cl3^iii^	0.98	2.91	3.754 (2)	145
C13—H13*B*⋯Cl3^iii^	0.98	2.98	3.876 (2)	153
C12—H12*A*⋯Cl4^iv^	0.98	2.78	3.699 (2)	157
C13—H13*A*⋯Cl4^v^	0.98	2.93	3.760 (2)	143

Symmetry codes: (i) 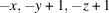 ; (ii) 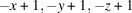 ; (iii) 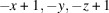 ; (iv) 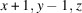 ; (v) 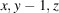 .

**Table 21:** Hydrogen-bond geometry (Å, °) for **19a** [Chem scheme1]

*D*—H⋯*A*	*D*—H	H⋯*A*	*D*⋯*A*	*D*—H⋯*A*
C3—H3⋯Au2	1.00	2.93	3.786 (5)	144
C3—H3⋯Cl6	1.00	2.90	3.751 (5)	143
C13—H13*A*⋯S1	0.98	2.46	3.049 (5)	118
C23—H23*B*⋯Cl1	0.98	2.74	3.305 (5)	117
C31—H31*B*⋯Cl1	0.98	2.66	3.393 (5)	132
C23—H23*A*⋯Cl6^i^	0.98	2.74	3.713 (5)	173
C21—H21*C*⋯Cl6^ii^	0.98	2.90	3.672 (5)	137
C13—H13*B*⋯Cl3^iii^	0.98	2.81	3.536 (8)	131
C22—H22*B*⋯Cl2^iv^	0.98	2.87	3.783 (5)	156
C13—H13*C*⋯Cl4^v^	0.98	2.89	3.677 (19)	138

Symmetry codes: (i) 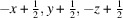 ; (ii) 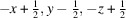 ; (iii) 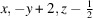 ; (iv) 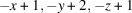 ; (v) 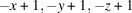 .

**Table 22:** Hydrogen-bond geometry (Å, °) for **20a** [Chem scheme1]

*D*—H⋯*A*	*D*—H	H⋯*A*	*D*⋯*A*	*D*—H⋯*A*
C23—H23*B*⋯Cl1	0.98	2.71	3.522 (4)	141
C22—H22*B*⋯Cl1	0.98	2.68	3.497 (4)	141
C33—H33*B*⋯Cl1	0.98	2.82	3.401 (4)	118
C13—H13*A*⋯Cl5^i^	0.98	2.88	3.796 (4)	156
C12—H12*C*⋯Cl5	0.98	2.92	3.810 (4)	151
C31—H31*C*⋯Cl5^ii^	0.98	2.84	3.763 (4)	158
C12—H12*C*⋯S1	0.98	2.44	3.027 (4)	118
C22—H22*B*⋯S1	0.98	2.92	3.370 (4)	109
C33—H33*B*⋯S1	0.98	2.92	3.424 (4)	113
C21—H21*A*⋯Cl4^iii^	0.98	2.77	3.708 (4)	161

Symmetry codes: (i) 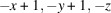 ; (ii) 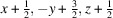 ; (iii) 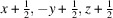 .

**Table 23:** Hydrogen-bond geometry (Å, °) for **21a** [Chem scheme1]

*D*—H⋯*A*	*D*—H	H⋯*A*	*D*⋯*A*	*D*—H⋯*A*
C21—H21*B*⋯Cl1	0.98	2.84	3.545 (2)	129
C32—H32*C*⋯Cl1	0.98	2.95	3.712 (2)	135
C1—H1⋯Cl5	1.00	2.93	3.8615 (19)	156
C2—H2⋯Cl2^i^	1.00	2.82	3.6147 (19)	137
C3—H3⋯Cl2^ii^	1.00	2.94	3.5249 (19)	118
C31—H31*C*⋯Cl2^ii^	0.98	2.98	3.626 (2)	125
C21—H21*C*⋯Cl2^iii^	0.98	2.98	3.868 (2)	151
C11—H11*C*⋯Cl4^iii^	0.98	2.87	3.828 (2)	166
C1—H1⋯Cl4^iv^	1.00	2.78	3.5341 (19)	133

Symmetry codes: (i) 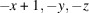 ; (ii) 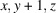 ; (iii) 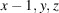 ; (iv) 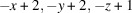 .

**Table 24:** Hydrogen-bond geometry (Å, °) for **22a** [Chem scheme1]

*D*—H⋯*A*	*D*—H	H⋯*A*	*D*⋯*A*	*D*—H⋯*A*
C13—H13*B*⋯Se1	0.98	2.65	3.142 (3)	111
C2—H2⋯Cl1	1.00	2.99	3.454 (3)	110
C3—H3⋯Au2	1.00	2.75	3.690 (3)	157
C32—H32*C*⋯Cl1	0.98	2.92	3.682 (3)	135
C21—H21*C*⋯Cl3	0.98	2.99	3.774 (3)	138
C11—H11*A*⋯Cl5	0.98	2.97	3.741 (3)	137
C2—H2⋯Cl2^i^	1.00	2.87	3.629 (3)	133
C22—H22*A*⋯Cl3^ii^	0.98	2.79	3.629 (3)	144
C12—H12*B*⋯Cl3^iii^	0.98	2.95	3.783 (3)	143
C13—H13*B*⋯Cl3^iii^	0.98	3.01	3.902 (3)	152
C12—H12*A*⋯Cl4^iv^	0.98	2.80	3.721 (3)	156
C13—H13*A*⋯Cl4^v^	0.98	2.94	3.751 (3)	141

Symmetry codes: (i) 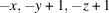 ; (ii) 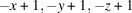 ; (iii) 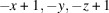 ; (iv) 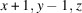 ; (v) 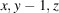 .

**Table 25:** Hydrogen-bond geometry (Å, °) for **23a** [Chem scheme1]

*D*—H⋯*A*	*D*—H	H⋯*A*	*D*⋯*A*	*D*—H⋯*A*
C3—H3⋯Au2	1.00	2.95	3.805 (2)	144
C3—H3⋯Cl6	1.00	2.88	3.730 (2)	143
C13—H13*A*⋯Se1	0.98	2.52	3.110 (3)	119
C23—H23*B*⋯Cl1	0.98	2.81	3.385 (3)	118
C31—H31*B*⋯Cl1	0.98	2.66	3.451 (3)	138
C23—H23*A*⋯Cl6^i^	0.98	2.76	3.736 (2)	173
C21—H21*C*⋯Cl6^ii^	0.98	2.90	3.674 (2)	137
C13—H13*B*⋯Cl3^iii^	0.98	2.80	3.498 (4)	129
C22—H22*B*⋯Cl2^iv^	0.98	2.86	3.787 (3)	157
C13—H13*C*⋯Cl4^v^	0.98	2.85	3.685 (15)	143

Symmetry codes: (i) 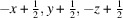 ; (ii) 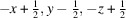 ; (iii) 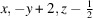 ; (iv) 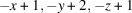 ; (v) 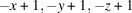 .

**Table 26:** Hydrogen-bond geometry (Å, °) for **17b** [Chem scheme1]

*D*—H⋯*A*	*D*—H	H⋯*A*	*D*⋯*A*	*D*—H⋯*A*
C21—H21*B*⋯Br1	0.98	3.28	3.864 (3)	120
C3—H3⋯Br2^i^	1.00	3.29	3.792 (3)	113
C31—H31*C*⋯Br2^i^	0.98	3.00	3.787 (3)	138
C32—H32*A*⋯Br2^i^	0.98	2.98	3.763 (3)	137
C1—H1⋯Br4^i^	1.00	3.15	3.997 (3)	144
C21—H21*C*⋯Br4^ii^	0.98	3.06	3.943 (3)	151
C31—H31*B*⋯Br4^i^	0.98	3.04	3.983 (3)	162
C1—H1⋯Br5^i^	1.00	2.98	3.802 (3)	140
C22—H22*C*⋯Br5^iii^	0.98	3.11	3.911 (3)	140

Symmetry codes: (i) 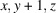 ; (ii) 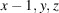 ; (iii) 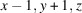 .

**Table 27:** Hydrogen-bond geometry (Å, °) for **18b** [Chem scheme1]

*D*—H⋯*A*	*D*—H	H⋯*A*	*D*⋯*A*	*D*—H⋯*A*
C13—H13*B*⋯S1	0.98	2.66	3.095 (4)	107
C2—H2⋯Br1	1.00	2.99	3.459 (4)	110
C3—H3⋯Au2	1.00	2.89	3.828 (4)	156
C32—H32*C*⋯Br1	0.98	3.01	3.739 (4)	132
C21—H21*C*⋯Br3	0.98	2.98	3.814 (4)	143
C11—H11*A*⋯Br5	0.98	3.07	3.778 (4)	130
C2—H2⋯Br2^i^	1.00	3.28	4.017 (4)	132
C22—H22*A*⋯Br3^ii^	0.98	2.95	3.771 (4)	142
C12—H12*B*⋯Br3^iii^	0.98	2.93	3.832 (4)	154
C13—H13*B*⋯Br3^iii^	0.98	3.18	4.075 (4)	152
C12—H12*A*⋯Br4^iv^	0.98	2.80	3.747 (4)	163
C13—H13*A*⋯Br4^v^	0.98	3.07	3.776 (4)	130

Symmetry codes: (i) 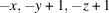 ; (ii) 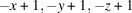 ; (iii) 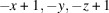 ; (iv) 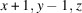 ; (v) 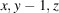 .

**Table 28:** Hydrogen-bond geometry (Å, °) for **19b** [Chem scheme1]

*D*—H⋯*A*	*D*—H	H⋯*A*	*D*⋯*A*	*D*—H⋯*A*
C13—H13*A*⋯S1	0.98	2.59	3.089 (5)	112
C31—H31*B*⋯Br1	0.98	2.72	3.487 (5)	135
C23—H23*B*⋯Br1	0.98	2.91	3.407 (6)	112
C3—H3⋯Br3	1.00	3.04	4.007 (5)	164
C23—H23*C*⋯Br3	0.98	3.09	4.040 (5)	165
C11—H11*C*⋯Br4	0.98	3.11	4.084 (5)	171
C31—H31*C*⋯Br2^i^	0.98	3.06	3.781 (5)	132
C23—H23*A*⋯Br4^ii^	0.98	2.90	3.824 (5)	157
C13—H13*C*⋯Br4^iii^	0.98	3.08	4.024 (5)	162
C32—H32*C*⋯Br4^i^	0.98	3.03	3.814 (5)	138
C11—H11*A*⋯Br5^iii^	0.98	3.06	3.846 (5)	138
C32—H32*C*⋯Br5^iv^	0.98	3.00	3.864 (5)	148

Symmetry codes: (i) 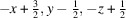 ; (ii) 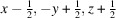 ; (iii) 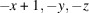 ; (iv) 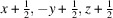 .

**Table 29:** Hydrogen-bond geometry (Å, °) for **20b** [Chem scheme1]

*D*—H⋯*A*	*D*—H	H⋯*A*	*D*⋯*A*	*D*—H⋯*A*
C12—H12*C*⋯S1	0.98	2.53	3.036 (5)	112
C22—H22*B*⋯Br1	0.98	2.74	3.569 (5)	143
C23—H23*A*⋯Br1	0.98	2.80	3.617 (5)	142
C22—H22*A*⋯Br2^i^	0.98	2.97	3.739 (5)	137
C13—H13*C*⋯Br4^ii^	0.98	2.95	3.859 (5)	154
C21—H21*A*⋯Br4^iii^	0.98	2.85	3.785 (5)	160

Symmetry codes: (i) 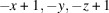 ; (ii) 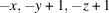 ; (iii) 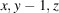 .

**Table 30:** Hydrogen-bond geometry (Å, °) for **21b** [Chem scheme1]

*D*—H⋯*A*	*D*—H	H⋯*A*	*D*⋯*A*	*D*—H⋯*A*
C21—H21*B*⋯Br1	0.98	3.34	3.927 (4)	120
C3—H3⋯Br2^i^	1.00	3.27	3.788 (3)	114
C31—H31*C*⋯Br2^i^	0.98	3.08	3.836 (3)	135
C32—H32*A*⋯Br2^i^	0.98	2.96	3.740 (3)	137
C1—H1⋯Br4^i^	1.00	3.18	4.045 (3)	145
C21—H21*C*⋯Br4^ii^	0.98	3.08	3.953 (3)	149
C31—H31*B*⋯Br4^i^	0.98	3.03	3.972 (4)	162
C1—H1⋯Br5^i^	1.00	2.93	3.756 (4)	140
C22—H22*C*⋯Br5^iii^	0.98	3.15	3.893 (4)	134

Symmetry codes: (i) 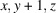 ; (ii) 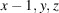 ; (iii) 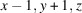 .

**Table 31:** Hydrogen-bond geometry (Å, °) for **22b** [Chem scheme1]

*D*—H⋯*A*	*D*—H	H⋯*A*	*D*⋯*A*	*D*—H⋯*A*
C13—H13*B*⋯Se1	0.98	2.70	3.167 (4)	109
C2—H2⋯Br1	1.00	3.05	3.540 (4)	112
C3—H3⋯Au2	1.00	2.85	3.783 (4)	155
C32—H32*C*⋯Br1	0.98	3.05	3.788 (4)	134
C21—H21*C*⋯Br3	0.98	3.03	3.848 (4)	142
C11—H11*A*⋯Br5	0.98	3.05	3.787 (4)	133
C2—H2⋯Br2^i^	1.00	3.17	3.901 (4)	131
C22—H22*A*⋯Br3^ii^	0.98	2.92	3.764 (4)	144
C12—H12*B*⋯Br3^iii^	0.98	2.96	3.826 (4)	148
C13—H13*B*⋯Br3^iii^	0.98	3.15	4.051 (4)	153
C12—H12*A*⋯Br4^iv^	0.98	2.82	3.761 (4)	160
C13—H13*A*⋯Br4^v^	0.98	3.05	3.771 (5)	132

Symmetry codes: (i) 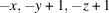 ; (ii) 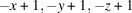 ; (iii) 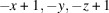 ; (iv) 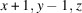 ; (v) 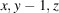 .

**Table 32:** Hydrogen-bond geometry (Å, °) for **23b** [Chem scheme1]

*D*—H⋯*A*	*D*—H	H⋯*A*	*D*⋯*A*	*D*—H⋯*A*
C13—H13*A*⋯Se1	0.98	2.60	3.163 (8)	116
C31—H31*B*⋯Br1	0.98	2.78	3.551 (7)	137
C23—H23*B*⋯Br1	0.98	2.98	3.476 (8)	112
C3—H3⋯Br3	1.00	3.13	4.108 (7)	166
C23—H23*C*⋯Br3	0.98	3.05	3.994 (9)	161
C11—H11*C*⋯Br4	0.98	3.23	4.182 (7)	165
C31—H31*C*⋯Br2^i^	0.98	3.06	3.825 (7)	136
C23—H23*A*⋯Br4^ii^	0.98	2.91	3.824 (7)	156
C13—H13*C*⋯Br4^iii^	0.98	3.06	3.998 (8)	160
C32—H32*C*⋯Br4^i^	0.98	3.01	3.782 (7)	136
C11—H11*A*⋯Br5^iii^	0.98	3.12	3.874 (7)	135
C32—H32*C*⋯Br5^iv^	0.98	2.93	3.804 (8)	149

Symmetry codes: (i) 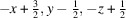 ; (ii) 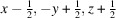 ; (iii) 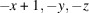 ; (iv) 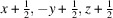 .

**Table:** 

	**17a**	**18a**	**19a**	**20a**	**21a**
Crystal data					
Chemical formula	(C_9_H_21_ClPS)[AuCl_4_]	(C_10_H_23_ClPS)[AuCl_4_]	(C_11_H_25_ClPS)[AuCl_4_]	(C_12_H_27_ClPS)[AuCl_4_]	(C_9_H_21_ClPSe)[AuCl_4_]
*M*_r_	566.50	580.53	594.55	608.58	613.40
Crystal system, space group	Monoclinic, *P*2_1_/*n*	Triclinic, *P* 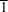	Monoclinic, *C*2/*c*	Monoclinic, *P*2_1_/*n*	Triclinic, *P* 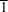
Temperature (K)	100	100	100	100	100
*a*, *b*, *c* (Å)	13.3577 (5), 10.0877 (3), 13.9182 (5)	7.5619 (4), 8.7408 (4), 15.4558 (6)	33.653 (3), 7.7933 (3), 16.212 (2)	9.7661 (3), 12.5787 (3), 16.9776 (5)	7.4202 (3), 8.5966 (3), 15.3980 (6)
α, β, γ (°)	90, 106.290 (4), 90	84.346 (4), 78.306 (4), 66.915 (5)	90, 113.861 (8), 90	90, 103.887 (3), 90	97.497 (3), 100.128 (4), 109.324 (4)
*V* (Å^3^)	1800.18 (11)	920.07 (8)	3888.5 (6)	2024.67 (10)	893.42 (6)
*Z*	4	2	8	4	2
Radiation type	Mo *K*α	Mo *K*α	Mo *K*α	Mo *K*α	Mo *K*α
μ (mm^−1^)	9.10	8.90	8.43	8.10	11.09
Crystal size (mm)	0.2 × 0.1 × 0.03	0.2 × 0.18 × 0.05	0.2 × 0.02 × 0.01	0.15 × 0.07 × 0.04	0.18 × 0.15 × 0.10

Data collection					
Diffractometer	Oxford Diffraction Xcalibur, Eos	Oxford Diffraction Xcalibur, Eos	Oxford Diffraction Xcalibur, Eos	Oxford Diffraction Xcalibur, Eos	Oxford Diffraction Xcalibur, Eos
Absorption correction	Multi-scan (*CrysAlis PRO*; Rigaku OD, 2015 ▸)	Multi-scan (*CrysAlis PRO*; Rigaku OD, 2015 ▸)	Multi-scan (*CrysAlis PRO*; Rigaku OD, 2015 ▸)	Multi-scan (*CrysAlis PRO*; Rigaku OD, 2015 ▸)	Multi-scan (*CrysAlis PRO*; Rigaku OD, 2015 ▸)
*T*_min_, *T*_max_	0.359, 1.000	0.316, 1.000	0.698, 1.000	0.756, 1.000	0.631, 1.000
No. of measured, independent and observed [*I* > 2σ(*I*)] reflections	65964, 4458, 3807	50339, 5453, 4838	84613, 4815, 3861	8891, 8891, 4196	106851, 5322, 4900
*R*_int_	0.056	0.032	0.096	–	0.039
θ values (°)	θ_max_ = 28.3, θ_min_ = 2.5	θ_max_ = 30.8, θ_min_ = 2.5	θ_max_ = 28.3, θ_min_ = 2.5	θ_max_ = 31.3, θ_min_ = 2.2	θ_max_ = 30.9, θ_min_ = 2.6
(sin θ/λ)_max_ (Å^−1^)	0.667	0.721	0.667	0.731	0.722

Refinement					
*R*[*F* ^2^ > 2σ(*F* ^2^)], *wR*(*F* ^2^), *S*	0.040, 0.102, 1.08	0.017, 0.036, 1.05	0.033, 0.064, 1.04	0.022, 0.045, 0.77	0.016, 0.033, 1.10
No. of reflections	4458	5453	4815	8891	5322
No. of parameters	160	174	191	194	164
No. of restraints	0	0	1	0	0
H-atom treatment	H-atom parameters constrained	H-atom parameters constrained	H-atom parameters constrained	H-atom parameters constrained	H-atom parameters constrained
Δρ_max_, Δρ_min_ (e Å^−3^)	5.27, −1.24	1.32, −1.07	2.43, −1.87	1.12, −0.92	0.83, −0.85
Extinction method	None	*F*_c_^*^ = *kF*_c_[1 + 0.001 *F*_c_^2^λ^3^/sin(2θ)]^-1/4^ (*SHELXL2019/3*; Sheldrick, 2015 ▸)	None	None	*F*_c_^*^ = *kF*_c_[1 + 0.001 *F*_c_^2^λ^3^/sin(2θ)]^-1/4^ (*SHELXL2019/3*; Sheldrick, 2015 ▸)
Extinction coefficient	–	0.00167 (11)	–	–	0.00346 (11)

**Table:** 

	**22a**	**23a**	**17b**	**18b**	**19b**
Crystal data					
Chemical formula	(C_10_H_23_ClPSe)[AuCl_4_]	(C_11_H_25_ClPSe)[AuCl_4_]	(C_9_H_21_BrPS)[AuBr_4_]	(C_10_H_23_BrPS)[AuBr_4_]	(C_11_H_25_BrPS)[AuBr_4_]
*M*_r_	627.43	641.46	788.80	802.83	816.86
Crystal system, space group	Triclinic, *P* 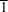	Monoclinic, *C*2/*c*	Triclinic, *P* 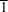	Triclinic, *P* 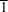	Monoclinic, *P*2_1_/*n*
Temperature (K)	100	100	100	100	100
*a*, *b*, *c* (Å)	7.5836 (2), 8.7603 (2), 15.4719 (6)	33.7472 (6), 7.79306 (8), 16.2575 (3)	7.8543 (4), 8.0592 (4), 15.3505 (7)	7.8455 (6), 9.1380 (6), 15.5440 (9)	12.4712 (4), 10.3712 (3), 16.2524 (5)
α, β, γ (°)	84.445 (3), 78.641 (3), 67.128 (3)	90, 113.582 (3), 90	76.717 (4), 83.169 (4), 87.722 (4)	86.600 (5), 81.097 (6), 64.862 (6)	90, 92.724 (3), 90
*V* (Å^3^)	928.28 (5)	3918.54 (14)	938.89 (8)	996.65 (13)	2099.73 (11)
*Z*	2	8	2	2	4
Radiation type	Mo *K*α	Mo *K*α	Mo *K*α	Mo *K*α	Mo *K*α
μ (mm^−1^)	10.67	10.12	18.65	17.57	16.69
Crystal size (mm)	0.2 × 0.2 × 0.03	0.35 × 0.2 × 0.04	0.2 × 0.08 × 0.05	0.25 × 0.1 × 0.03	0.35 × 0.25 × 0.10

Data collection					
Diffractometer	Oxford Diffraction Xcalibur, Eos	Oxford Diffraction Xcalibur, Eos	Oxford Diffraction Xcalibur, Eos	Oxford Diffraction Xcalibur, Eos	Oxford Diffraction Xcalibur, Eos
Absorption correction	Multi-scan (*CrysAlis PRO*; Rigaku OD, 2015 ▸)	Multi-scan (*CrysAlis PRO*; Rigaku OD, 2015 ▸)	Multi-scan (*CrysAlis PRO*; Rigaku OD, 2015 ▸)	Multi-scan (*CrysAlis PRO*; Rigaku OD, 2015 ▸)	Multi-scan (*CrysAlis PRO*; Rigaku OD, 2015 ▸)
*T*_min_, *T*_max_	0.232, 1.000	0.308, 1.000	0.247, 1.000	0.097, 0.621	0.068, 0.286
No. of measured, independent and observed [*I* > 2σ(*I*)] reflections	102916, 5490, 4978	257492, 6028, 5453	54229, 5646, 5089	26487, 5731, 5030	70058, 6335, 5041
*R*_int_	0.042	0.058	0.043	0.045	0.065
θ values (°)	θ_max_ = 30.9, θ_min_ = 2.5	θ_max_ = 30.9, θ_min_ = 2.5	θ_max_ = 30.9, θ_min_ = 2.6	θ_max_ = 30.8, θ_min_ = 2.5	θ_max_ = 30.9, θ_min_ = 2.3
(sin θ/λ)_max_ (Å^−1^)	0.722	0.723	0.721	0.720	0.723

Refinement					
*R*[*F* ^2^ > 2σ(*F* ^2^)], *wR*(*F* ^2^), *S*	0.021, 0.054, 1.05	0.021, 0.043, 1.11	0.021, 0.040, 1.08	0.027, 0.052, 1.06	0.033, 0.061, 1.09
No. of reflections	5490	6028	5646	5731	6335
No. of parameters	174	191	164	173	183
No. of restraints	0	0	0	0	0
H-atom treatment	H-atom parameters constrained	H-atom parameters constrained	H-atom parameters constrained	H-atom parameters constrained	H-atom parameters constrained
Δρ_max_, Δρ_min_ (e Å^−3^)	1.86, −1.59	1.13, −1.20	1.42, −1.15	2.15, −2.24	2.43, −1.44
Extinction method	*F*_c_^*^ = *kF*_c_[1 + 0.001 *F*_c_^2^λ^3^/sin(2θ)]^-1/4^ (*SHELXL2019/3*; Sheldrick, 2015 ▸)	None	*F*_c_^*^ = *kF*_c_[1 + 0.001 *F*_c_^2^λ^3^/sin(2θ)]^-1/4^ (*SHELXL2019/3*; Sheldrick, 2015 ▸)	None	None
Extinction coefficient	0.00144 (18)	–	0.00112 (7)	–	–

**Table:** 

	**20b**	**21b**	**22b**	**23b**
Crystal data				
Chemical formula	(C_12_H_27_BrPS)[AuBr_4_]	(C_9_H_21_BrPSe)[AuBr_4_]	(C_10_H_23_BrPSe)[AuBr_4_]	(C_11_H_25_BrPSe)[AuBr_4_]
*M*_r_	830.88	835.70	849.73	863.76
Crystal system, space group	Triclinic, *P* 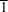	Triclinic, *P* 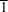	Triclinic, *P* 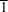	Monoclinic, *P*2_1_/*n*
Temperature (K)	100	100	100	101
*a*, *b*, *c* (Å)	10.2218 (5), 10.8085 (6), 11.1163 (5)	7.9212 (4), 8.0606 (4), 15.2911 (8)	7.8155 (3), 9.1505 (3), 15.5221 (5)	12.3529 (4), 10.4233 (4), 16.4635 (5)
α, β, γ (°)	70.909 (4), 71.516 (5), 75.645 (4)	76.817 (5), 82.668 (5), 87.728 (4)	85.965 (2), 80.294 (3), 66.049 (3)	90, 93.453 (3), 90
*V* (Å^3^)	1086.40 (10)	942.78 (9)	999.97 (6)	2115.97 (12)
*Z*	2	2	2	4
Radiation type	Mo *K*α	Mo *K*α	Mo *K*α	Mo *K*α
μ (mm^−1^)	16.13	20.39	19.23	18.18
Crystal size (mm)	0.1 × 0.05 × 0.002	0.1 × 0.1 × 0.05	0.3 × 0.2 × 0.03	0.2 × 0.1 × 0.02

Data collection				
Diffractometer	Oxford Diffraction Xcalibur, Eos	Oxford Diffraction Xcalibur, Eos	Oxford Diffraction Xcalibur, Eos	Oxford Diffraction Xcalibur, Eos
Absorption correction	Multi-scan (*CrysAlis PRO*; Rigaku OD, 2015 ▸)	Multi-scan (*CrysAlis PRO*; Rigaku OD, 2015 ▸)	Multi-scan (*CrysAlis PRO*; Rigaku OD, 2015 ▸)	Multi-scan (*CrysAlis PRO*; Rigaku OD, 2015 ▸)
*T*_min_, *T*_max_	0.284, 1.000	0.434, 1.000	0.202, 1.000	0.122, 0.713
No. of measured, independent and observed [*I* > 2σ(*I*)] reflections	73343, 6283, 5151	69642, 5661, 4881	52431, 5847, 5198	7063, 7063, 4089
*R*_int_	0.078	0.057	0.050	–
θ values (°)	θ_max_ = 30.0, θ_min_ = 2.4	θ_max_ = 30.9, θ_min_ = 2.6	θ_max_ = 30.9, θ_min_ = 2.4	θ_max_ = 28.3, θ_min_ = 2.3
(sin θ/λ)_max_ (Å^−1^)	0.704	0.723	0.722	0.667

Refinement				
*R*[*F* ^2^ > 2σ(*F* ^2^)], *wR*(*F* ^2^), *S*	0.032, 0.066, 1.05	0.024, 0.045, 1.06	0.025, 0.063, 1.04	0.034, 0.061, 0.81
No. of reflections	6283	5661	5847	7063
No. of parameters	193	164	173	184
No. of restraints	0	0	0	66
H-atom treatment	H-atom parameters constrained	H-atom parameters constrained	H-atom parameters constrained	H-atom parameters constrained
Δρ_max_, Δρ_min_ (e Å^−3^)	1.56, −1.67	1.53, −1.01	1.71, −1.44	1.72, −1.10
Extinction method	None	*F*_c_^*^ = *kF*_c_[1 + 0.001 *F*_c_^2^λ^3^/sin(2θ)]^-1/4^ (*SHELXL2019/3*; Sheldrick, 2015 ▸)	None	None
Extinction coefficient	–	0.00116 (7)	–	–

Computer programs: *CrysAlis PRO* (Rigaku OD, 2015 ▸), *SHELXS97* (Sheldrick, 2008 ▸), *SHELXL2019/3* (Sheldrick, 2015 ▸) and *XP* (Bruker, 1998 ▸).

## supplementary crystallographic information

### (Chlorosulfanyl)tris(propan-2-yl)phosphonium tetrachloridoaurate(III) (17a) Crystal data

**Table:**

(C_9_H_21_ClPS)[AuCl_4_]	*F*(000) = 1080
*M**_r_* = 566.50	*D*_x_ = 2.090 Mg m^−^^3^
Monoclinic, *P*2_1_/*n*	Mo *K*α radiation, λ = 0.71073 Å
*a* = 13.3577 (5) Å	Cell parameters from 13392 reflections
*b* = 10.0877 (3) Å	θ = 2.5–27.5°
*c* = 13.9182 (5) Å	µ = 9.10 mm^−^^1^
β = 106.290 (4)°	*T* = 100 K
*V* = 1800.18 (11) Å^3^	Plate, yellow
*Z* = 4	0.2 × 0.1 × 0.03 mm

### (Chlorosulfanyl)tris(propan-2-yl)phosphonium tetrachloridoaurate(III) (17a) Data collection

**Table:**

Oxford Diffraction Xcalibur, Eos diffractometer	4458 independent reflections
Radiation source: fine-focus sealed tube	3807 reflections with *I* > 2σ(*I*)
Detector resolution: 16.1419 pixels mm^-1^	*R*_int_ = 0.056
ω scan	θ_max_ = 28.3°, θ_min_ = 2.5°
Absorption correction: multi-scan (CrysAlisPro; Rigaku OD, 2015)	*h* = −17→17
*T*_min_ = 0.359, *T*_max_ = 1.000	*k* = −13→13
65964 measured reflections	*l* = −18→18

### (Chlorosulfanyl)tris(propan-2-yl)phosphonium tetrachloridoaurate(III) (17a) Refinement

**Table:**

Refinement on *F*^2^	Primary atom site location: structure-invariant direct methods
Least-squares matrix: full	Secondary atom site location: difference Fourier map
*R*[*F*^2^ > 2σ(*F*^2^)] = 0.040	Hydrogen site location: inferred from neighbouring sites
*wR*(*F*^2^) = 0.102	H-atom parameters constrained
*S* = 1.08	*w* = 1/[σ^2^(*F*_o_^2^) + (0.0437*P*)^2^ + 16.018*P*] where *P* = (*F*_o_^2^ + 2*F*_c_^2^)/3
4458 reflections	(Δ/σ)_max_ < 0.001
160 parameters	Δρ_max_ = 5.27 e Å^−^^3^
0 restraints	Δρ_min_ = −1.24 e Å^−^^3^

### (Chlorosulfanyl)tris(propan-2-yl)phosphonium tetrachloridoaurate(III) (17a) Special details

**Table:**

Refinement. The structure can also be refined with a second position of the AuCl4 anion, occupation ca. 3%, which leads to an improved R factor, but is probably just a way of removing the excess residual electron density at the gold atom, in turn probably caused by residual absorption errors.

### (Chlorosulfanyl)tris(propan-2-yl)phosphonium tetrachloridoaurate(III) (17a) Fractional atomic coordinates and isotropic or equivalent isotropic displacement parameters (Å^2^)

**Table:**

	*x*	*y*	*z*	*U*_iso_*/*U*_eq_	
P1	0.49492 (13)	0.26458 (15)	0.71127 (13)	0.0229 (3)	
S1	0.37078 (15)	0.3269 (2)	0.59158 (16)	0.0411 (5)	
Cl1	0.4167 (2)	0.24787 (19)	0.47722 (16)	0.0518 (6)	
C1	0.4821 (6)	0.3685 (7)	0.8153 (6)	0.0332 (15)	
H1	0.524753	0.449958	0.815124	0.040*	
C2	0.6151 (5)	0.3045 (7)	0.6810 (5)	0.0283 (14)	
H2	0.617064	0.249462	0.621766	0.034*	
C3	0.4873 (5)	0.0874 (6)	0.7358 (5)	0.0244 (12)	
H3	0.538296	0.069314	0.802265	0.029*	
C11	0.5284 (6)	0.3003 (7)	0.9151 (6)	0.0366 (16)	
H11A	0.524432	0.359834	0.969554	0.055*	
H11B	0.601537	0.278020	0.922051	0.055*	
H11C	0.489247	0.219045	0.918159	0.055*	
C12	0.3706 (7)	0.4145 (8)	0.8027 (7)	0.047 (2)	
H12A	0.325891	0.337373	0.802076	0.071*	
H12B	0.345490	0.463001	0.739501	0.071*	
H12C	0.368791	0.472870	0.858506	0.071*	
C21	0.6189 (6)	0.4495 (7)	0.6512 (6)	0.0394 (17)	
H21A	0.617945	0.506362	0.707997	0.059*	
H21B	0.558271	0.469518	0.594537	0.059*	
H21C	0.682984	0.465715	0.631895	0.059*	
C22	0.7085 (5)	0.2646 (7)	0.7673 (5)	0.0319 (15)	
H22A	0.772495	0.272582	0.746412	0.048*	
H22B	0.700199	0.172606	0.786402	0.048*	
H22C	0.713043	0.322906	0.824699	0.048*	
C31	0.3807 (6)	0.0498 (7)	0.7447 (6)	0.0351 (16)	
H31A	0.328664	0.062437	0.679948	0.053*	
H31B	0.363197	0.105981	0.795134	0.053*	
H31C	0.381162	−0.043333	0.764827	0.053*	
C32	0.5206 (6)	0.0026 (6)	0.6583 (5)	0.0297 (14)	
H32A	0.522900	−0.090931	0.677830	0.044*	
H32B	0.589870	0.030604	0.655174	0.044*	
H32C	0.470257	0.014050	0.592388	0.044*	
Au1	0.19556 (2)	0.73810 (2)	0.47234 (2)	0.02350 (9)	
Cl2	0.34024 (14)	0.70385 (17)	0.60253 (15)	0.0379 (4)	
Cl3	0.24567 (14)	0.95147 (16)	0.45821 (13)	0.0325 (4)	
Cl4	0.04849 (14)	0.77264 (18)	0.34302 (14)	0.0355 (4)	
Cl5	0.14653 (14)	0.52388 (16)	0.48526 (14)	0.0341 (4)	

### (Chlorosulfanyl)tris(propan-2-yl)phosphonium tetrachloridoaurate(III) (17a) Atomic displacement parameters (Å^2^)

**Table:**

	*U*^11^	*U*^22^	*U*^33^	*U*^12^	*U*^13^	*U*^23^
P1	0.0234 (8)	0.0195 (7)	0.0242 (8)	0.0026 (6)	0.0038 (6)	−0.0009 (6)
S1	0.0346 (10)	0.0362 (10)	0.0437 (11)	0.0058 (8)	−0.0031 (8)	0.0093 (8)
Cl1	0.0764 (15)	0.0362 (10)	0.0309 (10)	−0.0012 (9)	−0.0046 (10)	0.0011 (7)
C1	0.043 (4)	0.022 (3)	0.038 (4)	−0.006 (3)	0.017 (3)	−0.004 (3)
C2	0.028 (3)	0.025 (3)	0.033 (4)	−0.004 (3)	0.012 (3)	−0.007 (3)
C3	0.025 (3)	0.020 (3)	0.025 (3)	−0.001 (2)	0.002 (2)	0.000 (2)
C11	0.048 (4)	0.027 (3)	0.040 (4)	0.000 (3)	0.021 (3)	0.001 (3)
C12	0.051 (5)	0.041 (4)	0.063 (6)	0.017 (4)	0.037 (4)	0.011 (4)
C21	0.049 (4)	0.027 (4)	0.047 (4)	−0.011 (3)	0.021 (4)	−0.002 (3)
C22	0.026 (3)	0.040 (4)	0.031 (4)	−0.005 (3)	0.011 (3)	−0.010 (3)
C31	0.037 (4)	0.031 (4)	0.040 (4)	−0.009 (3)	0.014 (3)	−0.004 (3)
C32	0.039 (4)	0.018 (3)	0.031 (3)	−0.001 (3)	0.010 (3)	−0.004 (2)
Au1	0.02594 (14)	0.02090 (13)	0.02427 (14)	−0.00120 (9)	0.00804 (9)	−0.00287 (9)
Cl2	0.0324 (9)	0.0263 (8)	0.0454 (10)	−0.0038 (7)	−0.0051 (7)	0.0020 (7)
Cl3	0.0371 (9)	0.0226 (7)	0.0358 (9)	−0.0045 (6)	0.0069 (7)	0.0022 (6)
Cl4	0.0361 (9)	0.0334 (8)	0.0307 (8)	−0.0025 (7)	−0.0008 (7)	−0.0002 (7)
Cl5	0.0357 (9)	0.0234 (7)	0.0398 (9)	−0.0068 (6)	0.0049 (7)	−0.0012 (6)

### (Chlorosulfanyl)tris(propan-2-yl)phosphonium tetrachloridoaurate(III) (17a) Geometric parameters (Å, º)

**Table:**

P1—C2	1.815 (7)	C12—H12B	0.9800
P1—C3	1.828 (6)	C12—H12C	0.9800
P1—C1	1.834 (7)	C21—H21A	0.9800
P1—S1	2.091 (2)	C21—H21B	0.9800
S1—Cl1	2.023 (3)	C21—H21C	0.9800
S1—Cl5	3.553 (3)	C22—H22A	0.9800
C1—C11	1.518 (11)	C22—H22B	0.9800
C1—C12	1.522 (11)	C22—H22C	0.9800
C1—H1	1.0000	C31—H31A	0.9800
C2—C22	1.525 (10)	C31—H31B	0.9800
C2—C21	1.525 (10)	C31—H31C	0.9800
C2—H2	1.0000	C32—H32A	0.9800
C3—C31	1.511 (9)	C32—H32B	0.9800
C3—C32	1.537 (9)	C32—H32C	0.9800
C3—H3	1.0000	Au1—Cl2	2.2745 (17)
C11—H11A	0.9800	Au1—Cl3	2.2789 (16)
C11—H11B	0.9800	Au1—Cl5	2.2797 (16)
C11—H11C	0.9800	Au1—Cl4	2.2884 (17)
C12—H12A	0.9800		
			
C2—P1—C3	111.1 (3)	H12A—C12—H12B	109.5
C2—P1—C1	109.9 (3)	C1—C12—H12C	109.5
C3—P1—C1	112.9 (3)	H12A—C12—H12C	109.5
C2—P1—S1	107.7 (2)	H12B—C12—H12C	109.5
C3—P1—S1	111.7 (2)	C2—C21—H21A	109.5
C1—P1—S1	103.2 (3)	C2—C21—H21B	109.5
Cl1—S1—P1	99.25 (11)	H21A—C21—H21B	109.5
Cl1—S1—Cl5	107.39 (10)	C2—C21—H21C	109.5
P1—S1—Cl5	152.59 (11)	H21A—C21—H21C	109.5
C11—C1—C12	112.2 (6)	H21B—C21—H21C	109.5
C11—C1—P1	111.0 (5)	C2—C22—H22A	109.5
C12—C1—P1	112.9 (6)	C2—C22—H22B	109.5
C11—C1—H1	106.8	H22A—C22—H22B	109.5
C12—C1—H1	106.8	C2—C22—H22C	109.5
P1—C1—H1	106.8	H22A—C22—H22C	109.5
C22—C2—C21	112.6 (6)	H22B—C22—H22C	109.5
C22—C2—P1	109.9 (5)	C3—C31—H31A	109.5
C21—C2—P1	112.0 (5)	C3—C31—H31B	109.5
C22—C2—H2	107.3	H31A—C31—H31B	109.5
C21—C2—H2	107.3	C3—C31—H31C	109.5
P1—C2—H2	107.3	H31A—C31—H31C	109.5
C31—C3—C32	112.5 (5)	H31B—C31—H31C	109.5
C31—C3—P1	111.3 (5)	C3—C32—H32A	109.5
C32—C3—P1	111.8 (4)	C3—C32—H32B	109.5
C31—C3—H3	107.0	H32A—C32—H32B	109.5
C32—C3—H3	107.0	C3—C32—H32C	109.5
P1—C3—H3	107.0	H32A—C32—H32C	109.5
C1—C11—H11A	109.5	H32B—C32—H32C	109.5
C1—C11—H11B	109.5	Cl2—Au1—Cl3	90.44 (6)
H11A—C11—H11B	109.5	Cl2—Au1—Cl5	89.54 (6)
C1—C11—H11C	109.5	Cl3—Au1—Cl5	179.34 (7)
H11A—C11—H11C	109.5	Cl2—Au1—Cl4	179.13 (7)
H11B—C11—H11C	109.5	Cl3—Au1—Cl4	89.80 (6)
C1—C12—H12A	109.5	Cl5—Au1—Cl4	90.24 (6)
C1—C12—H12B	109.5	Au1—Cl5—S1	109.61 (7)
			
C2—P1—S1—Cl1	48.2 (3)	C3—P1—C2—C22	−59.0 (5)
C3—P1—S1—Cl1	−74.0 (3)	C1—P1—C2—C22	66.7 (5)
C1—P1—S1—Cl1	164.4 (2)	S1—P1—C2—C22	178.4 (4)
C2—P1—S1—Cl5	−118.1 (3)	C3—P1—C2—C21	174.9 (5)
C3—P1—S1—Cl5	119.7 (3)	C1—P1—C2—C21	−59.4 (6)
C1—P1—S1—Cl5	−1.9 (3)	S1—P1—C2—C21	52.4 (5)
C2—P1—C1—C11	−92.0 (6)	C2—P1—C3—C31	−170.9 (5)
C3—P1—C1—C11	32.6 (6)	C1—P1—C3—C31	65.1 (6)
S1—P1—C1—C11	153.4 (5)	S1—P1—C3—C31	−50.7 (5)
C2—P1—C1—C12	141.0 (5)	C2—P1—C3—C32	−44.1 (5)
C3—P1—C1—C12	−94.4 (6)	C1—P1—C3—C32	−168.2 (5)
S1—P1—C1—C12	26.4 (6)	S1—P1—C3—C32	76.1 (5)

### (Chlorosulfanyl)tris(propan-2-yl)phosphonium tetrachloridoaurate(III) (17a) Hydrogen-bond geometry (Å, º)

**Table:**

*D*—H···*A*	*D*—H	H···*A*	*D*···*A*	*D*—H···*A*
C2—H2···Cl1	1.00	2.86	3.343 (7)	110
C32—H32*C*···Cl1	0.98	2.83	3.527 (7)	129
C12—H12*B*···S1	0.98	2.57	3.069 (9)	111
C21—H21*B*···S1	0.98	2.88	3.415 (8)	115
C32—H32*A*···Cl1^i^	0.98	2.97	3.395 (7)	108
C22—H22*C*···Cl3^ii^	0.98	2.89	3.843 (7)	164
C1—H1···Cl4^ii^	1.00	2.83	3.721 (7)	149
C32—H32*A*···Cl4^iii^	0.98	2.88	3.731 (7)	145
C3—H3···Cl5^iii^	1.00	2.72	3.701 (7)	167

Symmetry codes: (i) −*x*+1, −*y*, −*z*+1; (ii) *x*+1/2, −*y*+3/2, *z*+1/2; (iii) *x*+1/2, −*y*+1/2, *z*+1/2.

### (tert-Butyl)(chlorosulfanyl)bis(propan-2-yl)phosphonium tetrachloridoaurate(III) (18a) Crystal data

**Table:**

(C_10_H_23_ClPS)[AuCl_4_]	*Z* = 2
*M**_r_* = 580.53	*F*(000) = 556
Triclinic, *P*1	*D*_x_ = 2.095 Mg m^−^^3^
*a* = 7.5619 (4) Å	Mo *K*α radiation, λ = 0.71073 Å
*b* = 8.7408 (4) Å	Cell parameters from 21451 reflections
*c* = 15.4558 (6) Å	θ = 2.5–30.7°
α = 84.346 (4)°	µ = 8.90 mm^−^^1^
β = 78.306 (4)°	*T* = 100 K
γ = 66.915 (5)°	Plate, yellow
*V* = 920.07 (8) Å^3^	0.2 × 0.18 × 0.05 mm

### (tert-Butyl)(chlorosulfanyl)bis(propan-2-yl)phosphonium tetrachloridoaurate(III) (18a) Data collection

**Table:**

Oxford Diffraction Xcalibur, Eos diffractometer	5453 independent reflections
Radiation source: Enhance (Mo) X-ray Source	4838 reflections with *I* > 2σ(*I*)
Detector resolution: 16.1419 pixels mm^-1^	*R*_int_ = 0.032
ω scan	θ_max_ = 30.8°, θ_min_ = 2.5°
Absorption correction: multi-scan (CrysAlisPro; Rigaku OD, 2015)	*h* = −10→10
*T*_min_ = 0.316, *T*_max_ = 1.000	*k* = −12→12
50339 measured reflections	*l* = −22→22

### (tert-Butyl)(chlorosulfanyl)bis(propan-2-yl)phosphonium tetrachloridoaurate(III) (18a) Refinement

**Table:**

Refinement on *F*^2^	Secondary atom site location: difference Fourier map
Least-squares matrix: full	Hydrogen site location: inferred from neighbouring sites
*R*[*F*^2^ > 2σ(*F*^2^)] = 0.017	H-atom parameters constrained
*wR*(*F*^2^) = 0.036	*w* = 1/[σ^2^(*F*_o_^2^) + (0.0146*P*)^2^ + 0.7146*P*] where *P* = (*F*_o_^2^ + 2*F*_c_^2^)/3
*S* = 1.05	(Δ/σ)_max_ < 0.001
5453 reflections	Δρ_max_ = 1.32 e Å^−^^3^
174 parameters	Δρ_min_ = −1.07 e Å^−^^3^
0 restraints	Extinction correction: *SHELXL2019/3* (Sheldrick, 2015), *F*_c_^*^ = *kF*_c_[1 + 0.001 *F*_c_^2^λ^3^/sin(2θ)]^-1/4^
Primary atom site location: structure-invariant direct methods	Extinction coefficient: 0.00167 (11)

### (tert-Butyl)(chlorosulfanyl)bis(propan-2-yl)phosphonium tetrachloridoaurate(III) (18a) Fractional atomic coordinates and isotropic or equivalent isotropic displacement parameters (Å^2^)

**Table:**

	*x*	*y*	*z*	*U*_iso_*/*U*_eq_	
P1	0.50154 (7)	0.10754 (6)	0.25334 (3)	0.01224 (10)	
S1	0.38459 (8)	−0.05864 (6)	0.32441 (4)	0.01794 (10)	
Cl1	0.16037 (8)	0.09519 (6)	0.41119 (4)	0.02207 (11)	
C1	0.7444 (3)	−0.0351 (2)	0.19677 (13)	0.0153 (4)	
C2	0.5052 (3)	0.2531 (3)	0.32961 (13)	0.0177 (4)	
H2	0.365008	0.322876	0.351735	0.021*	
C3	0.3485 (3)	0.2313 (2)	0.17343 (13)	0.0150 (4)	
H3	0.425026	0.289589	0.133508	0.018*	
C11	0.8102 (3)	0.0503 (3)	0.11176 (14)	0.0202 (4)	
H11A	0.810597	0.156811	0.126276	0.030*	
H11B	0.719773	0.069885	0.070783	0.030*	
H11C	0.942123	−0.021278	0.084058	0.030*	
C12	0.8927 (3)	−0.0766 (3)	0.25912 (14)	0.0207 (4)	
H12A	1.017861	−0.159937	0.232013	0.031*	
H12B	0.843556	−0.120881	0.315429	0.031*	
H12C	0.911108	0.024451	0.269613	0.031*	
C13	0.7340 (3)	−0.1982 (2)	0.17357 (15)	0.0206 (4)	
H13A	0.628937	−0.172829	0.139798	0.031*	
H13B	0.707756	−0.259176	0.228090	0.031*	
H13C	0.858777	−0.266419	0.138057	0.031*	
C21	0.5927 (4)	0.1771 (3)	0.41227 (15)	0.0272 (5)	
H21A	0.735554	0.130546	0.396580	0.041*	
H21B	0.546943	0.088628	0.436873	0.041*	
H21C	0.551478	0.263411	0.456305	0.041*	
C22	0.5945 (4)	0.3744 (3)	0.27927 (16)	0.0242 (5)	
H22A	0.576412	0.462365	0.318885	0.036*	
H22B	0.529594	0.424156	0.228770	0.036*	
H22C	0.734407	0.314002	0.258130	0.036*	
C31	0.3042 (3)	0.1241 (3)	0.11454 (15)	0.0229 (5)	
H31A	0.231644	0.062428	0.151359	0.034*	
H31B	0.427100	0.045502	0.082497	0.034*	
H31C	0.225641	0.195878	0.072150	0.034*	
C32	0.1580 (3)	0.3670 (3)	0.21726 (15)	0.0216 (4)	
H32A	0.083732	0.431051	0.171716	0.032*	
H32B	0.189133	0.441441	0.249630	0.032*	
H32C	0.079827	0.315144	0.258369	0.032*	
Au1	0.000000	0.500000	0.500000	0.01355 (3)	
Cl2	−0.16568 (8)	0.34352 (7)	0.57470 (4)	0.02303 (11)	
Cl3	0.21372 (8)	0.41221 (7)	0.59592 (4)	0.02613 (12)	
Au2	0.500000	0.500000	0.000000	0.01239 (3)	
Cl4	0.34494 (8)	0.70229 (6)	0.10234 (4)	0.02293 (11)	
Cl5	0.79512 (7)	0.47158 (6)	0.02926 (4)	0.02032 (10)	

### (tert-Butyl)(chlorosulfanyl)bis(propan-2-yl)phosphonium tetrachloridoaurate(III) (18a) Atomic displacement parameters (Å^2^)

**Table:**

	*U*^11^	*U*^22^	*U*^33^	*U*^12^	*U*^13^	*U*^23^
P1	0.0107 (2)	0.0125 (2)	0.0131 (2)	−0.00431 (18)	−0.00189 (18)	−0.00008 (18)
S1	0.0162 (2)	0.0147 (2)	0.0212 (3)	−0.00623 (18)	−0.00016 (19)	0.00216 (19)
Cl1	0.0175 (2)	0.0233 (2)	0.0232 (3)	−0.0088 (2)	0.00370 (19)	−0.0011 (2)
C1	0.0118 (9)	0.0165 (9)	0.0148 (9)	−0.0033 (7)	−0.0010 (7)	0.0004 (7)
C2	0.0173 (10)	0.0222 (10)	0.0158 (10)	−0.0095 (8)	−0.0012 (8)	−0.0055 (8)
C3	0.0138 (9)	0.0129 (9)	0.0179 (10)	−0.0038 (7)	−0.0055 (8)	0.0023 (7)
C11	0.0157 (10)	0.0233 (10)	0.0175 (10)	−0.0055 (8)	0.0013 (8)	0.0016 (8)
C12	0.0126 (10)	0.0265 (11)	0.0200 (11)	−0.0042 (8)	−0.0034 (8)	0.0008 (8)
C13	0.0197 (10)	0.0143 (9)	0.0250 (11)	−0.0027 (8)	−0.0040 (9)	−0.0033 (8)
C21	0.0271 (12)	0.0406 (14)	0.0167 (11)	−0.0152 (11)	−0.0036 (9)	−0.0049 (10)
C22	0.0265 (12)	0.0249 (11)	0.0266 (12)	−0.0155 (9)	−0.0027 (9)	−0.0042 (9)
C31	0.0251 (11)	0.0194 (10)	0.0254 (12)	−0.0052 (9)	−0.0138 (9)	−0.0008 (9)
C32	0.0177 (10)	0.0165 (10)	0.0264 (12)	−0.0010 (8)	−0.0065 (9)	−0.0001 (8)
Au1	0.01509 (6)	0.01277 (5)	0.01290 (6)	−0.00503 (4)	−0.00292 (4)	−0.00121 (4)
Cl2	0.0239 (3)	0.0218 (2)	0.0256 (3)	−0.0125 (2)	−0.0035 (2)	0.0033 (2)
Cl3	0.0254 (3)	0.0343 (3)	0.0223 (3)	−0.0129 (2)	−0.0121 (2)	0.0057 (2)
Au2	0.00974 (5)	0.01046 (5)	0.01638 (6)	−0.00389 (4)	−0.00134 (4)	0.00042 (4)
Cl4	0.0181 (2)	0.0202 (2)	0.0277 (3)	−0.00397 (19)	−0.0005 (2)	−0.0092 (2)
Cl5	0.0134 (2)	0.0207 (2)	0.0283 (3)	−0.00719 (18)	−0.00544 (19)	0.0000 (2)

### (tert-Butyl)(chlorosulfanyl)bis(propan-2-yl)phosphonium tetrachloridoaurate(III) (18a) Geometric parameters (Å, º)

**Table:**

P1—C2	1.829 (2)	C13—H13B	0.9800
P1—C3	1.836 (2)	C13—H13C	0.9800
P1—C1	1.860 (2)	C21—H21A	0.9800
P1—S1	2.0970 (7)	C21—H21B	0.9800
S1—Cl1	2.0316 (7)	C21—H21C	0.9800
Cl1—Cl2	3.3964 (8)	C22—H22A	0.9800
C1—C11	1.538 (3)	C22—H22B	0.9800
C1—C13	1.538 (3)	C22—H22C	0.9800
C1—C12	1.539 (3)	C31—H31A	0.9800
C2—C21	1.530 (3)	C31—H31B	0.9800
C2—C22	1.540 (3)	C31—H31C	0.9800
C2—H2	1.0000	C32—H32A	0.9800
C3—C31	1.535 (3)	C32—H32B	0.9800
C3—C32	1.538 (3)	C32—H32C	0.9800
C3—H3	1.0000	Au1—Cl3^i^	2.2744 (5)
C11—H11A	0.9800	Au1—Cl3	2.2744 (5)
C11—H11B	0.9800	Au1—Cl2	2.2847 (5)
C11—H11C	0.9800	Au1—Cl2^i^	2.2847 (5)
C12—H12A	0.9800	Au2—Cl4^ii^	2.2753 (5)
C12—H12B	0.9800	Au2—Cl4	2.2753 (5)
C12—H12C	0.9800	Au2—Cl5	2.2801 (5)
C13—H13A	0.9800	Au2—Cl5^ii^	2.2801 (5)
			
C2—P1—C3	107.15 (9)	C1—C13—H13C	109.5
C2—P1—C1	115.86 (10)	H13A—C13—H13C	109.5
C3—P1—C1	111.39 (9)	H13B—C13—H13C	109.5
C2—P1—S1	109.35 (7)	C2—C21—H21A	109.5
C3—P1—S1	110.73 (7)	C2—C21—H21B	109.5
C1—P1—S1	102.30 (7)	H21A—C21—H21B	109.5
Cl1—S1—P1	101.57 (3)	C2—C21—H21C	109.5
S1—Cl1—Cl2	171.74 (3)	H21A—C21—H21C	109.5
C11—C1—C13	109.53 (17)	H21B—C21—H21C	109.5
C11—C1—C12	109.89 (17)	C2—C22—H22A	109.5
C13—C1—C12	108.50 (17)	C2—C22—H22B	109.5
C11—C1—P1	109.31 (14)	H22A—C22—H22B	109.5
C13—C1—P1	110.59 (14)	C2—C22—H22C	109.5
C12—C1—P1	109.01 (13)	H22A—C22—H22C	109.5
C21—C2—C22	112.40 (19)	H22B—C22—H22C	109.5
C21—C2—P1	116.51 (16)	C3—C31—H31A	109.5
C22—C2—P1	110.40 (14)	C3—C31—H31B	109.5
C21—C2—H2	105.5	H31A—C31—H31B	109.5
C22—C2—H2	105.5	C3—C31—H31C	109.5
P1—C2—H2	105.5	H31A—C31—H31C	109.5
C31—C3—C32	110.39 (17)	H31B—C31—H31C	109.5
C31—C3—P1	112.89 (14)	C3—C32—H32A	109.5
C32—C3—P1	112.80 (14)	C3—C32—H32B	109.5
C31—C3—H3	106.8	H32A—C32—H32B	109.5
C32—C3—H3	106.8	C3—C32—H32C	109.5
P1—C3—H3	106.8	H32A—C32—H32C	109.5
C1—C11—H11A	109.5	H32B—C32—H32C	109.5
C1—C11—H11B	109.5	Cl3^i^—Au1—Cl3	180.0
H11A—C11—H11B	109.5	Cl3^i^—Au1—Cl2	89.57 (2)
C1—C11—H11C	109.5	Cl3—Au1—Cl2	90.43 (2)
H11A—C11—H11C	109.5	Cl3^i^—Au1—Cl2^i^	90.43 (2)
H11B—C11—H11C	109.5	Cl3—Au1—Cl2^i^	89.57 (2)
C1—C12—H12A	109.5	Cl2—Au1—Cl2^i^	180.000 (18)
C1—C12—H12B	109.5	Au1—Cl2—Cl1	75.206 (18)
H12A—C12—H12B	109.5	Cl4^ii^—Au2—Cl4	180.0
C1—C12—H12C	109.5	Cl4^ii^—Au2—Cl5	89.65 (2)
H12A—C12—H12C	109.5	Cl4—Au2—Cl5	90.35 (2)
H12B—C12—H12C	109.5	Cl4^ii^—Au2—Cl5^ii^	90.34 (2)
C1—C13—H13A	109.5	Cl4—Au2—Cl5^ii^	89.66 (2)
C1—C13—H13B	109.5	Cl5—Au2—Cl5^ii^	180.0
H13A—C13—H13B	109.5		
			
C2—P1—S1—Cl1	41.13 (8)	C3—P1—C2—C21	169.11 (16)
C3—P1—S1—Cl1	−76.73 (7)	C1—P1—C2—C21	−65.87 (19)
C1—P1—S1—Cl1	164.48 (7)	S1—P1—C2—C21	49.04 (17)
C2—P1—C1—C11	−87.34 (16)	C3—P1—C2—C22	−61.10 (17)
C3—P1—C1—C11	35.47 (17)	C1—P1—C2—C22	63.92 (18)
S1—P1—C1—C11	153.80 (13)	S1—P1—C2—C22	178.83 (13)
C2—P1—C1—C13	152.00 (14)	C2—P1—C3—C31	−170.50 (15)
C3—P1—C1—C13	−85.19 (16)	C1—P1—C3—C31	61.82 (18)
S1—P1—C1—C13	33.14 (15)	S1—P1—C3—C31	−51.31 (17)
C2—P1—C1—C12	32.80 (18)	C2—P1—C3—C32	−44.51 (17)
C3—P1—C1—C12	155.61 (14)	C1—P1—C3—C32	−172.19 (14)
S1—P1—C1—C12	−86.06 (14)	S1—P1—C3—C32	74.68 (15)

Symmetry codes: (i) −*x*, −*y*+1, −*z*+1; (ii) −*x*+1, −*y*+1, −*z*.

### (tert-Butyl)(chlorosulfanyl)bis(propan-2-yl)phosphonium tetrachloridoaurate(III) (18a) Hydrogen-bond geometry (Å, º)

**Table:**

*D*—H···*A*	*D*—H	H···*A*	*D*···*A*	*D*—H···*A*
C13—H13*B*···S1	0.98	2.63	3.073 (2)	107
C2—H2···Cl1	1.00	2.95	3.383 (2)	107
C32—H32*C*···Cl1	0.98	2.90	3.635 (2)	133
C21—H21*C*···Cl3	0.98	2.93	3.705 (2)	137
C11—H11*A*···Cl5	0.98	2.97	3.742 (2)	136
C3—H3···Au2	1.00	2.75	3.693 (2)	158
C2—H2···Cl2^i^	1.00	2.93	3.688 (2)	133
C22—H22*A*···Cl3^iii^	0.98	2.82	3.642 (2)	142
C12—H12*B*···Cl3^iv^	0.98	2.91	3.754 (2)	145
C13—H13*B*···Cl3^iv^	0.98	2.98	3.876 (2)	153
C12—H12*A*···Cl4^v^	0.98	2.78	3.699 (2)	157
C13—H13*A*···Cl4^vi^	0.98	2.93	3.760 (2)	143

Symmetry codes: (i) −*x*, −*y*+1, −*z*+1; (iii) −*x*+1, −*y*+1, −*z*+1; (iv) −*x*+1, −*y*, −*z*+1; (v) *x*+1, *y*−1, *z*; (vi) *x*, *y*−1, *z*.

### Bis(tert-butyl)(chlorosulfanyl)(propan-2-yl)phosphonium tetrachloridoaurate(III) (19a) Crystal data

**Table:**

(C_11_H_25_ClPS)[AuCl_4_]	*F*(000) = 2288
*M**_r_* = 594.55	*D*_x_ = 2.031 Mg m^−^^3^
Monoclinic, *C*2/*c*	Mo *K*α radiation, λ = 0.71073 Å
*a* = 33.653 (3) Å	Cell parameters from 9326 reflections
*b* = 7.7933 (3) Å	θ = 2.3–29.3°
*c* = 16.212 (2) Å	µ = 8.43 mm^−^^1^
β = 113.861 (8)°	*T* = 100 K
*V* = 3888.5 (6) Å^3^	Plate, yellow
*Z* = 8	0.2 × 0.02 × 0.01 mm

### Bis(tert-butyl)(chlorosulfanyl)(propan-2-yl)phosphonium tetrachloridoaurate(III) (19a) Data collection

**Table:**

Oxford Diffraction Xcalibur, Eos diffractometer	4815 independent reflections
Radiation source: Enhance (Mo) X-ray Source	3861 reflections with *I* > 2σ(*I*)
Detector resolution: 16.1419 pixels mm^-1^	*R*_int_ = 0.096
ω scan	θ_max_ = 28.3°, θ_min_ = 2.5°
Absorption correction: multi-scan (CrysAlisPro; Rigaku OD, 2015)	*h* = −44→44
*T*_min_ = 0.698, *T*_max_ = 1.000	*k* = −10→10
84613 measured reflections	*l* = −21→21

### Bis(tert-butyl)(chlorosulfanyl)(propan-2-yl)phosphonium tetrachloridoaurate(III) (19a) Refinement

**Table:**

Refinement on *F*^2^	Primary atom site location: structure-invariant direct methods
Least-squares matrix: full	Secondary atom site location: difference Fourier map
*R*[*F*^2^ > 2σ(*F*^2^)] = 0.033	Hydrogen site location: inferred from neighbouring sites
*wR*(*F*^2^) = 0.064	H-atom parameters constrained
*S* = 1.04	*w* = 1/[σ^2^(*F*_o_^2^) + (0.0233*P*)^2^ + 18.2209*P*] where *P* = (*F*_o_^2^ + 2*F*_c_^2^)/3
4815 reflections	(Δ/σ)_max_ = 0.001
191 parameters	Δρ_max_ = 2.42 e Å^−^^3^
1 restraint	Δρ_min_ = −1.86 e Å^−^^3^

### Bis(tert-butyl)(chlorosulfanyl)(propan-2-yl)phosphonium tetrachloridoaurate(III) (19a) Special details

**Table:**

Refinement. The anion centred on Au1 is disordered, with the atoms Cl3 and Cl4 being slightly displaced from the twofold axis. Dimensions of disordered groups should be interpreted with caution.

### Bis(tert-butyl)(chlorosulfanyl)(propan-2-yl)phosphonium tetrachloridoaurate(III) (19a) Fractional atomic coordinates and isotropic or equivalent isotropic displacement parameters (Å^2^)

**Table:**

	*x*	*y*	*z*	*U*_iso_*/*U*_eq_	Occ. (<1)
P1	0.37573 (4)	0.61321 (15)	0.40339 (8)	0.0139 (2)	
S1	0.42233 (4)	0.76384 (17)	0.50388 (8)	0.0229 (3)	
Cl1	0.38636 (5)	0.89460 (17)	0.55781 (9)	0.0318 (3)	
C1	0.41258 (15)	0.4470 (6)	0.3892 (3)	0.0178 (10)	
C2	0.34680 (15)	0.7536 (6)	0.3034 (3)	0.0168 (9)	
C3	0.33665 (15)	0.5286 (6)	0.4457 (3)	0.0181 (10)	
H3	0.315890	0.624366	0.439720	0.022*	
C23	0.33158 (18)	0.9160 (6)	0.3367 (3)	0.0261 (12)	
H23A	0.315036	0.989329	0.285100	0.039*	
H23B	0.356937	0.978978	0.378526	0.039*	
H23C	0.313093	0.882820	0.367785	0.039*	
C21	0.30734 (16)	0.6650 (6)	0.2324 (3)	0.0224 (11)	
H21A	0.295630	0.736194	0.177973	0.034*	
H21B	0.285044	0.648821	0.256126	0.034*	
H21C	0.316006	0.553128	0.217523	0.034*	
C22	0.37784 (16)	0.8113 (6)	0.2609 (3)	0.0225 (11)	
H22A	0.385999	0.712175	0.233957	0.034*	
H22B	0.403976	0.861654	0.307501	0.034*	
H22C	0.363397	0.897101	0.214100	0.034*	
C11	0.42557 (16)	0.3207 (6)	0.4683 (3)	0.0228 (11)	
H11A	0.446432	0.237487	0.463790	0.034*	
H11B	0.399687	0.260225	0.466370	0.034*	
H11C	0.438904	0.383796	0.525243	0.034*	
C12	0.38961 (16)	0.3496 (6)	0.3002 (3)	0.0207 (10)	
H12A	0.408965	0.260339	0.294951	0.031*	
H12B	0.382171	0.429777	0.249589	0.031*	
H12C	0.363031	0.296526	0.299250	0.031*	
C13	0.45356 (16)	0.5339 (7)	0.3899 (3)	0.0247 (11)	
H13A	0.467984	0.597214	0.446518	0.037*	
H13B	0.445536	0.613540	0.338974	0.037*	
H13C	0.473373	0.446495	0.384784	0.037*	
C32	0.30897 (16)	0.3797 (6)	0.3897 (3)	0.0211 (11)	
H32A	0.326886	0.276078	0.400758	0.032*	
H32B	0.297679	0.409135	0.325450	0.032*	
H32C	0.284660	0.358817	0.406976	0.032*	
C31	0.35594 (17)	0.4771 (7)	0.5463 (3)	0.0246 (11)	
H31A	0.332771	0.474437	0.568312	0.037*	
H31B	0.378026	0.561094	0.581252	0.037*	
H31C	0.369270	0.363282	0.553266	0.037*	
Au1	0.500000	0.99691 (3)	0.750000	0.01611 (7)	
Cl2	0.51000 (4)	0.99765 (15)	0.61880 (7)	0.0205 (2)	
Cl3	0.49041 (17)	1.2856 (3)	0.7334 (3)	0.0361 (13)	0.5
Cl4	0.5066 (3)	0.7069 (3)	0.7565 (13)	0.0271 (18)	0.5
Au2	0.250000	0.750000	0.500000	0.01468 (7)	
Cl5	0.27160 (4)	1.02504 (15)	0.49346 (8)	0.0248 (3)	
Cl6	0.22544 (4)	0.72530 (15)	0.34763 (7)	0.0241 (3)	

### Bis(tert-butyl)(chlorosulfanyl)(propan-2-yl)phosphonium tetrachloridoaurate(III) (19a) Atomic displacement parameters (Å^2^)

**Table:**

	*U*^11^	*U*^22^	*U*^33^	*U*^12^	*U*^13^	*U*^23^
P1	0.0161 (6)	0.0138 (6)	0.0118 (5)	−0.0003 (5)	0.0056 (5)	−0.0005 (4)
S1	0.0230 (6)	0.0237 (7)	0.0186 (6)	−0.0052 (5)	0.0049 (5)	−0.0082 (5)
Cl1	0.0389 (8)	0.0307 (8)	0.0254 (7)	0.0011 (6)	0.0125 (6)	−0.0113 (5)
C1	0.020 (2)	0.022 (3)	0.013 (2)	0.0033 (19)	0.0081 (19)	0.0007 (19)
C2	0.023 (2)	0.012 (2)	0.014 (2)	−0.002 (2)	0.0067 (19)	0.0029 (18)
C3	0.021 (2)	0.017 (3)	0.017 (2)	0.0041 (19)	0.0087 (19)	0.0034 (18)
C23	0.033 (3)	0.020 (3)	0.022 (3)	0.003 (2)	0.008 (2)	0.001 (2)
C21	0.025 (3)	0.021 (3)	0.017 (2)	−0.001 (2)	0.006 (2)	0.000 (2)
C22	0.025 (3)	0.020 (3)	0.023 (3)	−0.003 (2)	0.012 (2)	0.005 (2)
C11	0.023 (3)	0.021 (3)	0.023 (3)	0.008 (2)	0.008 (2)	0.008 (2)
C12	0.024 (3)	0.015 (2)	0.025 (3)	0.002 (2)	0.012 (2)	−0.003 (2)
C13	0.023 (3)	0.028 (3)	0.025 (3)	0.003 (2)	0.012 (2)	0.003 (2)
C32	0.030 (3)	0.016 (3)	0.022 (2)	−0.007 (2)	0.015 (2)	−0.001 (2)
C31	0.030 (3)	0.029 (3)	0.018 (2)	−0.002 (2)	0.013 (2)	0.003 (2)
Au1	0.01937 (13)	0.01138 (13)	0.02047 (13)	0.000	0.01105 (10)	0.000
Cl2	0.0246 (6)	0.0207 (6)	0.0193 (5)	−0.0025 (5)	0.0119 (5)	−0.0009 (5)
Cl3	0.079 (5)	0.0095 (10)	0.039 (4)	0.0076 (13)	0.044 (3)	0.0037 (11)
Cl4	0.047 (6)	0.0117 (9)	0.028 (5)	0.0041 (14)	0.021 (5)	−0.0002 (16)
Au2	0.01788 (12)	0.01255 (13)	0.01355 (12)	0.00187 (10)	0.00630 (9)	−0.00001 (10)
Cl5	0.0353 (7)	0.0140 (6)	0.0274 (6)	−0.0033 (5)	0.0149 (5)	−0.0009 (5)
Cl6	0.0342 (7)	0.0222 (6)	0.0128 (5)	0.0050 (5)	0.0062 (5)	0.0001 (5)

### Bis(tert-butyl)(chlorosulfanyl)(propan-2-yl)phosphonium tetrachloridoaurate(III) (19a) Geometric parameters (Å, º)

**Table:**

P1—C3	1.834 (5)	C3—C31	1.545 (6)
P1—C2	1.870 (4)	Au1—Cl4^i^	2.269 (2)
P1—C1	1.871 (5)	Au1—Cl4	2.269 (2)
P1—S1	2.1035 (16)	Au1—Cl3^i^	2.274 (2)
S1—Cl1	2.0310 (19)	Au1—Cl3	2.274 (2)
S1—Cl2	3.3240 (17)	Au1—Cl2^i^	2.2839 (11)
C1—C13	1.532 (7)	Au1—Cl2	2.2839 (11)
C1—C11	1.533 (6)	Cl3—Cl3^i^	0.653 (8)
C1—C12	1.534 (6)	Cl4—Cl4^i^	0.409 (18)
C2—C21	1.526 (6)	Au2—Cl6	2.2738 (11)
C2—C22	1.533 (6)	Au2—Cl6^ii^	2.2738 (11)
C2—C23	1.543 (7)	Au2—Cl5^ii^	2.2796 (12)
C3—C32	1.534 (6)	Au2—Cl5	2.2796 (12)
			
C3—P1—C2	109.5 (2)	Cl4^i^—Au1—Cl4	10.3 (5)
C3—P1—C1	114.1 (2)	Cl4^i^—Au1—Cl3^i^	176.1 (4)
C2—P1—C1	115.9 (2)	Cl4—Au1—Cl3^i^	166.8 (3)
C3—P1—S1	109.51 (16)	Cl4^i^—Au1—Cl3	166.8 (3)
C2—P1—S1	107.97 (16)	Cl4—Au1—Cl3	176.1 (4)
C1—P1—S1	99.09 (16)	Cl3^i^—Au1—Cl3	16.50 (19)
Cl1—S1—P1	103.06 (7)	Cl4^i^—Au1—Cl2^i^	89.8 (5)
Cl1—S1—Cl2	91.84 (6)	Cl4—Au1—Cl2^i^	90.5 (5)
P1—S1—Cl2	162.69 (7)	Cl3^i^—Au1—Cl2^i^	87.38 (17)
C13—C1—C11	109.0 (4)	Cl3—Au1—Cl2^i^	92.33 (17)
C13—C1—C12	109.6 (4)	Cl4^i^—Au1—Cl2	90.5 (5)
C11—C1—C12	109.5 (4)	Cl4—Au1—Cl2	89.8 (5)
C13—C1—P1	109.4 (3)	Cl3^i^—Au1—Cl2	92.33 (17)
C11—C1—P1	108.7 (3)	Cl3—Au1—Cl2	87.38 (17)
C12—C1—P1	110.6 (3)	Cl2^i^—Au1—Cl2	179.71 (6)
C21—C2—C22	109.7 (4)	Au1—Cl2—S1	95.10 (4)
C21—C2—C23	108.9 (4)	Cl3^i^—Cl3—Au1	81.75 (9)
C22—C2—C23	107.4 (4)	Cl4^i^—Cl4—Au1	84.8 (2)
C21—C2—P1	112.2 (3)	Cl6—Au2—Cl6^ii^	180.00 (6)
C22—C2—P1	110.5 (3)	Cl6—Au2—Cl5^ii^	89.33 (4)
C23—C2—P1	107.8 (3)	Cl6^ii^—Au2—Cl5^ii^	90.67 (4)
C32—C3—C31	108.9 (4)	Cl6—Au2—Cl5	90.67 (4)
C32—C3—P1	114.0 (3)	Cl6^ii^—Au2—Cl5	89.33 (4)
C31—C3—P1	115.4 (3)	Cl5^ii^—Au2—Cl5	180.0
			
C3—P1—S1—Cl1	−40.49 (18)	C3—P1—C2—C21	−51.4 (4)
C2—P1—S1—Cl1	78.68 (17)	C1—P1—C2—C21	79.4 (4)
C1—P1—S1—Cl1	−160.17 (16)	S1—P1—C2—C21	−170.6 (3)
C3—P1—S1—Cl2	170.8 (3)	C3—P1—C2—C22	−174.3 (3)
C2—P1—S1—Cl2	−70.1 (3)	C1—P1—C2—C22	−43.5 (4)
C1—P1—S1—Cl2	51.1 (3)	S1—P1—C2—C22	66.6 (3)
C3—P1—C1—C13	−158.2 (3)	C3—P1—C2—C23	68.5 (4)
C2—P1—C1—C13	73.2 (4)	C1—P1—C2—C23	−160.6 (3)
S1—P1—C1—C13	−42.0 (3)	S1—P1—C2—C23	−50.6 (3)
C3—P1—C1—C11	−39.3 (4)	C2—P1—C3—C32	77.6 (4)
C2—P1—C1—C11	−167.9 (3)	C1—P1—C3—C32	−54.2 (4)
S1—P1—C1—C11	76.9 (3)	S1—P1—C3—C32	−164.2 (3)
C3—P1—C1—C12	81.0 (4)	C2—P1—C3—C31	−155.2 (3)
C2—P1—C1—C12	−47.6 (4)	C1—P1—C3—C31	73.0 (4)
S1—P1—C1—C12	−162.8 (3)	S1—P1—C3—C31	−37.0 (4)

Symmetry codes: (i) −*x*+1, *y*, −*z*+3/2; (ii) −*x*+1/2, −*y*+3/2, −*z*+1.

### Bis(tert-butyl)(chlorosulfanyl)(propan-2-yl)phosphonium tetrachloridoaurate(III) (19a) Hydrogen-bond geometry (Å, º)

**Table:**

*D*—H···*A*	*D*—H	H···*A*	*D*···*A*	*D*—H···*A*
C3—H3···Au2	1.00	2.93	3.786 (5)	144
C3—H3···Cl6	1.00	2.90	3.751 (5)	143
C13—H13*A*···S1	0.98	2.46	3.049 (5)	118
C23—H23*B*···Cl1	0.98	2.74	3.305 (5)	117
C31—H31*B*···Cl1	0.98	2.66	3.393 (5)	132
C23—H23*A*···Cl6^iii^	0.98	2.74	3.713 (5)	173
C21—H21*C*···Cl6^iv^	0.98	2.90	3.672 (5)	137
C13—H13*B*···Cl3^v^	0.98	2.81	3.536 (8)	131
C22—H22*B*···Cl2^vi^	0.98	2.87	3.783 (5)	156
C13—H13*C*···Cl4^vii^	0.98	2.89	3.677 (19)	138

Symmetry codes: (iii) −*x*+1/2, *y*+1/2, −*z*+1/2; (iv) −*x*+1/2, *y*−1/2, −*z*+1/2; (v) *x*, −*y*+2, *z*−1/2; (vi) −*x*+1, −*y*+2, −*z*+1; (vii) −*x*+1, −*y*+1, −*z*+1.

### Tris(tert-butyl)(chlorosulfanyl)phosphonium tetrachloridoaurate(III) (20a) Crystal data

**Table:**

(C_12_H_27_ClPS)[AuCl_4_]	*F*(000) = 1176
*M**_r_* = 608.58	*D*_x_ = 1.997 Mg m^−^^3^
Monoclinic, *P*2_1_/*n*	Mo *K*α radiation, λ = 0.71073 Å
*a* = 9.7661 (3) Å	Cell parameters from 16785 reflections
*b* = 12.5787 (3) Å	θ = 2.1–30.9°
*c* = 16.9776 (5) Å	µ = 8.10 mm^−^^1^
β = 103.887 (3)°	*T* = 100 K
*V* = 2024.67 (10) Å^3^	Block, yellow
*Z* = 4	0.15 × 0.07 × 0.04 mm

### Tris(tert-butyl)(chlorosulfanyl)phosphonium tetrachloridoaurate(III) (20a) Data collection

**Table:**

Oxford Diffraction Xcalibur, Eos diffractometer	8891 independent reflections
Radiation source: Enhance (Mo) X-ray Source	4196 reflections with *I* > 2σ(*I*)
Detector resolution: 16.1419 pixels mm^-1^	*R*_int_ = 0.0000
ω scan	θ_max_ = 31.3°, θ_min_ = 2.2°
Absorption correction: multi-scan (CrysAlisPro; Rigaku OD, 2015)	*h* = −14→14
*T*_min_ = 0.756, *T*_max_ = 1.000	*k* = −17→18
8891 measured reflections	*l* = −24→24

### Tris(tert-butyl)(chlorosulfanyl)phosphonium tetrachloridoaurate(III) (20a) Refinement

**Table:**

Refinement on *F*^2^	Primary atom site location: structure-invariant direct methods
Least-squares matrix: full	Secondary atom site location: difference Fourier map
*R*[*F*^2^ > 2σ(*F*^2^)] = 0.022	Hydrogen site location: inferred from neighbouring sites
*wR*(*F*^2^) = 0.045	H-atom parameters constrained
*S* = 0.77	*w* = 1/[σ^2^(*F*_o_^2^) + (0.0179*P*)^2^] where *P* = (*F*_o_^2^ + 2*F*_c_^2^)/3
8891 reflections	(Δ/σ)_max_ < 0.001
194 parameters	Δρ_max_ = 1.12 e Å^−^^3^
0 restraints	Δρ_min_ = −0.92 e Å^−^^3^

### Tris(tert-butyl)(chlorosulfanyl)phosphonium tetrachloridoaurate(III) (20a) Special details

**Table:**

Refinement. The data were affected by a small twinning component (by 180 degrees about the c axis) that was at first not detected, but which led to poor agreement of data with l = 4. Data reduction and refinement ("HKLF 5" method; Sheldrick, 2015) as a non-merohedral twin led to a BASF parameter (relative volume of the smaller twin component) of 0.0300 (3). As is often the case with "HKLF 5" refinements, several reflections were severely in error; 20 of these were removed from the dataset. Because equivalent reflections are merged during the generation of the "HKLF 5" intensity dataset, and because overlapped and non-overlapped reflections are both included in the refinement, the number of reflections should be interpreted carefully. The low GOOF value may be due to the difficulty of estimating e.s.d.s for the intensities of the small twin component.

### Tris(tert-butyl)(chlorosulfanyl)phosphonium tetrachloridoaurate(III) (20a) Fractional atomic coordinates and isotropic or equivalent isotropic displacement parameters (Å^2^)

**Table:**

	*x*	*y*	*z*	*U*_iso_*/*U*_eq_	
P1	0.49208 (10)	0.50047 (10)	0.25997 (5)	0.00909 (17)	
S1	0.28916 (10)	0.52505 (7)	0.18610 (5)	0.0156 (2)	
Cl1	0.15675 (9)	0.48996 (10)	0.25817 (5)	0.0199 (2)	
C1	0.5898 (3)	0.4946 (4)	0.17591 (18)	0.0136 (7)	
C2	0.5023 (4)	0.3710 (3)	0.3180 (2)	0.0134 (9)	
C3	0.5395 (4)	0.6193 (3)	0.3280 (2)	0.0100 (8)	
C11	0.7488 (4)	0.5137 (3)	0.2099 (2)	0.0175 (9)	
H11A	0.785639	0.462496	0.253266	0.026*	
H11B	0.763971	0.586113	0.231554	0.026*	
H11C	0.798098	0.504753	0.166349	0.026*	
C12	0.5311 (4)	0.5783 (3)	0.1101 (2)	0.0199 (10)	
H12A	0.579217	0.571551	0.065842	0.030*	
H12B	0.547250	0.649637	0.133674	0.030*	
H12C	0.429706	0.566905	0.088820	0.030*	
C13	0.5663 (4)	0.3869 (3)	0.1324 (2)	0.0195 (10)	
H13A	0.612098	0.387118	0.086949	0.029*	
H13B	0.464954	0.374476	0.111864	0.029*	
H13C	0.606958	0.330298	0.170529	0.029*	
C21	0.6553 (4)	0.3327 (3)	0.3404 (2)	0.0183 (9)	
H21A	0.661551	0.266126	0.371053	0.027*	
H21B	0.714792	0.386686	0.373639	0.027*	
H21C	0.687732	0.320647	0.290764	0.027*	
C22	0.4052 (5)	0.2874 (3)	0.2668 (2)	0.0212 (10)	
H22A	0.431884	0.277283	0.215255	0.032*	
H22B	0.307143	0.311762	0.256174	0.032*	
H22C	0.414629	0.219859	0.296432	0.032*	
C23	0.4521 (4)	0.3838 (3)	0.3972 (2)	0.0169 (9)	
H23A	0.456451	0.314866	0.424597	0.025*	
H23B	0.354722	0.409987	0.384259	0.025*	
H23C	0.513195	0.434685	0.432954	0.025*	
C31	0.6780 (4)	0.6028 (3)	0.3911 (2)	0.0167 (9)	
H31A	0.753578	0.588428	0.363620	0.025*	
H31B	0.668604	0.542377	0.425844	0.025*	
H31C	0.700781	0.667011	0.424346	0.025*	
C32	0.5508 (5)	0.7190 (3)	0.2774 (2)	0.0202 (9)	
H32A	0.569800	0.781178	0.313140	0.030*	
H32B	0.461964	0.729475	0.236676	0.030*	
H32C	0.627884	0.709811	0.250185	0.030*	
C33	0.4188 (4)	0.6433 (3)	0.3706 (2)	0.0160 (10)	
H33A	0.403029	0.580920	0.401926	0.024*	
H33B	0.332170	0.659840	0.329690	0.024*	
H33C	0.445164	0.704115	0.407159	0.024*	
Au1	0.000000	0.500000	0.500000	0.01111 (5)	
Cl2	0.03947 (11)	0.38336 (8)	0.40465 (6)	0.0223 (2)	
Cl3	0.10599 (12)	0.63217 (8)	0.44413 (6)	0.0255 (2)	
Au2	0.000000	0.500000	0.000000	0.01066 (5)	
Cl4	0.16838 (12)	0.37478 (9)	−0.00287 (6)	0.0176 (2)	
Cl5	0.15525 (12)	0.63167 (9)	−0.01076 (6)	0.0180 (2)	

### Tris(tert-butyl)(chlorosulfanyl)phosphonium tetrachloridoaurate(III) (20a) Atomic displacement parameters (Å^2^)

**Table:**

	*U*^11^	*U*^22^	*U*^33^	*U*^12^	*U*^13^	*U*^23^
P1	0.0088 (4)	0.0094 (3)	0.0096 (4)	0.0006 (6)	0.0030 (3)	0.0004 (6)
S1	0.0098 (4)	0.0252 (7)	0.0116 (4)	0.0007 (4)	0.0022 (4)	−0.0006 (4)
Cl1	0.0124 (4)	0.0251 (6)	0.0240 (4)	−0.0027 (5)	0.0078 (4)	−0.0028 (5)
C1	0.0126 (16)	0.0211 (19)	0.0106 (14)	0.005 (2)	0.0101 (13)	0.004 (2)
C2	0.012 (2)	0.010 (2)	0.017 (2)	−0.0012 (17)	0.0018 (17)	0.0000 (17)
C3	0.009 (2)	0.0081 (19)	0.0119 (18)	−0.0001 (16)	0.0012 (16)	−0.0002 (16)
C11	0.0168 (19)	0.018 (3)	0.0202 (17)	0.0020 (19)	0.0096 (15)	0.0055 (18)
C12	0.017 (2)	0.031 (3)	0.015 (2)	0.004 (2)	0.0108 (18)	0.0069 (18)
C13	0.018 (2)	0.031 (3)	0.012 (2)	0.002 (2)	0.0091 (18)	−0.0053 (19)
C21	0.024 (2)	0.014 (2)	0.018 (2)	0.0067 (18)	0.0077 (18)	0.0079 (16)
C22	0.028 (3)	0.012 (2)	0.027 (2)	−0.0036 (18)	0.013 (2)	−0.0003 (17)
C23	0.021 (2)	0.011 (2)	0.021 (2)	−0.0015 (19)	0.0092 (19)	0.0065 (18)
C31	0.016 (2)	0.014 (2)	0.0175 (19)	−0.0004 (17)	−0.0005 (17)	−0.0055 (16)
C32	0.021 (2)	0.015 (2)	0.024 (2)	−0.0022 (18)	0.0067 (18)	−0.0010 (17)
C33	0.019 (2)	0.011 (2)	0.017 (2)	0.0030 (18)	0.0028 (18)	−0.0021 (16)
Au1	0.00871 (10)	0.01398 (11)	0.01084 (10)	−0.00008 (14)	0.00275 (8)	0.00063 (13)
Cl2	0.0241 (6)	0.0240 (6)	0.0223 (5)	−0.0051 (5)	0.0126 (5)	−0.0084 (4)
Cl3	0.0330 (6)	0.0200 (6)	0.0291 (6)	−0.0072 (5)	0.0183 (5)	0.0010 (5)
Au2	0.01168 (10)	0.01105 (10)	0.00926 (9)	0.00079 (14)	0.00256 (8)	0.00012 (13)
Cl4	0.0163 (6)	0.0156 (6)	0.0217 (5)	0.0024 (5)	0.0060 (5)	−0.0026 (5)
Cl5	0.0161 (6)	0.0156 (6)	0.0227 (5)	−0.0002 (5)	0.0054 (5)	0.0040 (5)

### Tris(tert-butyl)(chlorosulfanyl)phosphonium tetrachloridoaurate(III) (20a) Geometric parameters (Å, º)

**Table:**

P1—C3	1.877 (4)	C21—H21B	0.9800
P1—C2	1.894 (4)	C21—H21C	0.9800
P1—C1	1.899 (3)	C22—H22A	0.9800
P1—S1	2.0983 (13)	C22—H22B	0.9800
S1—Cl1	2.0307 (13)	C22—H22C	0.9800
Cl1—Cl2	3.2652 (14)	C23—H23A	0.9800
C1—C13	1.534 (6)	C23—H23B	0.9800
C1—C11	1.541 (5)	C23—H23C	0.9800
C1—C12	1.543 (5)	C31—H31A	0.9800
C2—C21	1.529 (5)	C31—H31B	0.9800
C2—C22	1.538 (5)	C31—H31C	0.9800
C2—C23	1.546 (5)	C32—H32A	0.9800
C3—C31	1.525 (5)	C32—H32B	0.9800
C3—C32	1.539 (5)	C32—H32C	0.9800
C3—C33	1.553 (5)	C33—H33A	0.9800
C11—H11A	0.9800	C33—H33B	0.9800
C11—H11B	0.9800	C33—H33C	0.9800
C11—H11C	0.9800	Au1—Cl3	2.2818 (10)
C12—H12A	0.9800	Au1—Cl3^i^	2.2818 (10)
C12—H12B	0.9800	Au1—Cl2^i^	2.2850 (10)
C12—H12C	0.9800	Au1—Cl2	2.2850 (10)
C13—H13A	0.9800	Au2—Cl5	2.2821 (11)
C13—H13B	0.9800	Au2—Cl5^ii^	2.2822 (11)
C13—H13C	0.9800	Au2—Cl4^ii^	2.2859 (11)
C21—H21A	0.9800	Au2—Cl4	2.2860 (11)
			
C3—P1—C2	112.97 (15)	C2—C21—H21C	109.5
C3—P1—C1	113.5 (2)	H21A—C21—H21C	109.5
C2—P1—C1	112.6 (2)	H21B—C21—H21C	109.5
C3—P1—S1	107.96 (13)	C2—C22—H22A	109.5
C2—P1—S1	111.23 (13)	C2—C22—H22B	109.5
C1—P1—S1	97.43 (11)	H22A—C22—H22B	109.5
Cl1—S1—P1	104.74 (5)	C2—C22—H22C	109.5
S1—Cl1—Cl2	159.83 (6)	H22A—C22—H22C	109.5
C13—C1—C11	109.8 (3)	H22B—C22—H22C	109.5
C13—C1—C12	105.5 (3)	C2—C23—H23A	109.5
C11—C1—C12	109.3 (3)	C2—C23—H23B	109.5
C13—C1—P1	110.7 (3)	H23A—C23—H23B	109.5
C11—C1—P1	110.9 (2)	C2—C23—H23C	109.5
C12—C1—P1	110.6 (3)	H23A—C23—H23C	109.5
C21—C2—C22	111.0 (3)	H23B—C23—H23C	109.5
C21—C2—C23	108.2 (3)	C3—C31—H31A	109.5
C22—C2—C23	106.3 (3)	C3—C31—H31B	109.5
C21—C2—P1	109.1 (3)	H31A—C31—H31B	109.5
C22—C2—P1	110.2 (3)	C3—C31—H31C	109.5
C23—C2—P1	112.0 (3)	H31A—C31—H31C	109.5
C31—C3—C32	109.4 (3)	H31B—C31—H31C	109.5
C31—C3—C33	110.1 (3)	C3—C32—H32A	109.5
C32—C3—C33	105.4 (3)	C3—C32—H32B	109.5
C31—C3—P1	111.7 (3)	H32A—C32—H32B	109.5
C32—C3—P1	110.2 (3)	C3—C32—H32C	109.5
C33—C3—P1	109.8 (3)	H32A—C32—H32C	109.5
C1—C11—H11A	109.5	H32B—C32—H32C	109.5
C1—C11—H11B	109.5	C3—C33—H33A	109.5
H11A—C11—H11B	109.5	C3—C33—H33B	109.5
C1—C11—H11C	109.5	H33A—C33—H33B	109.5
H11A—C11—H11C	109.5	C3—C33—H33C	109.5
H11B—C11—H11C	109.5	H33A—C33—H33C	109.5
C1—C12—H12A	109.5	H33B—C33—H33C	109.5
C1—C12—H12B	109.5	Cl3—Au1—Cl3^i^	180.0
H12A—C12—H12B	109.5	Cl3—Au1—Cl2^i^	89.84 (4)
C1—C12—H12C	109.5	Cl3^i^—Au1—Cl2^i^	90.16 (4)
H12A—C12—H12C	109.5	Cl3—Au1—Cl2	90.16 (4)
H12B—C12—H12C	109.5	Cl3^i^—Au1—Cl2	89.84 (4)
C1—C13—H13A	109.5	Cl2^i^—Au1—Cl2	180.0
C1—C13—H13B	109.5	Au1—Cl2—Cl1	115.19 (4)
H13A—C13—H13B	109.5	Cl5—Au2—Cl5^ii^	180.0
C1—C13—H13C	109.5	Cl5—Au2—Cl4^ii^	89.82 (3)
H13A—C13—H13C	109.5	Cl5^ii^—Au2—Cl4^ii^	90.18 (3)
H13B—C13—H13C	109.5	Cl5—Au2—Cl4	90.18 (3)
C2—C21—H21A	109.5	Cl5^ii^—Au2—Cl4	89.82 (3)
C2—C21—H21B	109.5	Cl4^ii^—Au2—Cl4	180.0
H21A—C21—H21B	109.5		
			
C3—P1—S1—Cl1	−78.89 (15)	C3—P1—C2—C22	155.2 (3)
C2—P1—S1—Cl1	45.59 (15)	C1—P1—C2—C22	−74.5 (3)
C1—P1—S1—Cl1	163.38 (16)	S1—P1—C2—C22	33.6 (3)
P1—S1—Cl1—Cl2	−25.47 (19)	C3—P1—C2—C23	37.1 (3)
C3—P1—C1—C13	170.0 (2)	C1—P1—C2—C23	167.3 (3)
C2—P1—C1—C13	40.0 (3)	S1—P1—C2—C23	−84.5 (3)
S1—P1—C1—C13	−76.7 (3)	C2—P1—C3—C31	49.6 (3)
C3—P1—C1—C11	47.9 (4)	C1—P1—C3—C31	−80.1 (3)
C2—P1—C1—C11	−82.0 (3)	S1—P1—C3—C31	173.0 (2)
S1—P1—C1—C11	161.2 (3)	C2—P1—C3—C32	171.5 (3)
C3—P1—C1—C12	−73.4 (3)	C1—P1—C3—C32	41.7 (3)
C2—P1—C1—C12	156.6 (3)	S1—P1—C3—C32	−65.1 (3)
S1—P1—C1—C12	39.9 (3)	C2—P1—C3—C33	−72.8 (3)
C3—P1—C2—C21	−82.7 (3)	C1—P1—C3—C33	157.5 (2)
C1—P1—C2—C21	47.6 (3)	S1—P1—C3—C33	50.6 (3)
S1—P1—C2—C21	155.8 (2)		

Symmetry codes: (i) −*x*, −*y*+1, −*z*+1; (ii) −*x*, −*y*+1, −*z*.

### Tris(tert-butyl)(chlorosulfanyl)phosphonium tetrachloridoaurate(III) (20a) Hydrogen-bond geometry (Å, º)

**Table:**

*D*—H···*A*	*D*—H	H···*A*	*D*···*A*	*D*—H···*A*
C23—H23*B*···Cl1	0.98	2.71	3.522 (4)	141
C22—H22*B*···Cl1	0.98	2.68	3.497 (4)	141
C33—H33*B*···Cl1	0.98	2.82	3.401 (4)	118
C13—H13*A*···Cl5^iii^	0.98	2.88	3.796 (4)	156
C12—H12*C*···Cl5	0.98	2.92	3.810 (4)	151
C31—H31*C*···Cl5^iv^	0.98	2.84	3.763 (4)	158
C12—H12*C*···S1	0.98	2.44	3.027 (4)	118
C22—H22*B*···S1	0.98	2.92	3.370 (4)	109
C33—H33*B*···S1	0.98	2.92	3.424 (4)	113
C21—H21*A*···Cl4^v^	0.98	2.77	3.708 (4)	161

Symmetry codes: (iii) −*x*+1, −*y*+1, −*z*; (iv) *x*+1/2, −*y*+3/2, *z*+1/2; (v) *x*+1/2, −*y*+1/2, *z*+1/2.

### (Chloroselanyl)tris(propan-2-yl)phosphonium tetrachloridoaurate(III) (21a) Crystal data

**Table:**

(C_9_H_21_ClPSe)[AuCl_4_]	*Z* = 2
*M**_r_* = 613.40	*F*(000) = 576
Triclinic, *P*1	*D*_x_ = 2.280 Mg m^−^^3^
*a* = 7.4202 (3) Å	Mo *K*α radiation, λ = 0.71073 Å
*b* = 8.5966 (3) Å	Cell parameters from 42603 reflections
*c* = 15.3980 (6) Å	θ = 2.6–30.8°
α = 97.497 (3)°	µ = 11.09 mm^−^^1^
β = 100.128 (4)°	*T* = 100 K
γ = 109.324 (4)°	Block, yellow
*V* = 893.42 (6) Å^3^	0.18 × 0.15 × 0.10 mm

### (Chloroselanyl)tris(propan-2-yl)phosphonium tetrachloridoaurate(III) (21a) Data collection

**Table:**

Oxford Diffraction Xcalibur, Eos diffractometer	5322 independent reflections
Radiation source: Enhance (Mo) X-ray Source	4900 reflections with *I* > 2σ(*I*)
Detector resolution: 16.1419 pixels mm^-1^	*R*_int_ = 0.039
ω scan	θ_max_ = 30.9°, θ_min_ = 2.6°
Absorption correction: multi-scan (CrysAlisPro; Rigaku OD, 2015)	*h* = −10→10
*T*_min_ = 0.631, *T*_max_ = 1.000	*k* = −12→12
106851 measured reflections	*l* = −22→22

### (Chloroselanyl)tris(propan-2-yl)phosphonium tetrachloridoaurate(III) (21a) Refinement

**Table:**

Refinement on *F*^2^	Secondary atom site location: difference Fourier map
Least-squares matrix: full	Hydrogen site location: inferred from neighbouring sites
*R*[*F*^2^ > 2σ(*F*^2^)] = 0.016	H-atom parameters constrained
*wR*(*F*^2^) = 0.033	*w* = 1/[σ^2^(*F*_o_^2^) + (0.014*P*)^2^ + 0.5143*P*] where *P* = (*F*_o_^2^ + 2*F*_c_^2^)/3
*S* = 1.10	(Δ/σ)_max_ = 0.001
5322 reflections	Δρ_max_ = 0.83 e Å^−^^3^
164 parameters	Δρ_min_ = −0.85 e Å^−^^3^
0 restraints	Extinction correction: *SHELXL2019/3* (Sheldrick, 2015), *F*_c_^*^ = *kF*_c_[1 + 0.001 *F*_c_^2^λ^3^/sin(2θ)]^-1/4^
Primary atom site location: structure-invariant direct methods	Extinction coefficient: 0.00346 (11)

### (Chloroselanyl)tris(propan-2-yl)phosphonium tetrachloridoaurate(III) (21a) Fractional atomic coordinates and isotropic or equivalent isotropic displacement parameters (Å^2^)

**Table:**

	*x*	*y*	*z*	*U*_iso_*/*U*_eq_	
P1	0.53786 (6)	0.48435 (6)	0.25613 (3)	0.01070 (9)	
Se1	0.73544 (3)	0.38369 (2)	0.34073 (2)	0.01473 (4)	
Cl1	0.71221 (7)	0.17157 (6)	0.23989 (3)	0.02085 (10)	
C1	0.4802 (3)	0.6161 (2)	0.34217 (12)	0.0148 (4)	
H1	0.605803	0.679304	0.388314	0.018*	
C2	0.3309 (3)	0.3099 (2)	0.18196 (12)	0.0147 (4)	
H2	0.384866	0.259066	0.135410	0.018*	
C3	0.6750 (3)	0.6170 (2)	0.18854 (12)	0.0143 (3)	
H3	0.589658	0.673188	0.159062	0.017*	
C11	0.4107 (3)	0.7490 (3)	0.30424 (14)	0.0233 (4)	
H11A	0.287805	0.692478	0.258152	0.035*	
H11B	0.511169	0.818179	0.277363	0.035*	
H11C	0.388871	0.820785	0.353057	0.035*	
C12	0.3354 (3)	0.5142 (3)	0.39157 (14)	0.0216 (4)	
H12A	0.331514	0.588542	0.444612	0.032*	
H12B	0.377732	0.424896	0.410608	0.032*	
H12C	0.204198	0.463957	0.351138	0.032*	
C21	0.2291 (3)	0.1679 (3)	0.22723 (14)	0.0219 (4)	
H21A	0.160227	0.208475	0.268474	0.033*	
H21B	0.327474	0.131685	0.261082	0.033*	
H21C	0.134425	0.072642	0.181119	0.033*	
C22	0.1837 (3)	0.3768 (3)	0.13123 (14)	0.0221 (4)	
H22A	0.082901	0.284694	0.085424	0.033*	
H22B	0.252924	0.465671	0.102071	0.033*	
H22C	0.121334	0.422612	0.173898	0.033*	
C31	0.8610 (3)	0.7560 (3)	0.24968 (14)	0.0233 (4)	
H31A	0.944035	0.704355	0.282342	0.035*	
H31B	0.823129	0.826372	0.292886	0.035*	
H31C	0.934017	0.825763	0.212675	0.035*	
C32	0.7241 (3)	0.5134 (3)	0.11392 (14)	0.0210 (4)	
H32A	0.803099	0.589069	0.081055	0.032*	
H32B	0.602206	0.435669	0.072279	0.032*	
H32C	0.798569	0.449167	0.140630	0.032*	
Au1	0.500000	0.000000	0.000000	0.01487 (3)	
Cl3	0.30365 (8)	−0.18377 (7)	0.06920 (3)	0.02345 (10)	
Cl2	0.75097 (7)	−0.08933 (7)	0.04630 (3)	0.02262 (10)	
Au2	1.000000	1.000000	0.500000	0.01214 (3)	
Cl4	1.29351 (7)	1.06893 (7)	0.45972 (3)	0.02286 (10)	
Cl5	0.99249 (7)	0.73205 (6)	0.49898 (3)	0.02014 (10)	

### (Chloroselanyl)tris(propan-2-yl)phosphonium tetrachloridoaurate(III) (21a) Atomic displacement parameters (Å^2^)

**Table:**

	*U*^11^	*U*^22^	*U*^33^	*U*^12^	*U*^13^	*U*^23^
P1	0.01038 (19)	0.0108 (2)	0.01082 (19)	0.00406 (17)	0.00169 (15)	0.00242 (16)
Se1	0.01665 (9)	0.01492 (10)	0.01344 (8)	0.00888 (7)	−0.00026 (6)	0.00238 (7)
Cl1	0.0250 (2)	0.0155 (2)	0.0223 (2)	0.0110 (2)	0.00174 (18)	−0.00024 (18)
C1	0.0159 (8)	0.0157 (10)	0.0131 (8)	0.0072 (7)	0.0029 (6)	0.0012 (7)
C2	0.0126 (8)	0.0147 (10)	0.0132 (8)	0.0024 (7)	0.0003 (6)	0.0010 (7)
C3	0.0130 (8)	0.0146 (10)	0.0152 (8)	0.0042 (7)	0.0036 (6)	0.0051 (7)
C11	0.0325 (11)	0.0230 (12)	0.0227 (10)	0.0198 (10)	0.0078 (8)	0.0060 (8)
C12	0.0218 (10)	0.0278 (12)	0.0203 (9)	0.0114 (9)	0.0113 (8)	0.0067 (8)
C21	0.0178 (9)	0.0176 (11)	0.0257 (10)	0.0014 (8)	0.0022 (8)	0.0061 (8)
C22	0.0158 (9)	0.0243 (12)	0.0220 (9)	0.0055 (8)	−0.0032 (7)	0.0054 (8)
C31	0.0178 (9)	0.0228 (12)	0.0224 (10)	−0.0009 (8)	0.0007 (7)	0.0084 (8)
C32	0.0244 (10)	0.0230 (12)	0.0211 (9)	0.0109 (9)	0.0121 (8)	0.0078 (8)
Au1	0.02126 (5)	0.01143 (6)	0.01034 (5)	0.00685 (4)	−0.00120 (3)	0.00097 (3)
Cl3	0.0289 (2)	0.0204 (3)	0.0213 (2)	0.0083 (2)	0.00511 (19)	0.00814 (19)
Cl2	0.0257 (2)	0.0215 (3)	0.0211 (2)	0.0125 (2)	−0.00138 (18)	0.00491 (19)
Au2	0.01478 (5)	0.01236 (6)	0.00960 (4)	0.00728 (4)	0.00026 (3)	0.00054 (3)
Cl4	0.0200 (2)	0.0224 (3)	0.0262 (2)	0.0081 (2)	0.00875 (18)	−0.00042 (19)
Cl5	0.0254 (2)	0.0148 (2)	0.0214 (2)	0.01103 (19)	0.00119 (17)	0.00335 (17)

### (Chloroselanyl)tris(propan-2-yl)phosphonium tetrachloridoaurate(III) (21a) Geometric parameters (Å, º)

**Table:**

P1—C2	1.8230 (19)	C21—H21A	0.9800
P1—C3	1.8256 (19)	C21—H21B	0.9800
P1—C1	1.8304 (18)	C21—H21C	0.9800
P1—Se1	2.2488 (5)	C22—H22A	0.9800
Se1—Cl1	2.1736 (5)	C22—H22B	0.9800
Cl1—Cl2	3.6031 (7)	C22—H22C	0.9800
C1—C12	1.529 (3)	C31—H31A	0.9800
C1—C11	1.539 (3)	C31—H31B	0.9800
C1—H1	1.0000	C31—H31C	0.9800
C2—C21	1.531 (3)	C32—H32A	0.9800
C2—C22	1.539 (3)	C32—H32B	0.9800
C2—H2	1.0000	C32—H32C	0.9800
C3—C32	1.530 (3)	Au1—Cl2^i^	2.2778 (5)
C3—C31	1.542 (3)	Au1—Cl2	2.2778 (5)
C3—H3	1.0000	Au1—Cl3	2.2838 (5)
C11—H11A	0.9800	Au1—Cl3^i^	2.2839 (5)
C11—H11B	0.9800	Au2—Cl4^ii^	2.2795 (5)
C11—H11C	0.9800	Au2—Cl4	2.2795 (5)
C12—H12A	0.9800	Au2—Cl5^ii^	2.2836 (5)
C12—H12B	0.9800	Au2—Cl5	2.2836 (5)
C12—H12C	0.9800		
			
C2—P1—C3	109.48 (8)	C2—C21—H21A	109.5
C2—P1—C1	116.55 (9)	C2—C21—H21B	109.5
C3—P1—C1	109.25 (9)	H21A—C21—H21B	109.5
C2—P1—Se1	109.69 (6)	C2—C21—H21C	109.5
C3—P1—Se1	109.75 (6)	H21A—C21—H21C	109.5
C1—P1—Se1	101.79 (6)	H21B—C21—H21C	109.5
Cl1—Se1—P1	98.381 (19)	C2—C22—H22A	109.5
Se1—Cl1—Cl2	164.28 (2)	C2—C22—H22B	109.5
C12—C1—C11	111.32 (16)	H22A—C22—H22B	109.5
C12—C1—P1	113.15 (14)	C2—C22—H22C	109.5
C11—C1—P1	112.12 (13)	H22A—C22—H22C	109.5
C12—C1—H1	106.6	H22B—C22—H22C	109.5
C11—C1—H1	106.6	C3—C31—H31A	109.5
P1—C1—H1	106.6	C3—C31—H31B	109.5
C21—C2—C22	111.54 (16)	H31A—C31—H31B	109.5
C21—C2—P1	115.24 (13)	C3—C31—H31C	109.5
C22—C2—P1	110.02 (14)	H31A—C31—H31C	109.5
C21—C2—H2	106.5	H31B—C31—H31C	109.5
C22—C2—H2	106.5	C3—C32—H32A	109.5
P1—C2—H2	106.5	C3—C32—H32B	109.5
C32—C3—C31	111.75 (16)	H32A—C32—H32B	109.5
C32—C3—P1	111.79 (14)	C3—C32—H32C	109.5
C31—C3—P1	110.18 (13)	H32A—C32—H32C	109.5
C32—C3—H3	107.6	H32B—C32—H32C	109.5
C31—C3—H3	107.6	Cl2^i^—Au1—Cl2	180.00 (3)
P1—C3—H3	107.6	Cl2^i^—Au1—Cl3	89.455 (19)
C1—C11—H11A	109.5	Cl2—Au1—Cl3	90.545 (19)
C1—C11—H11B	109.5	Cl2^i^—Au1—Cl3^i^	90.544 (19)
H11A—C11—H11B	109.5	Cl2—Au1—Cl3^i^	89.456 (19)
C1—C11—H11C	109.5	Cl3—Au1—Cl3^i^	180.0
H11A—C11—H11C	109.5	Au1—Cl2—Cl1	72.720 (15)
H11B—C11—H11C	109.5	Cl4^ii^—Au2—Cl4	180.0
C1—C12—H12A	109.5	Cl4^ii^—Au2—Cl5^ii^	89.900 (19)
C1—C12—H12B	109.5	Cl4—Au2—Cl5^ii^	90.100 (19)
H12A—C12—H12B	109.5	Cl4^ii^—Au2—Cl5	90.100 (19)
C1—C12—H12C	109.5	Cl4—Au2—Cl5	89.900 (19)
H12A—C12—H12C	109.5	Cl5^ii^—Au2—Cl5	180.0
H12B—C12—H12C	109.5		
			
C2—P1—Se1—Cl1	37.57 (7)	Se1—P1—C2—C21	46.80 (15)
C3—P1—Se1—Cl1	−82.75 (7)	C3—P1—C2—C22	−65.57 (15)
C1—P1—Se1—Cl1	161.60 (7)	C1—P1—C2—C22	59.03 (16)
P1—Se1—Cl1—Cl2	70.10 (8)	Se1—P1—C2—C22	173.95 (11)
C2—P1—C1—C12	45.26 (16)	C2—P1—C3—C32	−50.07 (15)
C3—P1—C1—C12	169.97 (13)	C1—P1—C3—C32	−178.81 (13)
Se1—P1—C1—C12	−74.02 (13)	Se1—P1—C3—C32	70.38 (13)
C2—P1—C1—C11	−81.65 (16)	C2—P1—C3—C31	−174.96 (13)
C3—P1—C1—C11	43.06 (16)	C1—P1—C3—C31	56.30 (15)
Se1—P1—C1—C11	159.07 (13)	Se1—P1—C3—C31	−54.51 (15)
C3—P1—C2—C21	167.28 (14)	Cl3—Au1—Cl2—Cl1	−74.650 (17)
C1—P1—C2—C21	−68.12 (17)	Cl3^i^—Au1—Cl2—Cl1	105.350 (17)

Symmetry codes: (i) −*x*+1, −*y*, −*z*; (ii) −*x*+2, −*y*+2, −*z*+1.

### (Chloroselanyl)tris(propan-2-yl)phosphonium tetrachloridoaurate(III) (21a) Hydrogen-bond geometry (Å, º)

**Table:**

*D*—H···*A*	*D*—H	H···*A*	*D*···*A*	*D*—H···*A*
C21—H21*B*···Cl1	0.98	2.84	3.545 (2)	129
C32—H32*C*···Cl1	0.98	2.95	3.712 (2)	135
C1—H1···Cl5	1.00	2.93	3.8615 (19)	156
C2—H2···Cl2^i^	1.00	2.82	3.6147 (19)	137
C3—H3···Cl2^iii^	1.00	2.94	3.5249 (19)	118
C31—H31*C*···Cl2^iii^	0.98	2.98	3.626 (2)	125
C21—H21*C*···Cl2^iv^	0.98	2.98	3.868 (2)	151
C11—H11*C*···Cl4^iv^	0.98	2.87	3.828 (2)	166
C1—H1···Cl4^ii^	1.00	2.78	3.5341 (19)	133

Symmetry codes: (i) −*x*+1, −*y*, −*z*; (ii) −*x*+2, −*y*+2, −*z*+1; (iii) *x*, *y*+1, *z*; (iv) *x*−1, *y*, *z*.

### (tert-Butyl)(chloroselanyl)bis(propan-2-yl)phosphonium tetrachloridoaurate(III) (22a) Crystal data

**Table:**

(C_10_H_23_ClPSe)[AuCl_4_]	*Z* = 2
*M**_r_* = 627.43	*F*(000) = 592
Triclinic, *P*1	*D*_x_ = 2.245 Mg m^−^^3^
*a* = 7.5836 (2) Å	Mo *K*α radiation, λ = 0.71073 Å
*b* = 8.7603 (2) Å	Cell parameters from 37919 reflections
*c* = 15.4719 (6) Å	θ = 2.5–30.8°
α = 84.445 (3)°	µ = 10.67 mm^−^^1^
β = 78.641 (3)°	*T* = 100 K
γ = 67.128 (3)°	Plate, yellow
*V* = 928.28 (5) Å^3^	0.2 × 0.2 × 0.03 mm

### (tert-Butyl)(chloroselanyl)bis(propan-2-yl)phosphonium tetrachloridoaurate(III) (22a) Data collection

**Table:**

Oxford Diffraction Xcalibur, Eos diffractometer	5490 independent reflections
Radiation source: Enhance (Mo) X-ray Source	4978 reflections with *I* > 2σ(*I*)
Detector resolution: 16.1419 pixels mm^-1^	*R*_int_ = 0.042
ω scan	θ_max_ = 30.9°, θ_min_ = 2.5°
Absorption correction: multi-scan (CrysAlisPro; Rigaku OD, 2015)	*h* = −10→10
*T*_min_ = 0.232, *T*_max_ = 1.000	*k* = −12→12
102916 measured reflections	*l* = −22→21

### (tert-Butyl)(chloroselanyl)bis(propan-2-yl)phosphonium tetrachloridoaurate(III) (22a) Refinement

**Table:**

Refinement on *F*^2^	Secondary atom site location: difference Fourier map
Least-squares matrix: full	Hydrogen site location: inferred from neighbouring sites
*R*[*F*^2^ > 2σ(*F*^2^)] = 0.021	H-atom parameters constrained
*wR*(*F*^2^) = 0.054	*w* = 1/[σ^2^(*F*_o_^2^) + (0.0304*P*)^2^ + 1.3105*P*] where *P* = (*F*_o_^2^ + 2*F*_c_^2^)/3
*S* = 1.05	(Δ/σ)_max_ < 0.001
5490 reflections	Δρ_max_ = 1.86 e Å^−^^3^
174 parameters	Δρ_min_ = −1.59 e Å^−^^3^
0 restraints	Extinction correction: *SHELXL2019/3* (Sheldrick, 2015), *F*_c_^*^ = *kF*_c_[1 + 0.001 *F*_c_^2^λ^3^/sin(2θ)]^-1/4^
Primary atom site location: structure-invariant direct methods	Extinction coefficient: 0.00144 (18)

### (tert-Butyl)(chloroselanyl)bis(propan-2-yl)phosphonium tetrachloridoaurate(III) (22a) Fractional atomic coordinates and isotropic or equivalent isotropic displacement parameters (Å^2^)

**Table:**

	*x*	*y*	*z*	*U*_iso_*/*U*_eq_	
P1	0.50453 (9)	0.10337 (8)	0.25119 (4)	0.01197 (12)	
Se1	0.37816 (4)	−0.07476 (3)	0.32514 (2)	0.01739 (6)	
Cl1	0.14673 (9)	0.09827 (9)	0.41498 (5)	0.02223 (14)	
C1	0.7478 (4)	−0.0370 (3)	0.19534 (17)	0.0146 (5)	
C2	0.5057 (4)	0.2483 (4)	0.32872 (18)	0.0172 (5)	
H2	0.366050	0.317873	0.349742	0.021*	
C3	0.3524 (4)	0.2280 (3)	0.17101 (17)	0.0148 (5)	
H3	0.427966	0.287982	0.132381	0.018*	
C11	0.8138 (4)	0.0486 (4)	0.11101 (18)	0.0198 (5)	
H11A	0.810433	0.156260	0.125582	0.030*	
H11B	0.726486	0.065134	0.068933	0.030*	
H11C	0.946608	−0.020860	0.084707	0.030*	
C12	0.8939 (4)	−0.0783 (4)	0.25882 (19)	0.0204 (6)	
H12A	1.018092	−0.162290	0.232768	0.031*	
H12B	0.843022	−0.120954	0.314952	0.031*	
H12C	0.913544	0.022189	0.269124	0.031*	
C13	0.7372 (4)	−0.2000 (4)	0.1719 (2)	0.0203 (5)	
H13A	0.634986	−0.174976	0.136537	0.030*	
H13B	0.707357	−0.259370	0.226264	0.030*	
H13C	0.862342	−0.269091	0.138141	0.030*	
C21	0.5903 (4)	0.1724 (4)	0.41189 (19)	0.0256 (6)	
H21A	0.732489	0.124501	0.397086	0.038*	
H21B	0.542479	0.085370	0.436270	0.038*	
H21C	0.550277	0.258607	0.455628	0.038*	
C22	0.5972 (4)	0.3690 (4)	0.2794 (2)	0.0235 (6)	
H22A	0.584058	0.453563	0.319917	0.035*	
H22B	0.530347	0.422470	0.229941	0.035*	
H22C	0.735163	0.307435	0.256997	0.035*	
C31	0.3105 (4)	0.1225 (4)	0.1106 (2)	0.0231 (6)	
H31A	0.232105	0.064530	0.145738	0.035*	
H31B	0.433392	0.041165	0.081395	0.035*	
H31C	0.239274	0.194101	0.066081	0.035*	
C32	0.1628 (4)	0.3603 (4)	0.2149 (2)	0.0217 (6)	
H32A	0.087449	0.423452	0.169656	0.033*	
H32B	0.192661	0.435385	0.247387	0.033*	
H32C	0.087009	0.306743	0.255859	0.033*	
Au1	0.000000	0.500000	0.500000	0.01294 (5)	
Cl2	−0.17349 (10)	0.35513 (9)	0.57968 (5)	0.02250 (14)	
Cl3	0.21088 (11)	0.41781 (10)	0.59700 (5)	0.02634 (15)	
Au2	0.500000	0.500000	0.000000	0.01263 (5)	
Cl4	0.34763 (10)	0.69883 (9)	0.10371 (5)	0.02299 (14)	
Cl5	0.79509 (9)	0.46997 (9)	0.02957 (5)	0.02043 (13)	

### (tert-Butyl)(chloroselanyl)bis(propan-2-yl)phosphonium tetrachloridoaurate(III) (22a) Atomic displacement parameters (Å^2^)

**Table:**

	*U*^11^	*U*^22^	*U*^33^	*U*^12^	*U*^13^	*U*^23^
P1	0.0108 (3)	0.0126 (3)	0.0126 (3)	−0.0047 (2)	−0.0017 (2)	−0.0001 (2)
Se1	0.01475 (12)	0.01428 (14)	0.02174 (13)	−0.00624 (10)	0.00013 (9)	0.00256 (10)
Cl1	0.0172 (3)	0.0237 (4)	0.0233 (3)	−0.0085 (3)	0.0041 (2)	−0.0014 (3)
C1	0.0132 (11)	0.0149 (13)	0.0136 (11)	−0.0038 (9)	−0.0012 (9)	0.0005 (9)
C2	0.0176 (12)	0.0188 (14)	0.0170 (12)	−0.0084 (10)	−0.0020 (9)	−0.0043 (10)
C3	0.0132 (11)	0.0136 (13)	0.0170 (11)	−0.0040 (9)	−0.0044 (9)	0.0010 (9)
C11	0.0168 (12)	0.0227 (15)	0.0161 (12)	−0.0063 (11)	0.0023 (9)	0.0019 (10)
C12	0.0122 (11)	0.0222 (15)	0.0249 (14)	−0.0031 (10)	−0.0057 (10)	−0.0015 (11)
C13	0.0177 (12)	0.0153 (14)	0.0254 (14)	−0.0038 (10)	−0.0025 (10)	−0.0030 (11)
C21	0.0252 (14)	0.0386 (19)	0.0162 (13)	−0.0148 (13)	−0.0042 (10)	−0.0023 (12)
C22	0.0244 (14)	0.0252 (16)	0.0259 (14)	−0.0152 (12)	−0.0017 (11)	−0.0042 (12)
C31	0.0255 (14)	0.0206 (15)	0.0252 (14)	−0.0075 (12)	−0.0108 (11)	−0.0012 (11)
C32	0.0164 (12)	0.0194 (15)	0.0254 (14)	−0.0018 (11)	−0.0061 (10)	0.0009 (11)
Au1	0.01396 (7)	0.01208 (8)	0.01319 (7)	−0.00517 (5)	−0.00260 (5)	−0.00061 (5)
Cl2	0.0228 (3)	0.0210 (4)	0.0253 (3)	−0.0122 (3)	−0.0014 (2)	0.0031 (3)
Cl3	0.0248 (3)	0.0352 (4)	0.0230 (3)	−0.0134 (3)	−0.0119 (3)	0.0061 (3)
Au2	0.01037 (7)	0.01051 (8)	0.01674 (7)	−0.00433 (5)	−0.00129 (5)	0.00044 (5)
Cl4	0.0187 (3)	0.0208 (4)	0.0273 (3)	−0.0049 (3)	−0.0004 (2)	−0.0090 (3)
Cl5	0.0132 (3)	0.0206 (3)	0.0289 (3)	−0.0074 (2)	−0.0051 (2)	−0.0001 (3)

### (tert-Butyl)(chloroselanyl)bis(propan-2-yl)phosphonium tetrachloridoaurate(III) (22a) Geometric parameters (Å, º)

**Table:**

P1—C2	1.833 (3)	C13—H13B	0.9800
P1—C3	1.839 (3)	C13—H13C	0.9800
P1—C1	1.864 (3)	C21—H21A	0.9800
P1—Se1	2.2467 (7)	C21—H21B	0.9800
Se1—Cl1	2.1654 (7)	C21—H21C	0.9800
Cl1—Cl2	3.4465 (10)	C22—H22A	0.9800
C1—C11	1.536 (4)	C22—H22B	0.9800
C1—C12	1.540 (4)	C22—H22C	0.9800
C1—C13	1.542 (4)	C31—H31A	0.9800
C2—C21	1.529 (4)	C31—H31B	0.9800
C2—C22	1.545 (4)	C31—H31C	0.9800
C2—H2	1.0000	C32—H32A	0.9800
C3—C31	1.530 (4)	C32—H32B	0.9800
C3—C32	1.531 (4)	C32—H32C	0.9800
C3—H3	1.0000	Au1—Cl3	2.2736 (7)
C11—H11A	0.9800	Au1—Cl3^i^	2.2737 (7)
C11—H11B	0.9800	Au1—Cl2	2.2842 (7)
C11—H11C	0.9800	Au1—Cl2^i^	2.2842 (7)
C12—H12A	0.9800	Au2—Cl4	2.2781 (7)
C12—H12B	0.9800	Au2—Cl4^ii^	2.2781 (7)
C12—H12C	0.9800	Au2—Cl5^ii^	2.2823 (6)
C13—H13A	0.9800	Au2—Cl5	2.2823 (6)
			
C2—P1—C3	106.87 (13)	C1—C13—H13C	109.5
C2—P1—C1	115.83 (12)	H13A—C13—H13C	109.5
C3—P1—C1	111.60 (12)	H13B—C13—H13C	109.5
C2—P1—Se1	109.29 (9)	C2—C21—H21A	109.5
C3—P1—Se1	110.42 (9)	C2—C21—H21B	109.5
C1—P1—Se1	102.77 (9)	H21A—C21—H21B	109.5
Cl1—Se1—P1	98.35 (3)	C2—C21—H21C	109.5
Se1—Cl1—Cl2	171.45 (3)	H21A—C21—H21C	109.5
C11—C1—C12	110.2 (2)	H21B—C21—H21C	109.5
C11—C1—C13	109.8 (2)	C2—C22—H22A	109.5
C12—C1—C13	108.4 (2)	C2—C22—H22B	109.5
C11—C1—P1	109.60 (18)	H22A—C22—H22B	109.5
C12—C1—P1	108.97 (18)	C2—C22—H22C	109.5
C13—C1—P1	109.90 (17)	H22A—C22—H22C	109.5
C21—C2—C22	112.3 (2)	H22B—C22—H22C	109.5
C21—C2—P1	116.6 (2)	C3—C31—H31A	109.5
C22—C2—P1	110.04 (19)	C3—C31—H31B	109.5
C21—C2—H2	105.7	H31A—C31—H31B	109.5
C22—C2—H2	105.7	C3—C31—H31C	109.5
P1—C2—H2	105.7	H31A—C31—H31C	109.5
C31—C3—C32	110.5 (2)	H31B—C31—H31C	109.5
C31—C3—P1	112.97 (19)	C3—C32—H32A	109.5
C32—C3—P1	112.57 (19)	C3—C32—H32B	109.5
C31—C3—H3	106.8	H32A—C32—H32B	109.5
C32—C3—H3	106.8	C3—C32—H32C	109.5
P1—C3—H3	106.8	H32A—C32—H32C	109.5
C1—C11—H11A	109.5	H32B—C32—H32C	109.5
C1—C11—H11B	109.5	Cl3—Au1—Cl3^i^	180.0
H11A—C11—H11B	109.5	Cl3—Au1—Cl2	90.35 (3)
C1—C11—H11C	109.5	Cl3^i^—Au1—Cl2	89.65 (3)
H11A—C11—H11C	109.5	Cl3—Au1—Cl2^i^	89.65 (3)
H11B—C11—H11C	109.5	Cl3^i^—Au1—Cl2^i^	90.35 (3)
C1—C12—H12A	109.5	Cl2—Au1—Cl2^i^	180.00 (3)
C1—C12—H12B	109.5	Au1—Cl2—Cl1	73.52 (2)
H12A—C12—H12B	109.5	Cl4—Au2—Cl4^ii^	180.0
C1—C12—H12C	109.5	Cl4—Au2—Cl5^ii^	89.59 (3)
H12A—C12—H12C	109.5	Cl4^ii^—Au2—Cl5^ii^	90.41 (3)
H12B—C12—H12C	109.5	Cl4—Au2—Cl5	90.41 (3)
C1—C13—H13A	109.5	Cl4^ii^—Au2—Cl5	89.59 (3)
C1—C13—H13B	109.5	Cl5^ii^—Au2—Cl5	180.0
H13A—C13—H13B	109.5		
			
C2—P1—Se1—Cl1	39.96 (10)	C1—P1—C2—C21	−66.3 (2)
C3—P1—Se1—Cl1	−77.33 (10)	Se1—P1—C2—C21	49.2 (2)
C1—P1—Se1—Cl1	163.52 (9)	C3—P1—C2—C22	−62.0 (2)
C2—P1—C1—C11	−87.7 (2)	C1—P1—C2—C22	63.1 (2)
C3—P1—C1—C11	34.9 (2)	Se1—P1—C2—C22	178.52 (17)
Se1—P1—C1—C11	153.19 (17)	C2—P1—C3—C31	−170.76 (19)
C2—P1—C1—C12	32.9 (2)	C1—P1—C3—C31	61.7 (2)
C3—P1—C1—C12	155.51 (18)	Se1—P1—C3—C31	−52.0 (2)
Se1—P1—C1—C12	−86.16 (19)	C2—P1—C3—C32	−44.8 (2)
C2—P1—C1—C13	151.59 (18)	C1—P1—C3—C32	−172.36 (19)
C3—P1—C1—C13	−85.8 (2)	Se1—P1—C3—C32	74.0 (2)
Se1—P1—C1—C13	32.50 (19)	Cl3—Au1—Cl2—Cl1	−94.93 (3)
C3—P1—C2—C21	168.7 (2)	Cl3^i^—Au1—Cl2—Cl1	85.07 (3)

Symmetry codes: (i) −*x*, −*y*+1, −*z*+1; (ii) −*x*+1, −*y*+1, −*z*.

### (tert-Butyl)(chloroselanyl)bis(propan-2-yl)phosphonium tetrachloridoaurate(III) (22a) Hydrogen-bond geometry (Å, º)

**Table:**

*D*—H···*A*	*D*—H	H···*A*	*D*···*A*	*D*—H···*A*
C13—H13*B*···Se1	0.98	2.65	3.142 (3)	111
C2—H2···Cl1	1.00	2.99	3.454 (3)	110
C3—H3···Au2	1.00	2.75	3.690 (3)	157
C32—H32*C*···Cl1	0.98	2.92	3.682 (3)	135
C21—H21*C*···Cl3	0.98	2.99	3.774 (3)	138
C11—H11*A*···Cl5	0.98	2.97	3.741 (3)	137
C2—H2···Cl2^i^	1.00	2.87	3.629 (3)	133
C22—H22*A*···Cl3^iii^	0.98	2.79	3.629 (3)	144
C12—H12*B*···Cl3^iv^	0.98	2.95	3.783 (3)	143
C13—H13*B*···Cl3^iv^	0.98	3.01	3.902 (3)	152
C12—H12*A*···Cl4^v^	0.98	2.80	3.721 (3)	156
C13—H13*A*···Cl4^vi^	0.98	2.94	3.751 (3)	141

Symmetry codes: (i) −*x*, −*y*+1, −*z*+1; (iii) −*x*+1, −*y*+1, −*z*+1; (iv) −*x*+1, −*y*, −*z*+1; (v) *x*+1, *y*−1, *z*; (vi) *x*, *y*−1, *z*.

### Bis(tert-butyl)(chloroselanyl)(propan-2-yl)phosphonium tetrachloridoaurate(III) (23a) Crystal data

**Table:**

(C_11_H_25_ClPSe)[AuCl_4_]	*F*(000) = 2432
*M**_r_* = 641.46	*D*_x_ = 2.175 Mg m^−^^3^
Monoclinic, *C*2/*c*	Mo *K*α radiation, λ = 0.71073 Å
*a* = 33.7472 (6) Å	Cell parameters from 55878 reflections
*b* = 7.79306 (8) Å	θ = 2.2–30.9°
*c* = 16.2575 (3) Å	µ = 10.12 mm^−^^1^
β = 113.582 (3)°	*T* = 100 K
*V* = 3918.54 (14) Å^3^	Plate, yellow
*Z* = 8	0.35 × 0.2 × 0.04 mm

### Bis(tert-butyl)(chloroselanyl)(propan-2-yl)phosphonium tetrachloridoaurate(III) (23a) Data collection

**Table:**

Oxford Diffraction Xcalibur, Eos diffractometer	6028 independent reflections
Radiation source: Enhance (Mo) X-ray Source	5453 reflections with *I* > 2σ(*I*)
Detector resolution: 16.1419 pixels mm^-1^	*R*_int_ = 0.058
ω scan	θ_max_ = 30.9°, θ_min_ = 2.5°
Absorption correction: multi-scan (CrysAlisPro; Rigaku OD, 2015)	*h* = −48→47
*T*_min_ = 0.308, *T*_max_ = 1.000	*k* = −11→11
257492 measured reflections	*l* = −23→23

### Bis(tert-butyl)(chloroselanyl)(propan-2-yl)phosphonium tetrachloridoaurate(III) (23a) Refinement

**Table:**

Refinement on *F*^2^	Primary atom site location: structure-invariant direct methods
Least-squares matrix: full	Secondary atom site location: difference Fourier map
*R*[*F*^2^ > 2σ(*F*^2^)] = 0.021	Hydrogen site location: inferred from neighbouring sites
*wR*(*F*^2^) = 0.043	H-atom parameters constrained
*S* = 1.11	*w* = 1/[σ^2^(*F*_o_^2^) + (0.0165*P*)^2^ + 12.3178*P*] where *P* = (*F*_o_^2^ + 2*F*_c_^2^)/3
6028 reflections	(Δ/σ)_max_ = 0.002
191 parameters	Δρ_max_ = 1.13 e Å^−^^3^
0 restraints	Δρ_min_ = −1.20 e Å^−^^3^

### Bis(tert-butyl)(chloroselanyl)(propan-2-yl)phosphonium tetrachloridoaurate(III) (23a) Special details

**Table:**

Refinement. The anion centred on Au1 is disordered, with the atoms Cl3 and Cl4 being slightly displaced from the twofold axis. Dimensions of disordered groups should be interpreted with caution.

### Bis(tert-butyl)(chloroselanyl)(propan-2-yl)phosphonium tetrachloridoaurate(III) (23a) Fractional atomic coordinates and isotropic or equivalent isotropic displacement parameters (Å^2^)

**Table:**

	*x*	*y*	*z*	*U*_iso_*/*U*_eq_	Occ. (<1)
P1	0.37529 (2)	0.60748 (7)	0.40097 (4)	0.01109 (11)	
Se1	0.42555 (2)	0.76990 (3)	0.50633 (2)	0.01833 (5)	
Cl1	0.38551 (2)	0.90386 (9)	0.56139 (4)	0.02734 (14)	
C2	0.34626 (8)	0.7484 (3)	0.30151 (15)	0.0141 (4)	
C1	0.41121 (8)	0.4391 (3)	0.38588 (15)	0.0138 (4)	
C3	0.33619 (8)	0.5250 (3)	0.44412 (15)	0.0137 (4)	
H3	0.315469	0.621002	0.437455	0.016*	
C23	0.33117 (9)	0.9092 (3)	0.33558 (17)	0.0197 (5)	
H23A	0.314377	0.982549	0.284553	0.030*	
H23B	0.356412	0.972528	0.376659	0.030*	
H23C	0.313092	0.874894	0.367276	0.030*	
C21	0.30654 (8)	0.6599 (3)	0.23160 (16)	0.0168 (5)	
H21A	0.294574	0.731319	0.177595	0.025*	
H21B	0.284670	0.643492	0.256332	0.025*	
H21C	0.314980	0.548066	0.216138	0.025*	
C22	0.37709 (9)	0.8055 (3)	0.25826 (17)	0.0202 (5)	
H22A	0.385158	0.705974	0.231496	0.030*	
H22B	0.403128	0.856211	0.304167	0.030*	
H22C	0.362643	0.890849	0.211536	0.030*	
C11	0.42408 (8)	0.3114 (3)	0.46488 (17)	0.0186 (5)	
H11A	0.445045	0.228858	0.460457	0.028*	
H11B	0.398317	0.250180	0.462671	0.028*	
H11C	0.437065	0.374233	0.521677	0.028*	
C12	0.38849 (8)	0.3426 (3)	0.29689 (17)	0.0188 (5)	
H12A	0.407547	0.251957	0.291947	0.028*	
H12B	0.381773	0.422950	0.246727	0.028*	
H12C	0.361655	0.291381	0.295193	0.028*	
C13	0.45260 (8)	0.5246 (3)	0.38698 (18)	0.0204 (5)	
H13A	0.467873	0.581618	0.444851	0.031*	
H13B	0.444862	0.609482	0.338592	0.031*	
H13C	0.471389	0.437022	0.378194	0.031*	
C32	0.30876 (8)	0.3748 (3)	0.38931 (18)	0.0191 (5)	
H32A	0.326583	0.270978	0.401435	0.029*	
H32B	0.297854	0.402248	0.325203	0.029*	
H32C	0.284348	0.355387	0.406317	0.029*	
C31	0.35545 (9)	0.4778 (4)	0.54445 (17)	0.0227 (5)	
H31A	0.332039	0.463082	0.565031	0.034*	
H31B	0.374783	0.569670	0.578924	0.034*	
H31C	0.371774	0.370435	0.553273	0.034*	
Au1	0.500000	1.01052 (2)	0.750000	0.01363 (3)	
Cl2	0.51056 (2)	1.01088 (7)	0.61977 (4)	0.01812 (11)	
Cl3	0.49010 (7)	1.29947 (15)	0.73350 (16)	0.0338 (6)	0.5
Cl4	0.5054 (3)	0.72026 (14)	0.7548 (10)	0.0235 (13)	0.5
Au2	0.250000	0.750000	0.500000	0.01126 (3)	
Cl5	0.27035 (2)	1.02584 (7)	0.49126 (4)	0.02105 (12)	
Cl6	0.22611 (2)	0.72036 (8)	0.34868 (4)	0.02033 (12)	

### Bis(tert-butyl)(chloroselanyl)(propan-2-yl)phosphonium tetrachloridoaurate(III) (23a) Atomic displacement parameters (Å^2^)

**Table:**

	*U*^11^	*U*^22^	*U*^33^	*U*^12^	*U*^13^	*U*^23^
P1	0.0116 (3)	0.0111 (2)	0.0103 (2)	−0.00052 (19)	0.0041 (2)	−0.00012 (19)
Se1	0.01531 (12)	0.02074 (11)	0.01544 (11)	−0.00326 (9)	0.00247 (9)	−0.00587 (9)
Cl1	0.0316 (4)	0.0280 (3)	0.0228 (3)	0.0020 (3)	0.0112 (3)	−0.0093 (2)
C2	0.0157 (11)	0.0129 (10)	0.0120 (10)	−0.0007 (8)	0.0039 (9)	0.0022 (8)
C1	0.0122 (11)	0.0152 (10)	0.0149 (10)	0.0020 (8)	0.0063 (9)	0.0000 (8)
C3	0.0143 (11)	0.0151 (10)	0.0134 (10)	0.0001 (8)	0.0074 (9)	0.0024 (8)
C23	0.0239 (13)	0.0129 (10)	0.0203 (12)	0.0030 (9)	0.0067 (10)	0.0029 (9)
C21	0.0161 (12)	0.0168 (10)	0.0134 (10)	−0.0013 (9)	0.0017 (9)	0.0005 (8)
C22	0.0229 (13)	0.0204 (11)	0.0188 (11)	−0.0034 (10)	0.0098 (10)	0.0046 (9)
C11	0.0187 (12)	0.0187 (11)	0.0184 (11)	0.0067 (9)	0.0075 (10)	0.0047 (9)
C12	0.0213 (13)	0.0173 (11)	0.0186 (11)	0.0016 (9)	0.0087 (10)	−0.0042 (9)
C13	0.0130 (11)	0.0263 (12)	0.0241 (12)	−0.0007 (9)	0.0096 (10)	−0.0011 (10)
C32	0.0183 (12)	0.0164 (11)	0.0244 (12)	−0.0036 (9)	0.0103 (10)	−0.0002 (9)
C31	0.0266 (14)	0.0289 (13)	0.0156 (11)	−0.0006 (11)	0.0116 (10)	0.0047 (10)
Au1	0.01365 (6)	0.00993 (5)	0.02032 (6)	0.000	0.00996 (5)	0.000
Cl2	0.0196 (3)	0.0176 (2)	0.0199 (3)	−0.0035 (2)	0.0108 (2)	−0.0004 (2)
Cl3	0.065 (2)	0.0113 (4)	0.0477 (18)	0.0045 (6)	0.0463 (14)	0.0033 (5)
Cl4	0.040 (4)	0.0110 (4)	0.021 (2)	0.0037 (7)	0.014 (3)	0.0006 (9)
Au2	0.01106 (6)	0.01076 (5)	0.01175 (5)	0.00169 (4)	0.00433 (4)	0.00043 (4)
Cl5	0.0266 (3)	0.0134 (2)	0.0244 (3)	−0.0031 (2)	0.0115 (2)	0.0005 (2)
Cl6	0.0252 (3)	0.0205 (3)	0.0124 (2)	0.0039 (2)	0.0046 (2)	−0.0003 (2)

### Bis(tert-butyl)(chloroselanyl)(propan-2-yl)phosphonium tetrachloridoaurate(III) (23a) Geometric parameters (Å, º)

**Table:**

P1—C3	1.840 (2)	C11—H11C	0.9800
P1—C1	1.868 (2)	C12—H12A	0.9800
P1—C2	1.875 (2)	C12—H12B	0.9800
P1—Se1	2.2557 (6)	C12—H12C	0.9800
Se1—Cl1	2.1645 (7)	C13—H13A	0.9800
Se1—Cl2	3.3052 (6)	C13—H13B	0.9800
C2—C21	1.532 (3)	C13—H13C	0.9800
C2—C22	1.536 (4)	C32—H32A	0.9800
C2—C23	1.536 (3)	C32—H32B	0.9800
C1—C12	1.536 (3)	C32—H32C	0.9800
C1—C13	1.541 (3)	C31—H31A	0.9800
C1—C11	1.543 (3)	C31—H31B	0.9800
C3—C32	1.537 (3)	C31—H31C	0.9800
C3—C31	1.540 (3)	Au1—Cl4^i^	2.2681 (13)
C3—H3	1.0000	Au1—Cl4	2.2681 (13)
C23—H23A	0.9800	Au1—Cl3^i^	2.2765 (12)
C23—H23B	0.9800	Au1—Cl3	2.2765 (12)
C23—H23C	0.9800	Au1—Cl2	2.2833 (6)
C21—H21A	0.9800	Au1—Cl2^i^	2.2833 (6)
C21—H21B	0.9800	Cl3—Cl3^i^	0.669 (4)
C21—H21C	0.9800	Cl4—Cl4^i^	0.332 (17)
C22—H22A	0.9800	Au2—Cl6^ii^	2.2734 (6)
C22—H22B	0.9800	Au2—Cl6	2.2735 (6)
C22—H22C	0.9800	Au2—Cl5	2.2784 (6)
C11—H11A	0.9800	Au2—Cl5^ii^	2.2785 (6)
C11—H11B	0.9800		
			
C3—P1—C1	113.98 (11)	H11B—C11—H11C	109.5
C3—P1—C2	109.06 (11)	C1—C12—H12A	109.5
C1—P1—C2	116.17 (11)	C1—C12—H12B	109.5
C3—P1—Se1	110.03 (8)	H12A—C12—H12B	109.5
C1—P1—Se1	99.42 (8)	C1—C12—H12C	109.5
C2—P1—Se1	107.51 (7)	H12A—C12—H12C	109.5
Cl1—Se1—P1	100.40 (3)	H12B—C12—H12C	109.5
Cl1—Se1—Cl2	92.37 (2)	C1—C13—H13A	109.5
P1—Se1—Cl2	164.50 (2)	C1—C13—H13B	109.5
C21—C2—C22	110.0 (2)	H13A—C13—H13B	109.5
C21—C2—C23	108.5 (2)	C1—C13—H13C	109.5
C22—C2—C23	108.17 (19)	H13A—C13—H13C	109.5
C21—C2—P1	112.10 (15)	H13B—C13—H13C	109.5
C22—C2—P1	110.40 (17)	C3—C32—H32A	109.5
C23—C2—P1	107.51 (16)	C3—C32—H32B	109.5
C12—C1—C13	109.3 (2)	H32A—C32—H32B	109.5
C12—C1—C11	109.7 (2)	C3—C32—H32C	109.5
C13—C1—C11	108.5 (2)	H32A—C32—H32C	109.5
C12—C1—P1	111.22 (16)	H32B—C32—H32C	109.5
C13—C1—P1	109.11 (16)	C3—C31—H31A	109.5
C11—C1—P1	108.96 (16)	C3—C31—H31B	109.5
C32—C3—C31	109.5 (2)	H31A—C31—H31B	109.5
C32—C3—P1	113.73 (17)	C3—C31—H31C	109.5
C31—C3—P1	115.10 (17)	H31A—C31—H31C	109.5
C32—C3—H3	105.9	H31B—C31—H31C	109.5
C31—C3—H3	105.9	Cl4^i^—Au1—Cl4	8.4 (4)
P1—C3—H3	105.9	Cl4^i^—Au1—Cl3^i^	175.2 (3)
C2—C23—H23A	109.5	Cl4—Au1—Cl3^i^	167.6 (2)
C2—C23—H23B	109.5	Cl4^i^—Au1—Cl3	167.6 (2)
H23A—C23—H23B	109.5	Cl4—Au1—Cl3	175.2 (3)
C2—C23—H23C	109.5	Cl3^i^—Au1—Cl3	16.89 (9)
H23A—C23—H23C	109.5	Cl4^i^—Au1—Cl2	90.5 (4)
H23B—C23—H23C	109.5	Cl4—Au1—Cl2	89.6 (4)
C2—C21—H21A	109.5	Cl3^i^—Au1—Cl2	92.19 (8)
C2—C21—H21B	109.5	Cl3—Au1—Cl2	87.68 (8)
H21A—C21—H21B	109.5	Cl4^i^—Au1—Cl2^i^	89.6 (4)
C2—C21—H21C	109.5	Cl4—Au1—Cl2^i^	90.5 (4)
H21A—C21—H21C	109.5	Cl3^i^—Au1—Cl2^i^	87.67 (8)
H21B—C21—H21C	109.5	Cl3—Au1—Cl2^i^	92.18 (8)
C2—C22—H22A	109.5	Cl2—Au1—Cl2^i^	179.86 (3)
C2—C22—H22B	109.5	Au1—Cl2—Se1	95.398 (19)
H22A—C22—H22B	109.5	Cl3^i^—Cl3—Au1	81.55 (5)
C2—C22—H22C	109.5	Cl4^i^—Cl4—Au1	85.8 (2)
H22A—C22—H22C	109.5	Cl6^ii^—Au2—Cl6	180.00 (3)
H22B—C22—H22C	109.5	Cl6^ii^—Au2—Cl5	89.22 (2)
C1—C11—H11A	109.5	Cl6—Au2—Cl5	90.78 (2)
C1—C11—H11B	109.5	Cl6^ii^—Au2—Cl5^ii^	90.78 (2)
H11A—C11—H11B	109.5	Cl6—Au2—Cl5^ii^	89.22 (2)
C1—C11—H11C	109.5	Cl5—Au2—Cl5^ii^	180.0
H11A—C11—H11C	109.5		
			
C3—P1—Se1—Cl1	−39.56 (8)	Se1—P1—C1—C13	−40.61 (16)
C1—P1—Se1—Cl1	−159.50 (8)	C3—P1—C1—C11	−39.33 (19)
C2—P1—Se1—Cl1	79.09 (9)	C2—P1—C1—C11	−167.41 (16)
C3—P1—Se1—Cl2	175.48 (10)	Se1—P1—C1—C11	77.67 (16)
C1—P1—Se1—Cl2	55.54 (11)	C1—P1—C3—C32	−53.0 (2)
C2—P1—Se1—Cl2	−65.86 (12)	C2—P1—C3—C32	78.60 (19)
C3—P1—C2—C21	−51.5 (2)	Se1—P1—C3—C32	−163.70 (15)
C1—P1—C2—C21	79.0 (2)	C1—P1—C3—C31	74.4 (2)
Se1—P1—C2—C21	−170.76 (15)	C2—P1—C3—C31	−153.95 (18)
C3—P1—C2—C22	−174.55 (16)	Se1—P1—C3—C31	−36.25 (19)
C1—P1—C2—C22	−44.1 (2)	Cl4^i^—Au1—Cl2—Se1	51.0 (2)
Se1—P1—C2—C22	66.17 (17)	Cl4—Au1—Cl2—Se1	59.4 (2)
C3—P1—C2—C23	67.66 (18)	Cl3^i^—Au1—Cl2—Se1	−132.95 (5)
C1—P1—C2—C23	−161.87 (16)	Cl3—Au1—Cl2—Se1	−116.66 (5)
Se1—P1—C2—C23	−51.61 (18)	Cl4^i^—Au1—Cl3—Cl3^i^	173 (2)
C3—P1—C1—C12	81.70 (19)	Cl2—Au1—Cl3—Cl3^i^	−105.4 (5)
C2—P1—C1—C12	−46.4 (2)	Cl2^i^—Au1—Cl3—Cl3^i^	74.6 (5)
Se1—P1—C1—C12	−161.30 (16)	Cl3^i^—Au1—Cl4—Cl4^i^	165 (4)
C3—P1—C1—C13	−157.60 (16)	Cl2—Au1—Cl4—Cl4^i^	−96 (6)
C2—P1—C1—C13	74.31 (19)	Cl2^i^—Au1—Cl4—Cl4^i^	84 (6)

Symmetry codes: (i) −*x*+1, *y*, −*z*+3/2; (ii) −*x*+1/2, −*y*+3/2, −*z*+1.

### Bis(tert-butyl)(chloroselanyl)(propan-2-yl)phosphonium tetrachloridoaurate(III) (23a) Hydrogen-bond geometry (Å, º)

**Table:**

*D*—H···*A*	*D*—H	H···*A*	*D*···*A*	*D*—H···*A*
C3—H3···Au2	1.00	2.95	3.805 (2)	144
C3—H3···Cl6	1.00	2.88	3.730 (2)	143
C13—H13*A*···Se1	0.98	2.52	3.110 (3)	119
C23—H23*B*···Cl1	0.98	2.81	3.385 (3)	118
C31—H31*B*···Cl1	0.98	2.66	3.451 (3)	138
C23—H23*A*···Cl6^iii^	0.98	2.76	3.736 (2)	173
C21—H21*C*···Cl6^iv^	0.98	2.90	3.674 (2)	137
C13—H13*B*···Cl3^v^	0.98	2.80	3.498 (4)	129
C22—H22*B*···Cl2^vi^	0.98	2.86	3.787 (3)	157
C13—H13*C*···Cl4^vii^	0.98	2.85	3.685 (15)	143

Symmetry codes: (iii) −*x*+1/2, *y*+1/2, −*z*+1/2; (iv) −*x*+1/2, *y*−1/2, −*z*+1/2; (v) *x*, −*y*+2, *z*−1/2; (vi) −*x*+1, −*y*+2, −*z*+1; (vii) −*x*+1, −*y*+1, −*z*+1.

### (Bromosulfanyl)tris(propan-2-yl)phosphonium tetrabromidoaurate(III) (17b) Crystal data

**Table:**

(C_9_H_21_BrPS)[AuBr_4_]	*Z* = 2
*M**_r_* = 788.80	*F*(000) = 720
Triclinic, *P*1	*D*_x_ = 2.790 Mg m^−^^3^
*a* = 7.8543 (4) Å	Mo *K*α radiation, λ = 0.71073 Å
*b* = 8.0592 (4) Å	Cell parameters from 18583 reflections
*c* = 15.3505 (7) Å	θ = 2.6–30.8°
α = 76.717 (4)°	µ = 18.65 mm^−^^1^
β = 83.169 (4)°	*T* = 100 K
γ = 87.722 (4)°	Plate, red
*V* = 938.89 (8) Å^3^	0.2 × 0.08 × 0.05 mm

### (Bromosulfanyl)tris(propan-2-yl)phosphonium tetrabromidoaurate(III) (17b) Data collection

**Table:**

Oxford Diffraction Xcalibur, Eos diffractometer	5646 independent reflections
Radiation source: Enhance (Mo) X-ray Source	5089 reflections with *I* > 2σ(*I*)
Detector resolution: 16.1419 pixels mm^-1^	*R*_int_ = 0.043
ω scans	θ_max_ = 30.9°, θ_min_ = 2.6°
Absorption correction: multi-scan (CrysAlisPro; Rigaku OD, 2015)	*h* = −11→11
*T*_min_ = 0.247, *T*_max_ = 1.000	*k* = −11→11
54229 measured reflections	*l* = −22→21

### (Bromosulfanyl)tris(propan-2-yl)phosphonium tetrabromidoaurate(III) (17b) Refinement

**Table:**

Refinement on *F*^2^	Secondary atom site location: difference Fourier map
Least-squares matrix: full	Hydrogen site location: inferred from neighbouring sites
*R*[*F*^2^ > 2σ(*F*^2^)] = 0.021	H-atom parameters constrained
*wR*(*F*^2^) = 0.040	*w* = 1/[σ^2^(*F*_o_^2^) + (0.014*P*)^2^ + 1.1153*P*] where *P* = (*F*_o_^2^ + 2*F*_c_^2^)/3
*S* = 1.08	(Δ/σ)_max_ = 0.001
5646 reflections	Δρ_max_ = 1.42 e Å^−^^3^
164 parameters	Δρ_min_ = −1.15 e Å^−^^3^
0 restraints	Extinction correction: *SHELXL2019/3* (Sheldrick, 2015), *F*_c_^*^ = *kF*_c_[1 + 0.001 *F*_c_^2^λ^3^/sin(2θ)]^-1/4^
Primary atom site location: structure-invariant direct methods	Extinction coefficient: 0.00112 (7)

### (Bromosulfanyl)tris(propan-2-yl)phosphonium tetrabromidoaurate(III) (17b) Fractional atomic coordinates and isotropic or equivalent isotropic displacement parameters (Å^2^)

**Table:**

	*x*	*y*	*z*	*U*_iso_*/*U*_eq_	
P1	0.69771 (9)	0.74853 (9)	0.25622 (5)	0.00958 (13)	
S1	0.88558 (9)	0.55851 (9)	0.28013 (5)	0.01523 (14)	
Br1	0.85534 (4)	0.43166 (4)	0.17064 (2)	0.01714 (6)	
C1	0.7070 (4)	0.8436 (4)	0.35348 (18)	0.0129 (5)	
H1	0.831259	0.859646	0.357245	0.015*	
C2	0.4956 (4)	0.6524 (3)	0.24812 (18)	0.0119 (5)	
H2	0.512417	0.609080	0.191623	0.014*	
C3	0.7561 (4)	0.9104 (3)	0.15342 (18)	0.0113 (5)	
H3	0.681871	1.012659	0.157636	0.014*	
C11	0.6231 (4)	1.0202 (4)	0.3420 (2)	0.0189 (6)	
H11A	0.500205	1.011195	0.338359	0.028*	
H11B	0.675956	1.095104	0.286560	0.028*	
H11C	0.639301	1.067813	0.393673	0.028*	
C12	0.6375 (4)	0.7254 (4)	0.44330 (19)	0.0182 (6)	
H12A	0.674091	0.766691	0.493334	0.027*	
H12B	0.682260	0.609468	0.445424	0.027*	
H12C	0.511981	0.724814	0.448393	0.027*	
C21	0.4448 (4)	0.4985 (4)	0.3250 (2)	0.0186 (6)	
H21A	0.409512	0.537193	0.380510	0.028*	
H21B	0.542985	0.419964	0.333736	0.028*	
H21C	0.349316	0.439793	0.309798	0.028*	
C22	0.3521 (4)	0.7878 (4)	0.2357 (2)	0.0172 (6)	
H22A	0.250375	0.738280	0.220752	0.026*	
H22B	0.390742	0.884531	0.186696	0.026*	
H22C	0.323445	0.826977	0.291591	0.026*	
C31	0.9412 (4)	0.9658 (4)	0.14881 (19)	0.0165 (6)	
H31A	1.019276	0.872793	0.138121	0.025*	
H31B	0.959506	0.993536	0.205860	0.025*	
H31C	0.963641	1.066466	0.099479	0.025*	
C32	0.7224 (4)	0.8589 (4)	0.06692 (19)	0.0178 (6)	
H32A	0.746424	0.955373	0.015331	0.027*	
H32B	0.602194	0.826255	0.071665	0.027*	
H32C	0.797026	0.762154	0.058485	0.027*	
Au1	0.500000	0.500000	0.000000	0.01133 (4)	
Br2	0.76076 (4)	0.33440 (4)	−0.01670 (2)	0.01959 (7)	
Br3	0.39092 (4)	0.28650 (4)	0.13213 (2)	0.01803 (7)	
Au2	1.000000	0.000000	0.500000	0.00940 (4)	
Br4	1.11077 (4)	0.13466 (4)	0.34673 (2)	0.01512 (6)	
Br5	1.10194 (4)	−0.27647 (4)	0.47679 (2)	0.01735 (6)	

### (Bromosulfanyl)tris(propan-2-yl)phosphonium tetrabromidoaurate(III) (17b) Atomic displacement parameters (Å^2^)

**Table:**

	*U*^11^	*U*^22^	*U*^33^	*U*^12^	*U*^13^	*U*^23^
P1	0.0090 (3)	0.0109 (3)	0.0085 (3)	−0.0001 (2)	−0.0009 (2)	−0.0015 (2)
S1	0.0135 (4)	0.0151 (3)	0.0168 (3)	0.0036 (3)	−0.0037 (3)	−0.0026 (3)
Br1	0.01764 (15)	0.01416 (13)	0.01919 (14)	0.00246 (11)	0.00120 (11)	−0.00502 (11)
C1	0.0127 (14)	0.0166 (13)	0.0100 (12)	−0.0035 (10)	−0.0018 (10)	−0.0032 (10)
C2	0.0112 (14)	0.0143 (13)	0.0112 (12)	−0.0039 (10)	−0.0006 (10)	−0.0045 (10)
C3	0.0119 (14)	0.0119 (13)	0.0092 (12)	−0.0001 (10)	−0.0005 (10)	−0.0010 (9)
C11	0.0240 (17)	0.0168 (14)	0.0178 (14)	0.0004 (12)	−0.0031 (12)	−0.0075 (11)
C12	0.0220 (17)	0.0227 (15)	0.0099 (13)	−0.0041 (12)	−0.0003 (11)	−0.0037 (11)
C21	0.0204 (16)	0.0180 (15)	0.0161 (14)	−0.0072 (12)	0.0014 (12)	−0.0017 (11)
C22	0.0093 (14)	0.0205 (15)	0.0218 (15)	−0.0001 (11)	−0.0006 (11)	−0.0050 (12)
C31	0.0151 (15)	0.0183 (14)	0.0139 (13)	−0.0032 (11)	0.0002 (11)	0.0006 (11)
C32	0.0224 (17)	0.0185 (14)	0.0115 (13)	−0.0030 (12)	−0.0006 (12)	−0.0017 (11)
Au1	0.01375 (8)	0.01132 (7)	0.00895 (7)	0.00043 (5)	−0.00182 (5)	−0.00222 (5)
Br2	0.01882 (16)	0.01990 (15)	0.01867 (14)	0.00610 (11)	−0.00027 (12)	−0.00357 (11)
Br3	0.02203 (16)	0.01692 (14)	0.01267 (13)	−0.00162 (11)	0.00002 (11)	0.00091 (10)
Au2	0.00830 (7)	0.01071 (7)	0.00965 (7)	−0.00008 (5)	−0.00162 (5)	−0.00296 (5)
Br4	0.01749 (15)	0.01523 (13)	0.01148 (12)	−0.00092 (10)	0.00137 (10)	−0.00208 (10)
Br5	0.02171 (16)	0.01346 (13)	0.01655 (13)	0.00486 (11)	−0.00061 (11)	−0.00435 (10)

### (Bromosulfanyl)tris(propan-2-yl)phosphonium tetrabromidoaurate(III) (17b) Geometric parameters (Å, º)

**Table:**

P1—C2	1.823 (3)	C21—H21A	0.9800
P1—C3	1.825 (3)	C21—H21B	0.9800
P1—C1	1.837 (3)	C21—H21C	0.9800
P1—S1	2.0852 (10)	C22—H22A	0.9800
S1—Br1	2.1977 (8)	C22—H22B	0.9800
Br1—Br2	3.3206 (5)	C22—H22C	0.9800
C1—C11	1.527 (4)	C31—H31A	0.9800
C1—C12	1.538 (4)	C31—H31B	0.9800
C1—H1	1.0000	C31—H31C	0.9800
C2—C21	1.531 (4)	C32—H32A	0.9800
C2—C22	1.535 (4)	C32—H32B	0.9800
C2—H2	1.0000	C32—H32C	0.9800
C3—C31	1.526 (4)	Au1—Br2	2.4201 (3)
C3—C32	1.533 (4)	Au1—Br2^i^	2.4201 (3)
C3—H3	1.0000	Au1—Br3^i^	2.4307 (3)
C11—H11A	0.9800	Au1—Br3	2.4308 (3)
C11—H11B	0.9800	Au2—Br4^ii^	2.4259 (3)
C11—H11C	0.9800	Au2—Br4	2.4259 (3)
C12—H12A	0.9800	Au2—Br5	2.4265 (3)
C12—H12B	0.9800	Au2—Br5^ii^	2.4265 (3)
C12—H12C	0.9800		
			
C2—P1—C3	109.37 (13)	C2—C21—H21A	109.5
C2—P1—C1	116.89 (13)	C2—C21—H21B	109.5
C3—P1—C1	108.56 (13)	H21A—C21—H21B	109.5
C2—P1—S1	109.41 (10)	C2—C21—H21C	109.5
C3—P1—S1	112.29 (10)	H21A—C21—H21C	109.5
C1—P1—S1	100.11 (10)	H21B—C21—H21C	109.5
P1—S1—Br1	99.46 (4)	C2—C22—H22A	109.5
S1—Br1—Br2	165.22 (2)	C2—C22—H22B	109.5
C11—C1—C12	111.3 (2)	H22A—C22—H22B	109.5
C11—C1—P1	113.1 (2)	C2—C22—H22C	109.5
C12—C1—P1	112.9 (2)	H22A—C22—H22C	109.5
C11—C1—H1	106.3	H22B—C22—H22C	109.5
C12—C1—H1	106.3	C3—C31—H31A	109.5
P1—C1—H1	106.3	C3—C31—H31B	109.5
C21—C2—C22	112.2 (2)	H31A—C31—H31B	109.5
C21—C2—P1	114.3 (2)	C3—C31—H31C	109.5
C22—C2—P1	110.73 (19)	H31A—C31—H31C	109.5
C21—C2—H2	106.3	H31B—C31—H31C	109.5
C22—C2—H2	106.3	C3—C32—H32A	109.5
P1—C2—H2	106.3	C3—C32—H32B	109.5
C31—C3—C32	111.6 (2)	H32A—C32—H32B	109.5
C31—C3—P1	110.68 (19)	C3—C32—H32C	109.5
C32—C3—P1	114.0 (2)	H32A—C32—H32C	109.5
C31—C3—H3	106.7	H32B—C32—H32C	109.5
C32—C3—H3	106.7	Br2—Au1—Br2^i^	180.0
P1—C3—H3	106.7	Br2—Au1—Br3^i^	89.288 (11)
C1—C11—H11A	109.5	Br2^i^—Au1—Br3^i^	90.711 (11)
C1—C11—H11B	109.5	Br2—Au1—Br3	90.712 (11)
H11A—C11—H11B	109.5	Br2^i^—Au1—Br3	89.289 (11)
C1—C11—H11C	109.5	Br3^i^—Au1—Br3	180.0
H11A—C11—H11C	109.5	Au1—Br2—Br1	86.521 (11)
H11B—C11—H11C	109.5	Br4^ii^—Au2—Br4	180.0
C1—C12—H12A	109.5	Br4^ii^—Au2—Br5	89.307 (10)
C1—C12—H12B	109.5	Br4—Au2—Br5	90.692 (10)
H12A—C12—H12B	109.5	Br4^ii^—Au2—Br5^ii^	90.693 (10)
C1—C12—H12C	109.5	Br4—Au2—Br5^ii^	89.308 (10)
H12A—C12—H12C	109.5	Br5—Au2—Br5^ii^	180.0
H12B—C12—H12C	109.5		
			
C2—P1—S1—Br1	51.56 (10)	C1—P1—C2—C21	−63.8 (2)
C3—P1—S1—Br1	−70.07 (10)	S1—P1—C2—C21	49.0 (2)
C1—P1—S1—Br1	174.93 (9)	C3—P1—C2—C22	−59.7 (2)
P1—S1—Br1—Br2	19.42 (11)	C1—P1—C2—C22	64.1 (2)
C2—P1—C1—C11	−79.3 (2)	S1—P1—C2—C22	176.91 (17)
C3—P1—C1—C11	44.9 (2)	C2—P1—C3—C31	−167.66 (19)
S1—P1—C1—C11	162.73 (19)	C1—P1—C3—C31	63.7 (2)
C2—P1—C1—C12	48.3 (3)	S1—P1—C3—C31	−46.0 (2)
C3—P1—C1—C12	172.5 (2)	C2—P1—C3—C32	−40.9 (2)
S1—P1—C1—C12	−69.7 (2)	C1—P1—C3—C32	−169.5 (2)
C3—P1—C2—C21	172.4 (2)	S1—P1—C3—C32	80.8 (2)

Symmetry codes: (i) −*x*+1, −*y*+1, −*z*; (ii) −*x*+2, −*y*, −*z*+1.

### (Bromosulfanyl)tris(propan-2-yl)phosphonium tetrabromidoaurate(III) (17b) Hydrogen-bond geometry (Å, º)

**Table:**

*D*—H···*A*	*D*—H	H···*A*	*D*···*A*	*D*—H···*A*
C21—H21*B*···Br1	0.98	3.28	3.864 (3)	120
C3—H3···Br2^iii^	1.00	3.29	3.792 (3)	113
C31—H31*C*···Br2^iii^	0.98	3.00	3.787 (3)	138
C32—H32*A*···Br2^iii^	0.98	2.98	3.763 (3)	137
C1—H1···Br4^iii^	1.00	3.15	3.997 (3)	144
C21—H21*C*···Br4^iv^	0.98	3.06	3.943 (3)	151
C31—H31*B*···Br4^iii^	0.98	3.04	3.983 (3)	162
C1—H1···Br5^iii^	1.00	2.98	3.802 (3)	140
C22—H22*C*···Br5^v^	0.98	3.11	3.911 (3)	140

Symmetry codes: (iii) *x*, *y*+1, *z*; (iv) *x*−1, *y*, *z*; (v) *x*−1, *y*+1, *z*.

### (Bromosulfanyl)(tert-butyl)bis(propan-2-yl)phosphonium tetrabromidoaurate(III) (18b) Crystal data

**Table:**

(C_10_H_23_BrPS)[AuBr_4_]	*Z* = 2
*M**_r_* = 802.83	*F*(000) = 736
Triclinic, *P*1	*D*_x_ = 2.675 Mg m^−^^3^
*a* = 7.8455 (6) Å	Mo *K*α radiation, λ = 0.71073 Å
*b* = 9.1380 (6) Å	Cell parameters from 9831 reflections
*c* = 15.5440 (9) Å	θ = 2.5–30.8°
α = 86.600 (5)°	µ = 17.57 mm^−^^1^
β = 81.097 (6)°	*T* = 100 K
γ = 64.862 (6)°	Plate, orange-red
*V* = 996.65 (13) Å^3^	0.25 × 0.1 × 0.03 mm

### (Bromosulfanyl)(tert-butyl)bis(propan-2-yl)phosphonium tetrabromidoaurate(III) (18b) Data collection

**Table:**

Oxford Diffraction Xcalibur, Eos diffractometer	5731 independent reflections
Radiation source: Enhance (Mo) X-ray Source	5030 reflections with *I* > 2σ(*I*)
Detector resolution: 16.1419 pixels mm^-1^	*R*_int_ = 0.045
ω–scan	θ_max_ = 30.8°, θ_min_ = 2.5°
Absorption correction: multi-scan (CrysAlisPro; Rigaku OD, 2015)	*h* = −11→10
*T*_min_ = 0.097, *T*_max_ = 0.621	*k* = −12→13
26487 measured reflections	*l* = −22→22

### (Bromosulfanyl)(tert-butyl)bis(propan-2-yl)phosphonium tetrabromidoaurate(III) (18b) Refinement

**Table:**

Refinement on *F*^2^	Primary atom site location: structure-invariant direct methods
Least-squares matrix: full	Secondary atom site location: difference Fourier map
*R*[*F*^2^ > 2σ(*F*^2^)] = 0.027	Hydrogen site location: inferred from neighbouring sites
*wR*(*F*^2^) = 0.052	H-atom parameters constrained
*S* = 1.06	*w* = 1/[σ^2^(*F*_o_^2^) + (0.0167*P*)^2^] where *P* = (*F*_o_^2^ + 2*F*_c_^2^)/3
5731 reflections	(Δ/σ)_max_ = 0.001
173 parameters	Δρ_max_ = 2.15 e Å^−^^3^
0 restraints	Δρ_min_ = −2.24 e Å^−^^3^

### (Bromosulfanyl)(tert-butyl)bis(propan-2-yl)phosphonium tetrabromidoaurate(III) (18b) Fractional atomic coordinates and isotropic or equivalent isotropic displacement parameters (Å^2^)

**Table:**

	*x*	*y*	*z*	*U*_iso_*/*U*_eq_	
P1	0.50339 (12)	0.10905 (11)	0.24799 (7)	0.01362 (19)	
S1	0.39290 (12)	−0.05194 (10)	0.30387 (7)	0.0183 (2)	
Br1	0.16092 (5)	0.09936 (4)	0.40317 (3)	0.01924 (8)	
C1	0.7420 (5)	−0.0279 (4)	0.1918 (3)	0.0167 (8)	
C2	0.5065 (5)	0.2376 (5)	0.3328 (3)	0.0198 (8)	
H2	0.370055	0.306364	0.355119	0.024*	
C3	0.3524 (5)	0.2400 (4)	0.1706 (3)	0.0185 (8)	
H3	0.425737	0.295673	0.135909	0.022*	
C11	0.8009 (5)	0.0614 (5)	0.1138 (3)	0.0250 (9)	
H11A	0.797239	0.162321	0.134229	0.038*	
H11B	0.712607	0.085987	0.071261	0.038*	
H11C	0.930165	−0.007171	0.086514	0.038*	
C12	0.8867 (5)	−0.0766 (5)	0.2555 (3)	0.0229 (9)	
H12A	1.010620	−0.154105	0.227133	0.034*	
H12B	0.844151	−0.126343	0.306871	0.034*	
H12C	0.898209	0.019605	0.273240	0.034*	
C13	0.7392 (5)	−0.1820 (4)	0.1585 (3)	0.0216 (8)	
H13A	0.638084	−0.152137	0.122107	0.032*	
H13B	0.715560	−0.245457	0.208142	0.032*	
H13C	0.862209	−0.246547	0.123983	0.032*	
C21	0.5961 (6)	0.1502 (5)	0.4119 (3)	0.0299 (10)	
H21A	0.734900	0.106729	0.398138	0.045*	
H21B	0.558656	0.061372	0.427490	0.045*	
H21C	0.552320	0.226140	0.460940	0.045*	
C22	0.5904 (6)	0.3550 (5)	0.2935 (3)	0.0312 (11)	
H22A	0.576068	0.432071	0.338336	0.047*	
H22B	0.523056	0.413800	0.245404	0.047*	
H22C	0.725708	0.294173	0.271683	0.047*	
C31	0.3097 (6)	0.1447 (5)	0.1057 (3)	0.0265 (9)	
H31A	0.227586	0.096195	0.136335	0.040*	
H31B	0.429052	0.059163	0.078583	0.040*	
H31C	0.245011	0.217668	0.060592	0.040*	
C32	0.1676 (5)	0.3728 (4)	0.2152 (3)	0.0231 (9)	
H32A	0.096116	0.442908	0.170905	0.035*	
H32B	0.197860	0.437057	0.253499	0.035*	
H32C	0.090581	0.323216	0.249639	0.035*	
Au1	0.000000	0.500000	0.500000	0.01491 (5)	
Br2	−0.15629 (5)	0.32924 (5)	0.55955 (3)	0.02348 (9)	
Br3	0.22769 (6)	0.38839 (5)	0.60137 (3)	0.02456 (9)	
Au2	0.500000	0.500000	0.000000	0.01379 (5)	
Br4	0.35239 (5)	0.70272 (5)	0.11380 (3)	0.02518 (10)	
Br5	0.81073 (5)	0.45559 (4)	0.03161 (3)	0.02119 (9)	

### (Bromosulfanyl)(tert-butyl)bis(propan-2-yl)phosphonium tetrabromidoaurate(III) (18b) Atomic displacement parameters (Å^2^)

**Table:**

	*U*^11^	*U*^22^	*U*^33^	*U*^12^	*U*^13^	*U*^23^
P1	0.0130 (4)	0.0145 (4)	0.0138 (5)	−0.0062 (4)	−0.0016 (4)	−0.0009 (4)
S1	0.0158 (4)	0.0154 (4)	0.0232 (6)	−0.0069 (4)	−0.0008 (4)	0.0012 (4)
Br1	0.01646 (17)	0.02354 (18)	0.0193 (2)	−0.01088 (15)	0.00037 (15)	0.00017 (16)
C1	0.0159 (17)	0.0153 (17)	0.018 (2)	−0.0055 (14)	−0.0022 (15)	0.0009 (15)
C2	0.0176 (18)	0.0248 (19)	0.019 (2)	−0.0114 (16)	0.0008 (15)	−0.0054 (16)
C3	0.0190 (18)	0.0151 (17)	0.020 (2)	−0.0055 (15)	−0.0044 (16)	0.0026 (15)
C11	0.0207 (19)	0.027 (2)	0.022 (2)	−0.0067 (17)	0.0041 (17)	0.0010 (18)
C12	0.0144 (18)	0.029 (2)	0.023 (2)	−0.0073 (16)	−0.0027 (16)	−0.0007 (18)
C13	0.0199 (19)	0.0196 (19)	0.022 (2)	−0.0053 (16)	−0.0014 (16)	−0.0038 (17)
C21	0.023 (2)	0.049 (3)	0.023 (3)	−0.019 (2)	−0.0049 (18)	−0.007 (2)
C22	0.029 (2)	0.028 (2)	0.045 (3)	−0.0203 (19)	−0.001 (2)	−0.012 (2)
C31	0.028 (2)	0.024 (2)	0.026 (3)	−0.0051 (17)	−0.0150 (18)	−0.0004 (18)
C32	0.0213 (19)	0.0183 (18)	0.024 (2)	−0.0020 (16)	−0.0059 (17)	0.0003 (17)
Au1	0.01684 (10)	0.01425 (9)	0.01507 (11)	−0.00780 (8)	−0.00204 (8)	−0.00133 (8)
Br2	0.0262 (2)	0.02399 (19)	0.0257 (2)	−0.01649 (17)	−0.00262 (17)	0.00259 (17)
Br3	0.0251 (2)	0.0284 (2)	0.0240 (2)	−0.01321 (17)	−0.00993 (17)	0.00443 (17)
Au2	0.00922 (9)	0.01233 (9)	0.01999 (12)	−0.00528 (7)	−0.00075 (7)	0.00101 (8)
Br4	0.01812 (18)	0.0244 (2)	0.0315 (3)	−0.00830 (16)	0.00282 (16)	−0.01051 (18)
Br5	0.01142 (16)	0.02067 (18)	0.0331 (3)	−0.00777 (14)	−0.00541 (15)	0.00139 (17)

### (Bromosulfanyl)(tert-butyl)bis(propan-2-yl)phosphonium tetrabromidoaurate(III) (18b) Geometric parameters (Å, º)

**Table:**

P1—C2	1.827 (4)	C13—H13B	0.9800
P1—C3	1.830 (4)	C13—H13C	0.9800
P1—C1	1.862 (4)	C21—H21A	0.9800
P1—S1	2.0902 (12)	C21—H21B	0.9800
S1—Br1	2.2028 (10)	C21—H21C	0.9800
Br1—Br2	3.2874 (6)	C22—H22A	0.9800
C1—C12	1.527 (5)	C22—H22B	0.9800
C1—C13	1.539 (5)	C22—H22C	0.9800
C1—C11	1.542 (5)	C31—H31A	0.9800
C2—C21	1.522 (6)	C31—H31B	0.9800
C2—C22	1.535 (5)	C31—H31C	0.9800
C2—H2	1.0000	C32—H32A	0.9800
C3—C31	1.532 (5)	C32—H32B	0.9800
C3—C32	1.533 (5)	C32—H32C	0.9800
C3—H3	1.0000	Au1—Br3	2.4180 (4)
C11—H11A	0.9800	Au1—Br3^i^	2.4181 (4)
C11—H11B	0.9800	Au1—Br2	2.4287 (4)
C11—H11C	0.9800	Au1—Br2^i^	2.4287 (4)
C12—H12A	0.9800	Au2—Br4	2.4154 (4)
C12—H12B	0.9800	Au2—Br4^ii^	2.4154 (4)
C12—H12C	0.9800	Au2—Br5^ii^	2.4199 (4)
C13—H13A	0.9800	Au2—Br5	2.4199 (4)
			
C2—P1—C3	107.86 (17)	C1—C13—H13C	109.5
C2—P1—C1	114.96 (17)	H13A—C13—H13C	109.5
C3—P1—C1	111.42 (17)	H13B—C13—H13C	109.5
C2—P1—S1	109.57 (13)	C2—C21—H21A	109.5
C3—P1—S1	110.03 (13)	C2—C21—H21B	109.5
C1—P1—S1	102.89 (12)	H21A—C21—H21B	109.5
P1—S1—Br1	102.51 (5)	C2—C21—H21C	109.5
S1—Br1—Br2	174.89 (3)	H21A—C21—H21C	109.5
C12—C1—C13	108.6 (3)	H21B—C21—H21C	109.5
C12—C1—C11	109.7 (3)	C2—C22—H22A	109.5
C13—C1—C11	109.0 (3)	C2—C22—H22B	109.5
C12—C1—P1	109.4 (3)	H22A—C22—H22B	109.5
C13—C1—P1	111.1 (2)	C2—C22—H22C	109.5
C11—C1—P1	109.1 (2)	H22A—C22—H22C	109.5
C21—C2—C22	112.2 (3)	H22B—C22—H22C	109.5
C21—C2—P1	115.9 (3)	C3—C31—H31A	109.5
C22—C2—P1	110.5 (3)	C3—C31—H31B	109.5
C21—C2—H2	105.8	H31A—C31—H31B	109.5
C22—C2—H2	105.8	C3—C31—H31C	109.5
P1—C2—H2	105.8	H31A—C31—H31C	109.5
C31—C3—C32	110.6 (3)	H31B—C31—H31C	109.5
C31—C3—P1	112.5 (2)	C3—C32—H32A	109.5
C32—C3—P1	112.9 (3)	C3—C32—H32B	109.5
C31—C3—H3	106.8	H32A—C32—H32B	109.5
C32—C3—H3	106.8	C3—C32—H32C	109.5
P1—C3—H3	106.8	H32A—C32—H32C	109.5
C1—C11—H11A	109.5	H32B—C32—H32C	109.5
C1—C11—H11B	109.5	Br3—Au1—Br3^i^	180.0
H11A—C11—H11B	109.5	Br3—Au1—Br2	90.373 (15)
C1—C11—H11C	109.5	Br3^i^—Au1—Br2	89.628 (15)
H11A—C11—H11C	109.5	Br3—Au1—Br2^i^	89.627 (15)
H11B—C11—H11C	109.5	Br3^i^—Au1—Br2^i^	90.372 (15)
C1—C12—H12A	109.5	Br2—Au1—Br2^i^	180.000 (11)
C1—C12—H12B	109.5	Au1—Br2—Br1	78.058 (13)
H12A—C12—H12B	109.5	Br4—Au2—Br4^ii^	180.0
C1—C12—H12C	109.5	Br4—Au2—Br5^ii^	90.027 (15)
H12A—C12—H12C	109.5	Br4^ii^—Au2—Br5^ii^	89.972 (15)
H12B—C12—H12C	109.5	Br4—Au2—Br5	89.972 (15)
C1—C13—H13A	109.5	Br4^ii^—Au2—Br5	90.029 (15)
C1—C13—H13B	109.5	Br5^ii^—Au2—Br5	180.0
H13A—C13—H13B	109.5		
			
C2—P1—S1—Br1	39.31 (14)	C3—P1—C2—C21	169.8 (3)
C3—P1—S1—Br1	−79.12 (14)	C1—P1—C2—C21	−65.3 (3)
C1—P1—S1—Br1	162.05 (13)	S1—P1—C2—C21	50.0 (3)
C2—P1—C1—C12	31.6 (3)	C3—P1—C2—C22	−61.2 (3)
C3—P1—C1—C12	154.7 (2)	C1—P1—C2—C22	63.8 (3)
S1—P1—C1—C12	−87.5 (2)	S1—P1—C2—C22	179.0 (2)
C2—P1—C1—C13	151.5 (3)	C2—P1—C3—C31	−169.6 (3)
C3—P1—C1—C13	−85.4 (3)	C1—P1—C3—C31	63.3 (3)
S1—P1—C1—C13	32.4 (3)	S1—P1—C3—C31	−50.1 (3)
C2—P1—C1—C11	−88.4 (3)	C2—P1—C3—C32	−43.6 (3)
C3—P1—C1—C11	34.7 (3)	C1—P1—C3—C32	−170.6 (3)
S1—P1—C1—C11	152.6 (2)	S1—P1—C3—C32	75.9 (3)

Symmetry codes: (i) −*x*, −*y*+1, −*z*+1; (ii) −*x*+1, −*y*+1, −*z*.

### (Bromosulfanyl)(tert-butyl)bis(propan-2-yl)phosphonium tetrabromidoaurate(III) (18b) Hydrogen-bond geometry (Å, º)

**Table:**

*D*—H···*A*	*D*—H	H···*A*	*D*···*A*	*D*—H···*A*
C13—H13*B*···S1	0.98	2.66	3.095 (4)	107
C2—H2···Br1	1.00	2.99	3.459 (4)	110
C3—H3···Au2	1.00	2.89	3.828 (4)	156
C32—H32*C*···Br1	0.98	3.01	3.739 (4)	132
C21—H21*C*···Br3	0.98	2.98	3.814 (4)	143
C11—H11*A*···Br5	0.98	3.07	3.778 (4)	130
C2—H2···Br2^i^	1.00	3.28	4.017 (4)	132
C22—H22*A*···Br3^iii^	0.98	2.95	3.771 (4)	142
C12—H12*B*···Br3^iv^	0.98	2.93	3.832 (4)	154
C13—H13*B*···Br3^iv^	0.98	3.18	4.075 (4)	152
C12—H12*A*···Br4^v^	0.98	2.80	3.747 (4)	163
C13—H13*A*···Br4^vi^	0.98	3.07	3.776 (4)	130

Symmetry codes: (i) −*x*, −*y*+1, −*z*+1; (iii) −*x*+1, −*y*+1, −*z*+1; (iv) −*x*+1, −*y*, −*z*+1; (v) *x*+1, *y*−1, *z*; (vi) *x*, *y*−1, *z*.

### (Bromosulfanyl)bis(tert-butyl)(propan-2-yl)phosphonium tetrabromidoaurate(III) (19b) Crystal data

**Table:**

(C_11_H_25_BrPS)[AuBr_4_]	*F*(000) = 1504
*M**_r_* = 816.86	*D*_x_ = 2.584 Mg m^−^^3^
Monoclinic, *P*2_1_/*n*	Mo *K*α radiation, λ = 0.71073 Å
*a* = 12.4712 (4) Å	Cell parameters from 15934 reflections
*b* = 10.3712 (3) Å	θ = 2.3–30.9°
*c* = 16.2524 (5) Å	µ = 16.68 mm^−^^1^
β = 92.724 (3)°	*T* = 100 K
*V* = 2099.73 (11) Å^3^	Plate, orange-red
*Z* = 4	0.35 × 0.25 × 0.10 mm

### (Bromosulfanyl)bis(tert-butyl)(propan-2-yl)phosphonium tetrabromidoaurate(III) (19b) Data collection

**Table:**

Oxford Diffraction Xcalibur, Eos diffractometer	6335 independent reflections
Radiation source: Enhance (Mo) X-ray Source	5041 reflections with *I* > 2σ(*I*)
Detector resolution: 16.1419 pixels mm^-1^	*R*_int_ = 0.065
ω scans	θ_max_ = 30.9°, θ_min_ = 2.3°
Absorption correction: multi-scan (CrysAlisPro; Rigaku OD, 2015)	*h* = −17→17
*T*_min_ = 0.068, *T*_max_ = 0.286	*k* = −14→14
70058 measured reflections	*l* = −23→23

### (Bromosulfanyl)bis(tert-butyl)(propan-2-yl)phosphonium tetrabromidoaurate(III) (19b) Refinement

**Table:**

Refinement on *F*^2^	Primary atom site location: structure-invariant direct methods
Least-squares matrix: full	Secondary atom site location: difference Fourier map
*R*[*F*^2^ > 2σ(*F*^2^)] = 0.033	Hydrogen site location: inferred from neighbouring sites
*wR*(*F*^2^) = 0.061	H-atom parameters constrained
*S* = 1.09	*w* = 1/[σ^2^(*F*_o_^2^) + (0.0128*P*)^2^ + 8.4095*P*] where *P* = (*F*_o_^2^ + 2*F*_c_^2^)/3
6335 reflections	(Δ/σ)_max_ < 0.001
183 parameters	Δρ_max_ = 2.42 e Å^−^^3^
0 restraints	Δρ_min_ = −1.44 e Å^−^^3^

### (Bromosulfanyl)bis(tert-butyl)(propan-2-yl)phosphonium tetrabromidoaurate(III) (19b) Fractional atomic coordinates and isotropic or equivalent isotropic displacement parameters (Å^2^)

**Table:**

	*x*	*y*	*z*	*U*_iso_*/*U*_eq_	
P1	0.47617 (8)	0.09340 (11)	0.25608 (7)	0.0122 (2)	
S1	0.44209 (9)	0.26253 (11)	0.18886 (7)	0.0194 (2)	
Br1	0.50831 (4)	0.41904 (4)	0.26809 (3)	0.02289 (10)	
C2	0.3752 (4)	0.0775 (5)	0.3358 (3)	0.0227 (10)	
C1	0.4639 (4)	−0.0278 (4)	0.1711 (3)	0.0190 (9)	
C3	0.6110 (3)	0.1033 (4)	0.3061 (3)	0.0177 (9)	
H3	0.603870	0.156065	0.356994	0.021*	
C21	0.4031 (5)	−0.0301 (6)	0.3978 (3)	0.0363 (14)	
H21A	0.346244	−0.036995	0.437141	0.054*	
H21B	0.471370	−0.010183	0.427380	0.054*	
H21C	0.409507	−0.112108	0.368404	0.054*	
C22	0.2627 (4)	0.0542 (5)	0.2959 (3)	0.0308 (12)	
H22A	0.260412	−0.030698	0.269459	0.046*	
H22B	0.246401	0.121028	0.254587	0.046*	
H22C	0.209473	0.057430	0.338374	0.046*	
C23	0.3721 (4)	0.2056 (5)	0.3835 (3)	0.0333 (13)	
H23A	0.327063	0.195525	0.430947	0.050*	
H23B	0.342049	0.273327	0.347238	0.050*	
H23C	0.445015	0.229503	0.402811	0.050*	
C11	0.5660 (4)	−0.0216 (5)	0.1213 (3)	0.0284 (11)	
H11A	0.558092	−0.079602	0.073841	0.043*	
H11B	0.628200	−0.047990	0.156406	0.043*	
H11C	0.576469	0.066881	0.102076	0.043*	
C12	0.4520 (4)	−0.1653 (4)	0.2062 (3)	0.0251 (11)	
H12A	0.385209	−0.170931	0.235450	0.038*	
H12B	0.513072	−0.184233	0.244456	0.038*	
H12C	0.450086	−0.227835	0.160975	0.038*	
C13	0.3676 (4)	0.0033 (5)	0.1120 (3)	0.0285 (12)	
H13A	0.379906	0.085701	0.084545	0.043*	
H13B	0.302400	0.009192	0.143225	0.043*	
H13C	0.358977	−0.065155	0.070692	0.043*	
C31	0.6958 (3)	0.1712 (5)	0.2556 (3)	0.0249 (11)	
H31A	0.757212	0.195668	0.292127	0.037*	
H31B	0.664318	0.248634	0.229716	0.037*	
H31C	0.719712	0.112565	0.212930	0.037*	
C32	0.6555 (4)	−0.0289 (5)	0.3348 (4)	0.0298 (12)	
H32A	0.669637	−0.081990	0.286622	0.045*	
H32B	0.602792	−0.072220	0.368041	0.045*	
H32C	0.722404	−0.016356	0.367956	0.045*	
Au1	0.500000	0.500000	0.500000	0.01294 (5)	
Br2	0.59615 (4)	0.63077 (5)	0.40419 (3)	0.02733 (11)	
Br3	0.64240 (4)	0.34181 (5)	0.49969 (3)	0.02734 (12)	
Au2	0.500000	0.500000	0.000000	0.01160 (5)	
Br4	0.62972 (3)	0.32712 (4)	0.01765 (3)	0.01734 (9)	
Br5	0.35771 (3)	0.34321 (4)	−0.02188 (3)	0.01984 (10)	

### (Bromosulfanyl)bis(tert-butyl)(propan-2-yl)phosphonium tetrabromidoaurate(III) (19b) Atomic displacement parameters (Å^2^)

**Table:**

	*U*^11^	*U*^22^	*U*^33^	*U*^12^	*U*^13^	*U*^23^
P1	0.0133 (5)	0.0126 (5)	0.0109 (5)	0.0000 (4)	0.0005 (4)	−0.0006 (4)
S1	0.0249 (6)	0.0143 (5)	0.0185 (6)	0.0028 (4)	−0.0035 (4)	0.0004 (4)
Br1	0.0262 (2)	0.0137 (2)	0.0285 (3)	0.00032 (18)	−0.00035 (19)	−0.00333 (19)
C2	0.023 (2)	0.028 (3)	0.018 (2)	−0.009 (2)	0.0084 (18)	−0.006 (2)
C1	0.034 (3)	0.011 (2)	0.012 (2)	0.0021 (18)	−0.0016 (18)	−0.0018 (17)
C3	0.017 (2)	0.018 (2)	0.018 (2)	−0.0013 (17)	−0.0034 (17)	0.0002 (18)
C21	0.041 (3)	0.047 (4)	0.022 (3)	−0.012 (3)	0.009 (2)	0.006 (3)
C22	0.019 (2)	0.035 (3)	0.039 (3)	−0.009 (2)	0.006 (2)	−0.013 (3)
C23	0.030 (3)	0.038 (3)	0.034 (3)	−0.014 (2)	0.018 (2)	−0.018 (3)
C11	0.039 (3)	0.023 (3)	0.024 (3)	0.005 (2)	0.009 (2)	−0.003 (2)
C12	0.038 (3)	0.013 (2)	0.024 (3)	−0.005 (2)	−0.004 (2)	−0.0010 (19)
C13	0.044 (3)	0.022 (3)	0.018 (2)	0.003 (2)	−0.013 (2)	−0.003 (2)
C31	0.013 (2)	0.027 (3)	0.035 (3)	−0.0022 (19)	0.0040 (19)	0.002 (2)
C32	0.025 (3)	0.021 (2)	0.042 (3)	0.001 (2)	−0.012 (2)	0.009 (2)
Au1	0.01148 (10)	0.01165 (11)	0.01575 (11)	0.00025 (8)	0.00140 (8)	−0.00279 (9)
Br2	0.0386 (3)	0.0202 (2)	0.0243 (2)	−0.0096 (2)	0.0132 (2)	−0.0023 (2)
Br3	0.0193 (2)	0.0219 (2)	0.0403 (3)	0.00802 (19)	−0.0039 (2)	−0.0060 (2)
Au2	0.01165 (10)	0.01152 (10)	0.01178 (10)	−0.00266 (8)	0.00220 (8)	−0.00080 (9)
Br4	0.0155 (2)	0.0148 (2)	0.0219 (2)	0.00024 (16)	0.00228 (16)	−0.00118 (18)
Br5	0.0155 (2)	0.0149 (2)	0.0287 (3)	−0.00572 (16)	−0.00267 (17)	0.00139 (18)

### (Bromosulfanyl)bis(tert-butyl)(propan-2-yl)phosphonium tetrabromidoaurate(III) (19b) Geometric parameters (Å, º)

**Table:**

P1—C3	1.836 (4)	C11—H11A	0.9800
P1—C2	1.856 (5)	C11—H11B	0.9800
P1—C1	1.868 (4)	C11—H11C	0.9800
P1—S1	2.0992 (16)	C12—H12A	0.9800
S1—Br1	2.2077 (12)	C12—H12B	0.9800
Br1—Br2	3.2686 (7)	C12—H12C	0.9800
C2—C21	1.532 (7)	C13—H13A	0.9800
C2—C22	1.537 (6)	C13—H13B	0.9800
C2—C23	1.540 (7)	C13—H13C	0.9800
C1—C13	1.537 (6)	C31—H31A	0.9800
C1—C11	1.541 (7)	C31—H31B	0.9800
C1—C12	1.545 (6)	C31—H31C	0.9800
C3—C31	1.538 (6)	C32—H32A	0.9800
C3—C32	1.543 (6)	C32—H32B	0.9800
C3—H3	1.0000	C32—H32C	0.9800
C21—H21A	0.9800	Au1—Br3^i^	2.4179 (5)
C21—H21B	0.9800	Au1—Br3	2.4179 (5)
C21—H21C	0.9800	Au1—Br2^i^	2.4247 (5)
C22—H22A	0.9800	Au1—Br2	2.4247 (5)
C22—H22B	0.9800	Au2—Br5^ii^	2.4206 (4)
C22—H22C	0.9800	Au2—Br5	2.4207 (4)
C23—H23A	0.9800	Au2—Br4	2.4228 (4)
C23—H23B	0.9800	Au2—Br4^ii^	2.4229 (4)
C23—H23C	0.9800		
			
C3—P1—C2	109.5 (2)	C1—C11—H11A	109.5
C3—P1—C1	113.9 (2)	C1—C11—H11B	109.5
C2—P1—C1	115.0 (2)	H11A—C11—H11B	109.5
C3—P1—S1	110.01 (15)	C1—C11—H11C	109.5
C2—P1—S1	108.07 (17)	H11A—C11—H11C	109.5
C1—P1—S1	99.81 (14)	H11B—C11—H11C	109.5
P1—S1—Br1	104.48 (6)	C1—C12—H12A	109.5
S1—Br1—Br2	173.07 (4)	C1—C12—H12B	109.5
C21—C2—C22	109.7 (4)	H12A—C12—H12B	109.5
C21—C2—C23	108.0 (4)	C1—C12—H12C	109.5
C22—C2—C23	107.6 (4)	H12A—C12—H12C	109.5
C21—C2—P1	112.5 (4)	H12B—C12—H12C	109.5
C22—C2—P1	110.8 (3)	C1—C13—H13A	109.5
C23—C2—P1	108.0 (3)	C1—C13—H13B	109.5
C13—C1—C11	107.7 (4)	H13A—C13—H13B	109.5
C13—C1—C12	109.8 (4)	C1—C13—H13C	109.5
C11—C1—C12	109.2 (4)	H13A—C13—H13C	109.5
C13—C1—P1	110.8 (3)	H13B—C13—H13C	109.5
C11—C1—P1	108.6 (3)	C3—C31—H31A	109.5
C12—C1—P1	110.7 (3)	C3—C31—H31B	109.5
C31—C3—C32	108.8 (4)	H31A—C31—H31B	109.5
C31—C3—P1	115.3 (3)	C3—C31—H31C	109.5
C32—C3—P1	113.2 (3)	H31A—C31—H31C	109.5
C31—C3—H3	106.3	H31B—C31—H31C	109.5
C32—C3—H3	106.3	C3—C32—H32A	109.5
P1—C3—H3	106.3	C3—C32—H32B	109.5
C2—C21—H21A	109.5	H32A—C32—H32B	109.5
C2—C21—H21B	109.5	C3—C32—H32C	109.5
H21A—C21—H21B	109.5	H32A—C32—H32C	109.5
C2—C21—H21C	109.5	H32B—C32—H32C	109.5
H21A—C21—H21C	109.5	Br3^i^—Au1—Br3	180.0
H21B—C21—H21C	109.5	Br3^i^—Au1—Br2^i^	89.572 (19)
C2—C22—H22A	109.5	Br3—Au1—Br2^i^	90.429 (19)
C2—C22—H22B	109.5	Br3^i^—Au1—Br2	90.428 (19)
H22A—C22—H22B	109.5	Br3—Au1—Br2	89.571 (19)
C2—C22—H22C	109.5	Br2^i^—Au1—Br2	180.0
H22A—C22—H22C	109.5	Au1—Br2—Br1	84.190 (16)
H22B—C22—H22C	109.5	Br5^ii^—Au2—Br5	180.0
C2—C23—H23A	109.5	Br5^ii^—Au2—Br4	89.945 (15)
C2—C23—H23B	109.5	Br5—Au2—Br4	90.055 (15)
H23A—C23—H23B	109.5	Br5^ii^—Au2—Br4^ii^	90.055 (15)
C2—C23—H23C	109.5	Br5—Au2—Br4^ii^	89.944 (15)
H23A—C23—H23C	109.5	Br4—Au2—Br4^ii^	180.0
H23B—C23—H23C	109.5		
			
C3—P1—S1—Br1	−40.27 (17)	S1—P1—C1—C13	−41.1 (4)
C2—P1—S1—Br1	79.21 (16)	C3—P1—C1—C11	−40.2 (4)
C1—P1—S1—Br1	−160.33 (16)	C2—P1—C1—C11	−167.7 (3)
C3—P1—C2—C21	−50.6 (4)	S1—P1—C1—C11	76.9 (3)
C1—P1—C2—C21	79.1 (4)	C3—P1—C1—C12	79.7 (4)
S1—P1—C2—C21	−170.5 (3)	C2—P1—C1—C12	−47.8 (4)
C3—P1—C2—C22	−173.8 (3)	S1—P1—C1—C12	−163.1 (3)
C1—P1—C2—C22	−44.1 (4)	C2—P1—C3—C31	−155.5 (3)
S1—P1—C2—C22	66.3 (4)	C1—P1—C3—C31	74.2 (4)
C3—P1—C2—C23	68.5 (4)	S1—P1—C3—C31	−36.9 (4)
C1—P1—C2—C23	−161.8 (3)	C2—P1—C3—C32	78.3 (4)
S1—P1—C2—C23	−51.4 (4)	C1—P1—C3—C32	−51.9 (4)
C3—P1—C1—C13	−158.3 (3)	S1—P1—C3—C32	−163.0 (3)
C2—P1—C1—C13	74.2 (4)		

Symmetry codes: (i) −*x*+1, −*y*+1, −*z*+1; (ii) −*x*+1, −*y*+1, −*z*.

### (Bromosulfanyl)bis(tert-butyl)(propan-2-yl)phosphonium tetrabromidoaurate(III) (19b) Hydrogen-bond geometry (Å, º)

**Table:**

*D*—H···*A*	*D*—H	H···*A*	*D*···*A*	*D*—H···*A*
C13—H13*A*···S1	0.98	2.59	3.089 (5)	112
C31—H31*B*···Br1	0.98	2.72	3.487 (5)	135
C23—H23*B*···Br1	0.98	2.91	3.407 (6)	112
C3—H3···Br3	1.00	3.04	4.007 (5)	164
C23—H23*C*···Br3	0.98	3.09	4.040 (5)	165
C11—H11*C*···Br4	0.98	3.11	4.084 (5)	171
C31—H31*C*···Br2^iii^	0.98	3.06	3.781 (5)	132
C23—H23*A*···Br4^iv^	0.98	2.90	3.824 (5)	157
C13—H13*C*···Br4^v^	0.98	3.08	4.024 (5)	162
C32—H32*C*···Br4^iii^	0.98	3.03	3.814 (5)	138
C11—H11*A*···Br5^v^	0.98	3.06	3.846 (5)	138
C32—H32*C*···Br5^vi^	0.98	3.00	3.864 (5)	148

Symmetry codes: (iii) −*x*+3/2, *y*−1/2, −*z*+1/2; (iv) *x*−1/2, −*y*+1/2, *z*+1/2; (v) −*x*+1, −*y*, −*z*; (vi) *x*+1/2, −*y*+1/2, *z*+1/2.

### (Bromosulfanyl)tris(tert-butyl)phosphonium tetrabromidoaurate(III) (20b) Crystal data

**Table:**

(C_12_H_27_BrPS)[AuBr_4_]	*Z* = 2
*M**_r_* = 830.88	*F*(000) = 768
Triclinic, *P*1	*D*_x_ = 2.540 Mg m^−^^3^
*a* = 10.2218 (5) Å	Mo *K*α radiation, λ = 0.71073 Å
*b* = 10.8085 (6) Å	Cell parameters from 12650 reflections
*c* = 11.1163 (5) Å	θ = 2.5–29.6°
α = 70.909 (4)°	µ = 16.13 mm^−^^1^
β = 71.516 (5)°	*T* = 100 K
γ = 75.645 (4)°	Plate, red
*V* = 1086.40 (10) Å^3^	0.1 × 0.05 × 0.002 mm

### (Bromosulfanyl)tris(tert-butyl)phosphonium tetrabromidoaurate(III) (20b) Data collection

**Table:**

Oxford Diffraction Xcalibur, Eos diffractometer	6283 independent reflections
Radiation source: Enhance (Mo) X-ray Source	5151 reflections with *I* > 2σ(*I*)
Detector resolution: 16.1419 pixels mm^-1^	*R*_int_ = 0.078
ω scan	θ_max_ = 30.0°, θ_min_ = 2.4°
Absorption correction: multi-scan (CrysAlisPro; Rigaku OD, 2015)	*h* = −14→14
*T*_min_ = 0.284, *T*_max_ = 1.000	*k* = −15→15
73343 measured reflections	*l* = −15→15

### (Bromosulfanyl)tris(tert-butyl)phosphonium tetrabromidoaurate(III) (20b) Refinement

**Table:**

Refinement on *F*^2^	Primary atom site location: structure-invariant direct methods
Least-squares matrix: full	Secondary atom site location: difference Fourier map
*R*[*F*^2^ > 2σ(*F*^2^)] = 0.032	Hydrogen site location: inferred from neighbouring sites
*wR*(*F*^2^) = 0.066	H-atom parameters constrained
*S* = 1.05	*w* = 1/[σ^2^(*F*_o_^2^) + (0.022*P*)^2^ + 3.2663*P*] where *P* = (*F*_o_^2^ + 2*F*_c_^2^)/3
6283 reflections	(Δ/σ)_max_ = 0.001
193 parameters	Δρ_max_ = 1.56 e Å^−^^3^
0 restraints	Δρ_min_ = −1.67 e Å^−^^3^

### (Bromosulfanyl)tris(tert-butyl)phosphonium tetrabromidoaurate(III) (20b) Fractional atomic coordinates and isotropic or equivalent isotropic displacement parameters (Å^2^)

**Table:**

	*x*	*y*	*z*	*U*_iso_*/*U*_eq_	
Br1	0.59391 (5)	0.25831 (5)	0.21953 (5)	0.02502 (11)	
S1	0.37937 (12)	0.36541 (12)	0.26091 (12)	0.0198 (2)	
P1	0.25702 (12)	0.25589 (11)	0.22831 (11)	0.0129 (2)	
C1	0.0812 (5)	0.3286 (5)	0.3233 (5)	0.0188 (10)	
C2	0.3046 (5)	0.0719 (4)	0.3011 (5)	0.0179 (9)	
C3	0.2756 (5)	0.3022 (5)	0.0457 (5)	0.0192 (10)	
C11	−0.0398 (5)	0.3020 (5)	0.2854 (5)	0.0219 (10)	
H11A	−0.129199	0.337699	0.338052	0.033*	
H11B	−0.034135	0.206245	0.302280	0.033*	
H11C	−0.032962	0.345254	0.191639	0.033*	
C12	0.0707 (6)	0.4787 (5)	0.2971 (6)	0.0309 (13)	
H12A	−0.023234	0.515415	0.341352	0.046*	
H12B	0.088708	0.520700	0.202069	0.046*	
H12C	0.139943	0.496006	0.331148	0.046*	
C13	0.0652 (5)	0.2691 (6)	0.4720 (5)	0.0259 (11)	
H13A	0.142733	0.285298	0.495774	0.039*	
H13B	0.066141	0.173361	0.494761	0.039*	
H13C	−0.023518	0.310690	0.520411	0.039*	
C21	0.1768 (5)	0.0050 (5)	0.3309 (5)	0.0226 (10)	
H21A	0.201012	−0.090907	0.367185	0.034*	
H21B	0.149103	0.022308	0.249514	0.034*	
H21C	0.099153	0.040962	0.395162	0.034*	
C22	0.3481 (5)	0.0441 (5)	0.4286 (5)	0.0264 (11)	
H22A	0.271080	0.081219	0.492458	0.040*	
H22B	0.430415	0.085125	0.409510	0.040*	
H22C	0.370600	−0.051945	0.465301	0.040*	
C23	0.4267 (5)	0.0064 (5)	0.2063 (5)	0.0224 (10)	
H23A	0.508419	0.049850	0.182407	0.034*	
H23B	0.399175	0.015713	0.126606	0.034*	
H23C	0.450133	−0.087930	0.249799	0.034*	
C31	0.2029 (6)	0.2133 (6)	0.0115 (5)	0.0279 (12)	
H31A	0.104491	0.219770	0.061381	0.042*	
H31B	0.249217	0.121068	0.034629	0.042*	
H31C	0.208807	0.242862	−0.083038	0.042*	
C32	0.2122 (6)	0.4465 (5)	−0.0025 (5)	0.0303 (12)	
H32A	0.251143	0.502660	0.026493	0.045*	
H32B	0.110589	0.457207	0.033881	0.045*	
H32C	0.233973	0.472570	−0.098945	0.045*	
C33	0.4314 (5)	0.2889 (6)	−0.0280 (5)	0.0262 (11)	
H33A	0.440886	0.306298	−0.122386	0.039*	
H33B	0.478203	0.198913	0.005595	0.039*	
H33C	0.474297	0.353147	−0.014286	0.039*	
Au1	1.000000	0.000000	0.000000	0.01454 (6)	
Br2	0.84988 (5)	−0.00379 (5)	0.21819 (5)	0.02579 (12)	
Br3	1.16132 (5)	−0.18362 (5)	0.09502 (5)	0.02498 (11)	
Au2	0.500000	0.500000	0.500000	0.01325 (6)	
Br4	0.33104 (5)	0.64083 (5)	0.38216 (5)	0.01821 (10)	
Br5	0.66249 (5)	0.65479 (5)	0.37637 (5)	0.02208 (11)	

### (Bromosulfanyl)tris(tert-butyl)phosphonium tetrabromidoaurate(III) (20b) Atomic displacement parameters (Å^2^)

**Table:**

	*U*^11^	*U*^22^	*U*^33^	*U*^12^	*U*^13^	*U*^23^
Br1	0.0131 (2)	0.0355 (3)	0.0279 (3)	−0.0051 (2)	−0.00249 (19)	−0.0123 (2)
S1	0.0158 (5)	0.0232 (6)	0.0250 (6)	−0.0044 (4)	−0.0029 (5)	−0.0136 (5)
P1	0.0119 (5)	0.0133 (5)	0.0146 (6)	−0.0015 (4)	−0.0029 (4)	−0.0063 (5)
C1	0.016 (2)	0.018 (2)	0.023 (2)	−0.0006 (18)	−0.0034 (19)	−0.0105 (19)
C2	0.016 (2)	0.015 (2)	0.020 (2)	−0.0020 (17)	−0.0024 (18)	−0.0027 (18)
C3	0.021 (2)	0.022 (2)	0.015 (2)	−0.0061 (19)	−0.0047 (18)	−0.0031 (19)
C11	0.015 (2)	0.023 (2)	0.027 (3)	0.0004 (19)	−0.0067 (19)	−0.007 (2)
C12	0.023 (3)	0.023 (3)	0.050 (4)	0.002 (2)	−0.004 (2)	−0.023 (3)
C13	0.018 (2)	0.043 (3)	0.021 (2)	−0.003 (2)	−0.0013 (19)	−0.019 (2)
C21	0.019 (2)	0.017 (2)	0.029 (3)	−0.0045 (19)	−0.001 (2)	−0.007 (2)
C22	0.023 (3)	0.031 (3)	0.020 (2)	−0.003 (2)	−0.010 (2)	0.003 (2)
C23	0.018 (2)	0.013 (2)	0.031 (3)	0.0003 (18)	−0.002 (2)	−0.006 (2)
C31	0.029 (3)	0.042 (3)	0.022 (3)	−0.017 (2)	−0.006 (2)	−0.013 (2)
C32	0.031 (3)	0.028 (3)	0.026 (3)	−0.009 (2)	−0.013 (2)	0.009 (2)
C33	0.028 (3)	0.040 (3)	0.014 (2)	−0.012 (2)	−0.001 (2)	−0.010 (2)
Au1	0.01328 (12)	0.01668 (12)	0.01528 (12)	−0.00202 (9)	−0.00327 (9)	−0.00712 (9)
Br2	0.0187 (2)	0.0374 (3)	0.0170 (2)	0.0015 (2)	−0.00159 (18)	−0.0091 (2)
Br3	0.0238 (3)	0.0246 (2)	0.0239 (2)	0.00549 (19)	−0.0082 (2)	−0.0081 (2)
Au2	0.01221 (11)	0.01496 (12)	0.01321 (11)	−0.00114 (9)	−0.00191 (9)	−0.00671 (9)
Br4	0.0150 (2)	0.0181 (2)	0.0208 (2)	0.00057 (17)	−0.00566 (17)	−0.00558 (18)
Br5	0.0208 (2)	0.0268 (2)	0.0195 (2)	−0.01088 (19)	−0.00610 (18)	−0.00133 (19)

### (Bromosulfanyl)tris(tert-butyl)phosphonium tetrabromidoaurate(III) (20b) Geometric parameters (Å, º)

**Table:**

S1—P1	2.0973 (16)	C21—H21B	0.9800
Br1—S1	2.1934 (13)	C21—H21C	0.9800
Br1—Br2	3.3465 (7)	C22—H22A	0.9800
P1—C3	1.883 (5)	C22—H22B	0.9800
P1—C2	1.888 (5)	C22—H22C	0.9800
P1—C1	1.898 (5)	C23—H23A	0.9800
C1—C12	1.534 (7)	C23—H23B	0.9800
C1—C13	1.535 (7)	C23—H23C	0.9800
C1—C11	1.542 (7)	C31—H31A	0.9800
C2—C22	1.534 (7)	C31—H31B	0.9800
C2—C21	1.541 (6)	C31—H31C	0.9800
C2—C23	1.544 (6)	C32—H32A	0.9800
C3—C32	1.520 (7)	C32—H32B	0.9800
C3—C33	1.537 (7)	C32—H32C	0.9800
C3—C31	1.546 (7)	C33—H33A	0.9800
C11—H11A	0.9800	C33—H33B	0.9800
C11—H11B	0.9800	C33—H33C	0.9800
C11—H11C	0.9800	Au1—Br2	2.4178 (5)
C12—H12A	0.9800	Au1—Br2^i^	2.4178 (5)
C12—H12B	0.9800	Au1—Br3	2.4257 (5)
C12—H12C	0.9800	Au1—Br3^i^	2.4257 (5)
C13—H13A	0.9800	Au2—Br4^ii^	2.4171 (5)
C13—H13B	0.9800	Au2—Br4	2.4171 (5)
C13—H13C	0.9800	Au2—Br5	2.4234 (5)
C21—H21A	0.9800	Au2—Br5^ii^	2.4234 (5)
			
S1—Br1—Br2	157.33 (4)	C2—C21—H21C	109.5
P1—S1—Br1	105.70 (6)	H21A—C21—H21C	109.5
C3—P1—C2	113.0 (2)	H21B—C21—H21C	109.5
C3—P1—C1	113.2 (2)	C2—C22—H22A	109.5
C2—P1—C1	112.6 (2)	C2—C22—H22B	109.5
C3—P1—S1	107.74 (16)	H22A—C22—H22B	109.5
C2—P1—S1	111.85 (16)	C2—C22—H22C	109.5
C1—P1—S1	97.27 (15)	H22A—C22—H22C	109.5
C12—C1—C13	106.2 (4)	H22B—C22—H22C	109.5
C12—C1—C11	109.0 (4)	C2—C23—H23A	109.5
C13—C1—C11	109.6 (4)	C2—C23—H23B	109.5
C12—C1—P1	110.8 (3)	H23A—C23—H23B	109.5
C13—C1—P1	110.0 (3)	C2—C23—H23C	109.5
C11—C1—P1	111.2 (3)	H23A—C23—H23C	109.5
C22—C2—C21	109.3 (4)	H23B—C23—H23C	109.5
C22—C2—C23	107.4 (4)	C3—C31—H31A	109.5
C21—C2—C23	107.7 (4)	C3—C31—H31B	109.5
C22—C2—P1	111.0 (3)	H31A—C31—H31B	109.5
C21—C2—P1	108.8 (3)	C3—C31—H31C	109.5
C23—C2—P1	112.6 (3)	H31A—C31—H31C	109.5
C32—C3—C33	106.8 (4)	H31B—C31—H31C	109.5
C32—C3—C31	109.0 (4)	C3—C32—H32A	109.5
C33—C3—C31	109.9 (4)	C3—C32—H32B	109.5
C32—C3—P1	110.4 (4)	H32A—C32—H32B	109.5
C33—C3—P1	109.6 (3)	C3—C32—H32C	109.5
C31—C3—P1	111.0 (3)	H32A—C32—H32C	109.5
C1—C11—H11A	109.5	H32B—C32—H32C	109.5
C1—C11—H11B	109.5	C3—C33—H33A	109.5
H11A—C11—H11B	109.5	C3—C33—H33B	109.5
C1—C11—H11C	109.5	H33A—C33—H33B	109.5
H11A—C11—H11C	109.5	C3—C33—H33C	109.5
H11B—C11—H11C	109.5	H33A—C33—H33C	109.5
C1—C12—H12A	109.5	H33B—C33—H33C	109.5
C1—C12—H12B	109.5	Br2—Au1—Br2^i^	180.0
H12A—C12—H12B	109.5	Br2—Au1—Br3	89.836 (18)
C1—C12—H12C	109.5	Br2^i^—Au1—Br3	90.164 (18)
H12A—C12—H12C	109.5	Br2—Au1—Br3^i^	90.164 (18)
H12B—C12—H12C	109.5	Br2^i^—Au1—Br3^i^	89.836 (18)
C1—C13—H13A	109.5	Br3—Au1—Br3^i^	180.00 (2)
C1—C13—H13B	109.5	Au1—Br2—Br1	112.62 (2)
H13A—C13—H13B	109.5	Br4^ii^—Au2—Br4	180.0
C1—C13—H13C	109.5	Br4^ii^—Au2—Br5	90.493 (17)
H13A—C13—H13C	109.5	Br4—Au2—Br5	89.507 (17)
H13B—C13—H13C	109.5	Br4^ii^—Au2—Br5^ii^	89.507 (17)
C2—C21—H21A	109.5	Br4—Au2—Br5^ii^	90.493 (17)
C2—C21—H21B	109.5	Br5—Au2—Br5^ii^	180.0
H21A—C21—H21B	109.5		
			
Br2—Br1—S1—P1	−39.96 (14)	C3—P1—C2—C21	−83.6 (4)
Br1—S1—P1—C3	−81.14 (17)	C1—P1—C2—C21	46.2 (4)
Br1—S1—P1—C2	43.65 (17)	S1—P1—C2—C21	154.5 (3)
Br1—S1—P1—C1	161.63 (16)	C3—P1—C2—C23	35.6 (4)
C3—P1—C1—C12	−71.8 (4)	C1—P1—C2—C23	165.5 (3)
C2—P1—C1—C12	158.5 (4)	S1—P1—C2—C23	−86.2 (3)
S1—P1—C1—C12	41.1 (4)	C2—P1—C3—C32	170.1 (3)
C3—P1—C1—C13	171.1 (3)	C1—P1—C3—C32	40.5 (4)
C2—P1—C1—C13	41.3 (4)	S1—P1—C3—C32	−65.9 (4)
S1—P1—C1—C13	−76.0 (3)	C2—P1—C3—C33	−72.6 (4)
C3—P1—C1—C11	49.5 (4)	C1—P1—C3—C33	157.9 (3)
C2—P1—C1—C11	−80.2 (4)	S1—P1—C3—C33	51.5 (4)
S1—P1—C1—C11	162.4 (3)	C2—P1—C3—C31	49.0 (4)
C3—P1—C2—C22	156.0 (3)	C1—P1—C3—C31	−80.5 (4)
C1—P1—C2—C22	−74.2 (4)	S1—P1—C3—C31	173.1 (3)
S1—P1—C2—C22	34.2 (4)		

Symmetry codes: (i) −*x*+2, −*y*, −*z*; (ii) −*x*+1, −*y*+1, −*z*+1.

### (Bromosulfanyl)tris(tert-butyl)phosphonium tetrabromidoaurate(III) (20b) Hydrogen-bond geometry (Å, º)

**Table:**

*D*—H···*A*	*D*—H	H···*A*	*D*···*A*	*D*—H···*A*
C12—H12*C*···S1	0.98	2.53	3.036 (5)	112
C22—H22*B*···Br1	0.98	2.74	3.569 (5)	143
C23—H23*A*···Br1	0.98	2.80	3.617 (5)	142
C22—H22*A*···Br2^iii^	0.98	2.97	3.739 (5)	137
C13—H13*C*···Br4^iv^	0.98	2.95	3.859 (5)	154
C21—H21*A*···Br4^v^	0.98	2.85	3.785 (5)	160

Symmetry codes: (iii) −*x*+1, −*y*, −*z*+1; (iv) −*x*, −*y*+1, −*z*+1; (v) *x*, *y*−1, *z*.

### (Bromoselanyl)tris(propan-2-yl)phosphonium tetrabromidoaurate(III) (21b) Crystal data

**Table:**

(C_9_H_21_BrPSe)[AuBr_4_]	*Z* = 2
*M**_r_* = 835.70	*F*(000) = 756
Triclinic, *P*1	*D*_x_ = 2.944 Mg m^−^^3^
*a* = 7.9212 (4) Å	Mo *K*α radiation, λ = 0.71073 Å
*b* = 8.0606 (4) Å	Cell parameters from 16838 reflections
*c* = 15.2911 (8) Å	θ = 2.6–30.8°
α = 76.817 (5)°	µ = 20.39 mm^−^^1^
β = 82.668 (5)°	*T* = 100 K
γ = 87.728 (4)°	Plate, red
*V* = 942.78 (9) Å^3^	0.1 × 0.1 × 0.05 mm

### (Bromoselanyl)tris(propan-2-yl)phosphonium tetrabromidoaurate(III) (21b) Data collection

**Table:**

Oxford Diffraction Xcalibur, Eos diffractometer	5661 independent reflections
Radiation source: Enhance (Mo) X-ray Source	4881 reflections with *I* > 2σ(*I*)
Detector resolution: 16.1419 pixels mm^-1^	*R*_int_ = 0.057
ω scan	θ_max_ = 30.9°, θ_min_ = 2.6°
Absorption correction: multi-scan (CrysAlisPro; Rigaku OD, 2015)	*h* = −11→11
*T*_min_ = 0.434, *T*_max_ = 1.000	*k* = −11→11
69642 measured reflections	*l* = −22→22

### (Bromoselanyl)tris(propan-2-yl)phosphonium tetrabromidoaurate(III) (21b) Refinement

**Table:**

Refinement on *F*^2^	Secondary atom site location: difference Fourier map
Least-squares matrix: full	Hydrogen site location: inferred from neighbouring sites
*R*[*F*^2^ > 2σ(*F*^2^)] = 0.024	H-atom parameters constrained
*wR*(*F*^2^) = 0.045	*w* = 1/[σ^2^(*F*_o_^2^) + (0.0173*P*)^2^ + 0.8539*P*] where *P* = (*F*_o_^2^ + 2*F*_c_^2^)/3
*S* = 1.06	(Δ/σ)_max_ = 0.001
5661 reflections	Δρ_max_ = 1.53 e Å^−^^3^
164 parameters	Δρ_min_ = −1.01 e Å^−^^3^
0 restraints	Extinction correction: *SHELXL2019/3* (Sheldrick, 2015), *F*_c_^*^ = *kF*_c_[1 + 0.001 *F*_c_^2^λ^3^/sin(2θ)]^-1/4^
Primary atom site location: structure-invariant direct methods	Extinction coefficient: 0.00116 (7)

### (Bromoselanyl)tris(propan-2-yl)phosphonium tetrabromidoaurate(III) (21b) Fractional atomic coordinates and isotropic or equivalent isotropic displacement parameters (Å^2^)

**Table:**

	*x*	*y*	*z*	*U*_iso_*/*U*_eq_	
P1	0.69684 (11)	0.75293 (10)	0.25729 (6)	0.01015 (16)	
Se1	0.89396 (4)	0.54763 (4)	0.28517 (2)	0.01562 (8)	
Br1	0.85395 (5)	0.42244 (4)	0.16722 (2)	0.01794 (8)	
C1	0.7009 (4)	0.8501 (4)	0.3543 (2)	0.0149 (7)	
H1	0.823646	0.866198	0.358989	0.018*	
C2	0.4967 (4)	0.6579 (4)	0.2481 (2)	0.0124 (6)	
H2	0.515433	0.614546	0.191261	0.015*	
C3	0.7589 (4)	0.9128 (4)	0.1533 (2)	0.0124 (6)	
H3	0.684416	1.015095	0.156099	0.015*	
C11	0.6179 (5)	1.0273 (4)	0.3422 (3)	0.0203 (8)	
H11A	0.496182	1.018268	0.338242	0.031*	
H11B	0.671710	1.101572	0.286496	0.031*	
H11C	0.632581	1.075411	0.394026	0.031*	
C12	0.6281 (5)	0.7327 (5)	0.4448 (2)	0.0199 (7)	
H12A	0.662665	0.774416	0.495204	0.030*	
H12B	0.671987	0.616491	0.447476	0.030*	
H12C	0.503620	0.732766	0.449240	0.030*	
C21	0.4418 (5)	0.5047 (4)	0.3243 (3)	0.0187 (7)	
H21A	0.404408	0.543359	0.380078	0.028*	
H21B	0.538022	0.425173	0.333956	0.028*	
H21C	0.347723	0.447235	0.307976	0.028*	
C22	0.3559 (5)	0.7941 (4)	0.2344 (3)	0.0189 (7)	
H22A	0.253104	0.743321	0.222620	0.028*	
H22B	0.393905	0.886888	0.182871	0.028*	
H22C	0.330599	0.839065	0.289114	0.028*	
C31	0.9421 (4)	0.9693 (4)	0.1496 (2)	0.0164 (7)	
H31A	1.020134	0.873840	0.143993	0.025*	
H31B	0.955442	1.005973	0.205176	0.025*	
H31C	0.968212	1.064298	0.097271	0.025*	
C32	0.7299 (5)	0.8572 (4)	0.0675 (2)	0.0181 (7)	
H32A	0.754886	0.952060	0.014995	0.027*	
H32B	0.611020	0.823421	0.071968	0.027*	
H32C	0.805043	0.760364	0.060500	0.027*	
Au1	0.500000	0.500000	0.000000	0.01216 (5)	
Br2	0.75282 (5)	0.32913 (4)	−0.02174 (2)	0.02058 (8)	
Br3	0.39035 (5)	0.28624 (4)	0.13212 (2)	0.01930 (8)	
Au2	1.000000	0.000000	0.500000	0.01012 (5)	
Br4	1.11176 (4)	0.13772 (4)	0.34673 (2)	0.01553 (7)	
Br5	1.08990 (5)	−0.27636 (4)	0.47199 (2)	0.01712 (8)	

### (Bromoselanyl)tris(propan-2-yl)phosphonium tetrabromidoaurate(III) (21b) Atomic displacement parameters (Å^2^)

**Table:**

	*U*^11^	*U*^22^	*U*^33^	*U*^12^	*U*^13^	*U*^23^
P1	0.0091 (4)	0.0110 (3)	0.0099 (4)	0.0006 (3)	−0.0010 (3)	−0.0017 (3)
Se1	0.01397 (17)	0.01460 (15)	0.01786 (18)	0.00421 (12)	−0.00391 (14)	−0.00242 (13)
Br1	0.01892 (18)	0.01379 (15)	0.02116 (18)	0.00242 (12)	−0.00027 (15)	−0.00571 (13)
C1	0.0148 (17)	0.0170 (15)	0.0135 (17)	−0.0009 (13)	−0.0024 (14)	−0.0044 (13)
C2	0.0105 (16)	0.0122 (14)	0.0152 (17)	−0.0020 (12)	−0.0025 (13)	−0.0036 (12)
C3	0.0156 (17)	0.0112 (14)	0.0099 (15)	−0.0001 (12)	0.0002 (13)	−0.0021 (12)
C11	0.025 (2)	0.0156 (16)	0.023 (2)	0.0032 (14)	−0.0040 (16)	−0.0100 (14)
C12	0.0208 (19)	0.0255 (18)	0.0142 (18)	−0.0045 (15)	−0.0009 (15)	−0.0063 (14)
C21	0.0156 (18)	0.0158 (15)	0.0231 (19)	−0.0059 (13)	0.0021 (15)	−0.0027 (14)
C22	0.0124 (17)	0.0197 (16)	0.0226 (19)	0.0023 (13)	−0.0001 (15)	−0.0020 (14)
C31	0.0145 (17)	0.0179 (16)	0.0148 (17)	−0.0008 (13)	0.0017 (14)	−0.0013 (13)
C32	0.026 (2)	0.0162 (15)	0.0108 (16)	−0.0042 (14)	−0.0005 (15)	−0.0015 (13)
Au1	0.01495 (10)	0.01110 (8)	0.01053 (9)	0.00066 (6)	−0.00267 (7)	−0.00217 (6)
Br2	0.02075 (19)	0.02002 (16)	0.01994 (18)	0.00683 (13)	−0.00135 (15)	−0.00417 (14)
Br3	0.02334 (19)	0.01676 (15)	0.01494 (17)	−0.00107 (13)	−0.00055 (15)	0.00151 (13)
Au2	0.00935 (9)	0.01033 (8)	0.01101 (9)	0.00037 (6)	−0.00181 (7)	−0.00286 (6)
Br4	0.01799 (18)	0.01418 (14)	0.01292 (16)	0.00031 (12)	0.00141 (14)	−0.00183 (12)
Br5	0.02189 (18)	0.01260 (14)	0.01684 (17)	0.00474 (12)	−0.00159 (14)	−0.00453 (12)

### (Bromoselanyl)tris(propan-2-yl)phosphonium tetrabromidoaurate(III) (21b) Geometric parameters (Å, º)

**Table:**

P1—C2	1.823 (3)	C21—H21A	0.9800
P1—C3	1.829 (3)	C21—H21B	0.9800
P1—C1	1.832 (3)	C21—H21C	0.9800
P1—Se1	2.2364 (9)	C22—H22A	0.9800
Se1—Br1	2.3179 (5)	C22—H22B	0.9800
Br1—Br2	3.3445 (6)	C22—H22C	0.9800
C1—C11	1.530 (4)	C31—H31A	0.9800
C1—C12	1.545 (5)	C31—H31B	0.9800
C1—H1	1.0000	C31—H31C	0.9800
C2—C21	1.527 (5)	C32—H32A	0.9800
C2—C22	1.533 (4)	C32—H32B	0.9800
C2—H2	1.0000	C32—H32C	0.9800
C3—C32	1.526 (5)	Au1—Br2^i^	2.4162 (4)
C3—C31	1.529 (5)	Au1—Br2	2.4162 (4)
C3—H3	1.0000	Au1—Br3	2.4264 (4)
C11—H11A	0.9800	Au1—Br3^i^	2.4264 (4)
C11—H11B	0.9800	Au2—Br4	2.4230 (4)
C11—H11C	0.9800	Au2—Br4^ii^	2.4230 (4)
C12—H12A	0.9800	Au2—Br5	2.4258 (3)
C12—H12B	0.9800	Au2—Br5^ii^	2.4258 (3)
C12—H12C	0.9800		
			
C2—P1—C3	109.07 (16)	C2—C21—H21A	109.5
C2—P1—C1	116.58 (16)	C2—C21—H21B	109.5
C3—P1—C1	108.74 (15)	H21A—C21—H21B	109.5
C2—P1—Se1	109.29 (10)	C2—C21—H21C	109.5
C3—P1—Se1	112.43 (11)	H21A—C21—H21C	109.5
C1—P1—Se1	100.60 (11)	H21B—C21—H21C	109.5
P1—Se1—Br1	96.32 (3)	C2—C22—H22A	109.5
Se1—Br1—Br2	166.590 (17)	C2—C22—H22B	109.5
C11—C1—C12	111.0 (3)	H22A—C22—H22B	109.5
C11—C1—P1	113.7 (2)	C2—C22—H22C	109.5
C12—C1—P1	112.8 (2)	H22A—C22—H22C	109.5
C11—C1—H1	106.2	H22B—C22—H22C	109.5
C12—C1—H1	106.2	C3—C31—H31A	109.5
P1—C1—H1	106.2	C3—C31—H31B	109.5
C21—C2—C22	112.1 (3)	H31A—C31—H31B	109.5
C21—C2—P1	114.8 (2)	C3—C31—H31C	109.5
C22—C2—P1	110.6 (2)	H31A—C31—H31C	109.5
C21—C2—H2	106.2	H31B—C31—H31C	109.5
C22—C2—H2	106.2	C3—C32—H32A	109.5
P1—C2—H2	106.2	C3—C32—H32B	109.5
C32—C3—C31	111.8 (3)	H32A—C32—H32B	109.5
C32—C3—P1	113.5 (2)	C3—C32—H32C	109.5
C31—C3—P1	110.8 (2)	H32A—C32—H32C	109.5
C32—C3—H3	106.8	H32B—C32—H32C	109.5
C31—C3—H3	106.8	Br2^i^—Au1—Br2	180.0
P1—C3—H3	106.8	Br2^i^—Au1—Br3	89.238 (13)
C1—C11—H11A	109.5	Br2—Au1—Br3	90.763 (13)
C1—C11—H11B	109.5	Br2^i^—Au1—Br3^i^	90.762 (13)
H11A—C11—H11B	109.5	Br2—Au1—Br3^i^	89.237 (13)
C1—C11—H11C	109.5	Br3—Au1—Br3^i^	180.0
H11A—C11—H11C	109.5	Au1—Br2—Br1	85.485 (12)
H11B—C11—H11C	109.5	Br4—Au2—Br4^ii^	180.000 (16)
C1—C12—H12A	109.5	Br4—Au2—Br5	90.629 (12)
C1—C12—H12B	109.5	Br4^ii^—Au2—Br5	89.371 (12)
H12A—C12—H12B	109.5	Br4—Au2—Br5^ii^	89.372 (12)
C1—C12—H12C	109.5	Br4^ii^—Au2—Br5^ii^	90.628 (12)
H12A—C12—H12C	109.5	Br5—Au2—Br5^ii^	179.999 (17)
H12B—C12—H12C	109.5		
			
C2—P1—Se1—Br1	51.37 (12)	Se1—P1—C2—C21	48.5 (3)
C3—P1—Se1—Br1	−69.89 (12)	C3—P1—C2—C22	−60.0 (3)
C1—P1—Se1—Br1	174.59 (11)	C1—P1—C2—C22	63.6 (3)
C2—P1—C1—C11	−78.8 (3)	Se1—P1—C2—C22	176.7 (2)
C3—P1—C1—C11	44.9 (3)	C2—P1—C3—C32	−41.5 (3)
Se1—P1—C1—C11	163.2 (2)	C1—P1—C3—C32	−169.6 (2)
C2—P1—C1—C12	48.8 (3)	Se1—P1—C3—C32	79.9 (3)
C3—P1—C1—C12	172.6 (2)	C2—P1—C3—C31	−168.3 (2)
Se1—P1—C1—C12	−69.2 (3)	C1—P1—C3—C31	63.6 (3)
C3—P1—C2—C21	171.8 (2)	Se1—P1—C3—C31	−46.9 (2)
C1—P1—C2—C21	−64.6 (3)		

Symmetry codes: (i) −*x*+1, −*y*+1, −*z*; (ii) −*x*+2, −*y*, −*z*+1.

### (Bromoselanyl)tris(propan-2-yl)phosphonium tetrabromidoaurate(III) (21b) Hydrogen-bond geometry (Å, º)

**Table:**

*D*—H···*A*	*D*—H	H···*A*	*D*···*A*	*D*—H···*A*
C21—H21*B*···Br1	0.98	3.34	3.927 (4)	120
C3—H3···Br2^iii^	1.00	3.27	3.788 (3)	114
C31—H31*C*···Br2^iii^	0.98	3.08	3.836 (3)	135
C32—H32*A*···Br2^iii^	0.98	2.96	3.740 (3)	137
C1—H1···Br4^iii^	1.00	3.18	4.045 (3)	145
C21—H21*C*···Br4^iv^	0.98	3.08	3.953 (3)	149
C31—H31*B*···Br4^iii^	0.98	3.03	3.972 (4)	162
C1—H1···Br5^iii^	1.00	2.93	3.756 (4)	140
C22—H22*C*···Br5^v^	0.98	3.15	3.893 (4)	134

Symmetry codes: (iii) *x*, *y*+1, *z*; (iv) *x*−1, *y*, *z*; (v) *x*−1, *y*+1, *z*.

### (Bromoselanyl)(tert-butyl)bis(propan-2-yl)phosphonium tetrabromidoaurate(III) (22b) Crystal data

**Table:**

(C_10_H_23_BrPSe)[AuBr_4_]	*Z* = 2
*M**_r_* = 849.73	*F*(000) = 772
Triclinic, *P*1	*D*_x_ = 2.822 Mg m^−^^3^
*a* = 7.8155 (3) Å	Mo *K*α radiation, λ = 0.71073 Å
*b* = 9.1505 (3) Å	Cell parameters from 21572 reflections
*c* = 15.5221 (5) Å	θ = 2.4–30.8°
α = 85.965 (2)°	µ = 19.23 mm^−^^1^
β = 80.294 (3)°	*T* = 100 K
γ = 66.049 (3)°	Plate, red
*V* = 999.97 (6) Å^3^	0.3 × 0.2 × 0.03 mm

### (Bromoselanyl)(tert-butyl)bis(propan-2-yl)phosphonium tetrabromidoaurate(III) (22b) Data collection

**Table:**

Oxford Diffraction Xcalibur, Eos diffractometer	5847 independent reflections
Radiation source: Enhance (Mo) X-ray Source	5198 reflections with *I* > 2σ(*I*)
Detector resolution: 16.1419 pixels mm^-1^	*R*_int_ = 0.050
ω scans	θ_max_ = 30.9°, θ_min_ = 2.4°
Absorption correction: multi-scan (CrysAlisPro; Rigaku OD, 2015)	*h* = −11→11
*T*_min_ = 0.202, *T*_max_ = 1.000	*k* = −13→13
52431 measured reflections	*l* = −22→22

### (Bromoselanyl)(tert-butyl)bis(propan-2-yl)phosphonium tetrabromidoaurate(III) (22b) Refinement

**Table:**

Refinement on *F*^2^	Primary atom site location: structure-invariant direct methods
Least-squares matrix: full	Secondary atom site location: difference Fourier map
*R*[*F*^2^ > 2σ(*F*^2^)] = 0.025	Hydrogen site location: inferred from neighbouring sites
*wR*(*F*^2^) = 0.063	H-atom parameters constrained
*S* = 1.04	*w* = 1/[σ^2^(*F*_o_^2^) + (0.0295*P*)^2^ + 3.0506*P*] where *P* = (*F*_o_^2^ + 2*F*_c_^2^)/3
5847 reflections	(Δ/σ)_max_ = 0.001
173 parameters	Δρ_max_ = 1.71 e Å^−^^3^
0 restraints	Δρ_min_ = −1.44 e Å^−^^3^

### (Bromoselanyl)(tert-butyl)bis(propan-2-yl)phosphonium tetrabromidoaurate(III) (22b) Fractional atomic coordinates and isotropic or equivalent isotropic displacement parameters (Å^2^)

**Table:**

	*x*	*y*	*z*	*U*_iso_*/*U*_eq_	
P1	0.50396 (13)	0.10486 (11)	0.24702 (6)	0.01234 (17)	
Se1	0.37707 (5)	−0.06510 (4)	0.30872 (3)	0.01717 (8)	
Br1	0.14096 (5)	0.10400 (5)	0.41193 (3)	0.01896 (8)	
C1	0.7420 (5)	−0.0300 (4)	0.1909 (2)	0.0154 (7)	
C2	0.5064 (6)	0.2349 (5)	0.3307 (3)	0.0202 (8)	
H2	0.370536	0.303427	0.351902	0.024*	
C3	0.3564 (6)	0.2349 (4)	0.1684 (3)	0.0173 (7)	
H3	0.430348	0.292376	0.134040	0.021*	
C11	0.8062 (6)	0.0571 (5)	0.1123 (3)	0.0229 (9)	
H11A	0.803491	0.157676	0.131775	0.034*	
H11B	0.720630	0.079831	0.069005	0.034*	
H11C	0.935429	−0.010648	0.085845	0.034*	
C12	0.8846 (6)	−0.0773 (5)	0.2559 (3)	0.0212 (8)	
H12A	1.006172	−0.157871	0.229224	0.032*	
H12B	0.835837	−0.121394	0.309079	0.032*	
H12C	0.902346	0.017404	0.270870	0.032*	
C13	0.7354 (6)	−0.1838 (5)	0.1604 (3)	0.0218 (8)	
H13A	0.634662	−0.155692	0.124430	0.033*	
H13B	0.709770	−0.245346	0.211417	0.033*	
H13C	0.857404	−0.248159	0.125820	0.033*	
C21	0.5911 (6)	0.1508 (6)	0.4122 (3)	0.0271 (10)	
H21A	0.729668	0.104162	0.398448	0.041*	
H21B	0.546541	0.065956	0.430672	0.041*	
H21C	0.551020	0.228693	0.459541	0.041*	
C22	0.5960 (7)	0.3499 (6)	0.2896 (3)	0.0273 (9)	
H22A	0.584526	0.425951	0.333887	0.041*	
H22B	0.530519	0.408286	0.241327	0.041*	
H22C	0.730240	0.288839	0.267432	0.041*	
C31	0.3155 (7)	0.1397 (5)	0.1030 (3)	0.0256 (9)	
H31A	0.241760	0.082244	0.134354	0.038*	
H31B	0.435336	0.062881	0.072387	0.038*	
H31C	0.243202	0.213235	0.060597	0.038*	
C32	0.1709 (6)	0.3628 (5)	0.2133 (3)	0.0226 (9)	
H32A	0.100581	0.431734	0.168905	0.034*	
H32B	0.199553	0.427409	0.251845	0.034*	
H32C	0.094104	0.310846	0.247835	0.034*	
Au1	0.000000	0.500000	0.500000	0.01431 (5)	
Br2	−0.17342 (6)	0.34529 (5)	0.57133 (3)	0.02321 (9)	
Br3	0.22409 (6)	0.39711 (5)	0.60184 (3)	0.02503 (9)	
Au2	0.500000	0.500000	0.000000	0.01293 (5)	
Br4	0.34937 (6)	0.69967 (5)	0.11370 (3)	0.02330 (9)	
Br5	0.80787 (5)	0.45789 (5)	0.03378 (3)	0.01988 (8)	

### (Bromoselanyl)(tert-butyl)bis(propan-2-yl)phosphonium tetrabromidoaurate(III) (22b) Atomic displacement parameters (Å^2^)

**Table:**

	*U*^11^	*U*^22^	*U*^33^	*U*^12^	*U*^13^	*U*^23^
P1	0.0121 (4)	0.0126 (4)	0.0121 (4)	−0.0049 (3)	−0.0017 (3)	0.0003 (3)
Se1	0.01440 (17)	0.01364 (17)	0.02165 (19)	−0.00536 (14)	0.00047 (14)	0.00209 (14)
Br1	0.01548 (17)	0.02158 (18)	0.01890 (18)	−0.00821 (14)	0.00178 (14)	0.00010 (14)
C1	0.0126 (16)	0.0155 (17)	0.0146 (17)	−0.0026 (14)	−0.0007 (13)	0.0004 (13)
C2	0.0192 (19)	0.0231 (19)	0.0190 (19)	−0.0096 (16)	−0.0008 (15)	−0.0032 (15)
C3	0.0183 (18)	0.0143 (17)	0.0166 (18)	−0.0031 (14)	−0.0052 (14)	0.0024 (14)
C11	0.021 (2)	0.023 (2)	0.020 (2)	−0.0072 (16)	0.0031 (16)	0.0031 (16)
C12	0.0127 (17)	0.025 (2)	0.024 (2)	−0.0048 (15)	−0.0039 (15)	0.0001 (16)
C13	0.0193 (19)	0.0181 (18)	0.022 (2)	−0.0024 (15)	0.0013 (16)	−0.0047 (15)
C21	0.024 (2)	0.047 (3)	0.0137 (19)	−0.016 (2)	−0.0047 (16)	−0.0043 (18)
C22	0.027 (2)	0.029 (2)	0.031 (2)	−0.0158 (19)	−0.0029 (19)	−0.0076 (19)
C31	0.032 (2)	0.0193 (19)	0.022 (2)	−0.0027 (17)	−0.0143 (18)	−0.0022 (16)
C32	0.0182 (19)	0.0193 (19)	0.024 (2)	−0.0013 (15)	−0.0039 (16)	0.0030 (16)
Au1	0.01577 (10)	0.01210 (9)	0.01502 (10)	−0.00555 (7)	−0.00218 (7)	−0.00049 (7)
Br2	0.0242 (2)	0.02110 (19)	0.0269 (2)	−0.01315 (16)	−0.00151 (16)	0.00323 (16)
Br3	0.0259 (2)	0.0288 (2)	0.0240 (2)	−0.01270 (17)	−0.01168 (17)	0.00647 (16)
Au2	0.00891 (9)	0.01081 (9)	0.01807 (10)	−0.00374 (7)	−0.00046 (7)	0.00113 (7)
Br4	0.01693 (18)	0.02185 (19)	0.0283 (2)	−0.00550 (15)	0.00196 (16)	−0.00920 (16)
Br5	0.01169 (16)	0.01865 (18)	0.0301 (2)	−0.00635 (14)	−0.00504 (15)	0.00129 (15)

### (Bromoselanyl)(tert-butyl)bis(propan-2-yl)phosphonium tetrabromidoaurate(III) (22b) Geometric parameters (Å, º)

**Table:**

P1—C2	1.829 (4)	C13—H13B	0.9800
P1—C3	1.841 (4)	C13—H13C	0.9800
P1—C1	1.866 (4)	C21—H21A	0.9800
P1—Se1	2.2453 (10)	C21—H21B	0.9800
Se1—Br1	2.3237 (5)	C21—H21C	0.9800
Br1—Br2	3.3687 (6)	C22—H22A	0.9800
C1—C11	1.536 (6)	C22—H22B	0.9800
C1—C13	1.539 (6)	C22—H22C	0.9800
C1—C12	1.540 (6)	C31—H31A	0.9800
C2—C22	1.536 (6)	C31—H31B	0.9800
C2—C21	1.538 (6)	C31—H31C	0.9800
C2—H2	1.0000	C32—H32A	0.9800
C3—C31	1.533 (6)	C32—H32B	0.9800
C3—C32	1.534 (5)	C32—H32C	0.9800
C3—H3	1.0000	Au1—Br3	2.4162 (4)
C11—H11A	0.9800	Au1—Br3^i^	2.4162 (4)
C11—H11B	0.9800	Au1—Br2	2.4287 (4)
C11—H11C	0.9800	Au1—Br2^i^	2.4287 (4)
C12—H12A	0.9800	Au2—Br4	2.4178 (4)
C12—H12B	0.9800	Au2—Br4^ii^	2.4179 (4)
C12—H12C	0.9800	Au2—Br5^ii^	2.4202 (4)
C13—H13A	0.9800	Au2—Br5	2.4202 (4)
			
C2—P1—C3	107.07 (19)	C1—C13—H13C	109.5
C2—P1—C1	115.23 (19)	H13A—C13—H13C	109.5
C3—P1—C1	111.45 (17)	H13B—C13—H13C	109.5
C2—P1—Se1	109.82 (14)	C2—C21—H21A	109.5
C3—P1—Se1	109.70 (14)	C2—C21—H21B	109.5
C1—P1—Se1	103.51 (13)	H21A—C21—H21B	109.5
P1—Se1—Br1	99.52 (3)	C2—C21—H21C	109.5
Se1—Br1—Br2	175.31 (2)	H21A—C21—H21C	109.5
C11—C1—C13	109.9 (3)	H21B—C21—H21C	109.5
C11—C1—C12	109.6 (3)	C2—C22—H22A	109.5
C13—C1—C12	108.2 (3)	C2—C22—H22B	109.5
C11—C1—P1	109.6 (3)	H22A—C22—H22B	109.5
C13—C1—P1	110.4 (3)	C2—C22—H22C	109.5
C12—C1—P1	109.0 (3)	H22A—C22—H22C	109.5
C22—C2—C21	111.9 (4)	H22B—C22—H22C	109.5
C22—C2—P1	110.4 (3)	C3—C31—H31A	109.5
C21—C2—P1	116.2 (3)	C3—C31—H31B	109.5
C22—C2—H2	105.8	H31A—C31—H31B	109.5
C21—C2—H2	105.8	C3—C31—H31C	109.5
P1—C2—H2	105.8	H31A—C31—H31C	109.5
C31—C3—C32	110.4 (4)	H31B—C31—H31C	109.5
C31—C3—P1	112.4 (3)	C3—C32—H32A	109.5
C32—C3—P1	112.6 (3)	C3—C32—H32B	109.5
C31—C3—H3	107.0	H32A—C32—H32B	109.5
C32—C3—H3	107.0	C3—C32—H32C	109.5
P1—C3—H3	107.0	H32A—C32—H32C	109.5
C1—C11—H11A	109.5	H32B—C32—H32C	109.5
C1—C11—H11B	109.5	Br3—Au1—Br3^i^	180.0
H11A—C11—H11B	109.5	Br3—Au1—Br2	90.432 (15)
C1—C11—H11C	109.5	Br3^i^—Au1—Br2	89.568 (15)
H11A—C11—H11C	109.5	Br3—Au1—Br2^i^	89.568 (15)
H11B—C11—H11C	109.5	Br3^i^—Au1—Br2^i^	90.432 (15)
C1—C12—H12A	109.5	Br2—Au1—Br2^i^	180.000 (13)
C1—C12—H12B	109.5	Au1—Br2—Br1	75.542 (13)
H12A—C12—H12B	109.5	Br4—Au2—Br4^ii^	179.999 (15)
C1—C12—H12C	109.5	Br4—Au2—Br5^ii^	89.894 (14)
H12A—C12—H12C	109.5	Br4^ii^—Au2—Br5^ii^	90.105 (14)
H12B—C12—H12C	109.5	Br4—Au2—Br5	90.106 (14)
C1—C13—H13A	109.5	Br4^ii^—Au2—Br5	89.895 (14)
C1—C13—H13B	109.5	Br5^ii^—Au2—Br5	180.0
H13A—C13—H13B	109.5		
			
C2—P1—Se1—Br1	37.67 (15)	C3—P1—C2—C22	−61.9 (3)
C3—P1—Se1—Br1	−79.74 (13)	C1—P1—C2—C22	62.6 (3)
C1—P1—Se1—Br1	161.21 (13)	Se1—P1—C2—C22	179.0 (3)
C2—P1—C1—C11	−87.6 (3)	C3—P1—C2—C21	169.3 (3)
C3—P1—C1—C11	34.6 (3)	C1—P1—C2—C21	−66.1 (4)
Se1—P1—C1—C11	152.5 (3)	Se1—P1—C2—C21	50.2 (3)
C2—P1—C1—C13	151.1 (3)	C2—P1—C3—C31	−170.6 (3)
C3—P1—C1—C13	−86.7 (3)	C1—P1—C3—C31	62.5 (4)
Se1—P1—C1—C13	31.2 (3)	Se1—P1—C3—C31	−51.5 (3)
C2—P1—C1—C12	32.4 (3)	C2—P1—C3—C32	−45.2 (4)
C3—P1—C1—C12	154.7 (3)	C1—P1—C3—C32	−172.0 (3)
Se1—P1—C1—C12	−87.5 (3)	Se1—P1—C3—C32	74.0 (3)

Symmetry codes: (i) −*x*, −*y*+1, −*z*+1; (ii) −*x*+1, −*y*+1, −*z*.

### (Bromoselanyl)(tert-butyl)bis(propan-2-yl)phosphonium tetrabromidoaurate(III) (22b) Hydrogen-bond geometry (Å, º)

**Table:**

*D*—H···*A*	*D*—H	H···*A*	*D*···*A*	*D*—H···*A*
C13—H13*B*···Se1	0.98	2.70	3.167 (4)	109
C2—H2···Br1	1.00	3.05	3.540 (4)	112
C3—H3···Au2	1.00	2.85	3.783 (4)	155
C32—H32*C*···Br1	0.98	3.05	3.788 (4)	134
C21—H21*C*···Br3	0.98	3.03	3.848 (4)	142
C11—H11*A*···Br5	0.98	3.05	3.787 (4)	133
C2—H2···Br2^i^	1.00	3.17	3.901 (4)	131
C22—H22*A*···Br3^iii^	0.98	2.92	3.764 (4)	144
C12—H12*B*···Br3^iv^	0.98	2.96	3.826 (4)	148
C13—H13*B*···Br3^iv^	0.98	3.15	4.051 (4)	153
C12—H12*A*···Br4^v^	0.98	2.82	3.761 (4)	160
C13—H13*A*···Br4^vi^	0.98	3.05	3.771 (5)	132

Symmetry codes: (i) −*x*, −*y*+1, −*z*+1; (iii) −*x*+1, −*y*+1, −*z*+1; (iv) −*x*+1, −*y*, −*z*+1; (v) *x*+1, *y*−1, *z*; (vi) *x*, *y*−1, *z*.

### (Bromoselanyl)bis(tert-butyl)(propan-2-yl)phosphonium tetrabromidoaurate(III) (23b) Crystal data

**Table:**

(C_11_H_25_BrPSe)[AuBr_4_]	*F*(000) = 1576
*M**_r_* = 863.76	*D*_x_ = 2.711 Mg m^−^^3^
Monoclinic, *P*2_1_/*n*	Mo *K*α radiation, λ = 0.71073 Å
*a* = 12.3529 (4) Å	Cell parameters from 13739 reflections
*b* = 10.4233 (4) Å	θ = 2.1–30.8°
*c* = 16.4635 (5) Å	µ = 18.18 mm^−^^1^
β = 93.453 (3)°	*T* = 101 K
*V* = 2115.97 (12) Å^3^	Plate, dichroic red/orange
*Z* = 4	0.2 × 0.1 × 0.02 mm

### (Bromoselanyl)bis(tert-butyl)(propan-2-yl)phosphonium tetrabromidoaurate(III) (23b) Data collection

**Table:**

Oxford Diffraction Xcalibur, Eos diffractometer	7063 independent reflections
Radiation source: Enhance (Mo) X-ray Source	4089 reflections with *I* > 2σ(*I*)
Detector resolution: 16.1419 pixels mm^-1^	*R*_int_ = 0.0000
ω scan	θ_max_ = 28.3°, θ_min_ = 2.3°
Absorption correction: multi-scan (CrysAlisPro; Rigaku OD, 2015)	*h* = −16→16
*T*_min_ = 0.122, *T*_max_ = 0.713	*k* = −13→13
7063 measured reflections	*l* = −21→21

### (Bromoselanyl)bis(tert-butyl)(propan-2-yl)phosphonium tetrabromidoaurate(III) (23b) Refinement

**Table:**

Refinement on *F*^2^	Primary atom site location: structure-invariant direct methods
Least-squares matrix: full	Secondary atom site location: difference Fourier map
*R*[*F*^2^ > 2σ(*F*^2^)] = 0.034	Hydrogen site location: inferred from neighbouring sites
*wR*(*F*^2^) = 0.061	H-atom parameters constrained
*S* = 0.81	*w* = 1/[σ^2^(*F*_o_^2^) + (0.0239*P*)^2^] where *P* = (*F*_o_^2^ + 2*F*_c_^2^)/3
7063 reflections	(Δ/σ)_max_ < 0.001
184 parameters	Δρ_max_ = 1.72 e Å^−^^3^
66 restraints	Δρ_min_ = −1.10 e Å^−^^3^

### (Bromoselanyl)bis(tert-butyl)(propan-2-yl)phosphonium tetrabromidoaurate(III) (23b) Special details

**Table:**

Geometry. All esds (except the esd in the dihedral angle between two l.s. planes) are estimated using the full covariance matrix. The cell esds are taken into account individually in the estimation of esds in distances, angles and torsion angles; correlations between esds in cell parameters are only used when they are defined by crystal symmetry. An approximate (isotropic) treatment of cell esds is used for estimating esds involving l.s. planes.
Refinement. The crystal was a two-component non-merohedral twin (by 180 degree rotation about the a* axis). The data reduction generated all non- overlapped reflections from the larger component, together with all overlapped reflections. The number of data used for refinement should therefore be interpreted with caution. All equivalents were merged during the untwinning process, and the R(int) value is thus meaningless. The "HKLF 5" method was used for structure refinement. The relative volume of the smaller twin component refined to 0.0807 (6). The U values of the carbon atoms were restrained to be less anisotropic using the command "ISOR $C 0.005".

### (Bromoselanyl)bis(tert-butyl)(propan-2-yl)phosphonium tetrabromidoaurate(III) (23b) Fractional atomic coordinates and isotropic or equivalent isotropic displacement parameters (Å^2^)

**Table:**

	*x*	*y*	*z*	*U*_iso_*/*U*_eq_	
P1	0.47957 (15)	0.09088 (17)	0.25502 (11)	0.0126 (5)	
Se1	0.44215 (7)	0.27120 (7)	0.18322 (4)	0.01936 (19)	
Br1	0.51433 (7)	0.42888 (7)	0.27054 (5)	0.0248 (2)	
C2	0.3787 (6)	0.0765 (7)	0.3332 (4)	0.0218 (19)	
C1	0.4675 (6)	−0.0298 (6)	0.1701 (4)	0.0176 (18)	
C3	0.6156 (6)	0.1003 (6)	0.3049 (4)	0.0153 (17)	
H3	0.607781	0.152437	0.355216	0.018*	
C21	0.4077 (8)	−0.0295 (8)	0.3953 (4)	0.039 (3)	
H21A	0.351865	−0.034008	0.434986	0.059*	
H21B	0.478024	−0.010441	0.423418	0.059*	
H21C	0.411786	−0.112003	0.367010	0.059*	
C22	0.2654 (6)	0.0530 (7)	0.2937 (5)	0.030 (2)	
H22A	0.262312	−0.032758	0.269269	0.045*	
H22B	0.249411	0.117542	0.251352	0.045*	
H22C	0.211805	0.059172	0.335033	0.045*	
C23	0.3763 (7)	0.2033 (7)	0.3804 (5)	0.036 (2)	
H23A	0.331979	0.192879	0.427376	0.054*	
H23B	0.344971	0.270664	0.344723	0.054*	
H23C	0.450286	0.227282	0.399210	0.054*	
C11	0.5717 (7)	−0.0224 (7)	0.1231 (4)	0.029 (2)	
H11A	0.562872	−0.074840	0.073791	0.044*	
H11B	0.633200	−0.054452	0.157640	0.044*	
H11C	0.585108	0.066971	0.108119	0.044*	
C12	0.4547 (7)	−0.1648 (6)	0.2060 (4)	0.025 (2)	
H12A	0.382333	−0.173352	0.226697	0.038*	
H12B	0.509771	−0.177957	0.250631	0.038*	
H12C	0.463964	−0.229158	0.163584	0.038*	
C13	0.3680 (7)	0.0002 (7)	0.1136 (4)	0.0282 (19)	
H13A	0.377566	0.083827	0.087731	0.042*	
H13B	0.303290	0.002393	0.145169	0.042*	
H13C	0.359449	−0.066272	0.071617	0.042*	
C31	0.6995 (6)	0.1702 (7)	0.2571 (4)	0.0236 (19)	
H31A	0.760721	0.195971	0.294156	0.035*	
H31B	0.666388	0.246598	0.231329	0.035*	
H31C	0.725290	0.113057	0.215166	0.035*	
C32	0.6621 (6)	−0.0294 (7)	0.3344 (4)	0.028 (2)	
H32A	0.679990	−0.081171	0.287364	0.041*	
H32B	0.608123	−0.074674	0.364954	0.041*	
H32C	0.727718	−0.015047	0.369636	0.041*	
Au1	0.500000	0.500000	0.500000	0.01339 (10)	
Br2	0.60826 (7)	0.63241 (8)	0.41494 (5)	0.0277 (2)	
Br3	0.64014 (7)	0.33828 (8)	0.50404 (5)	0.0293 (2)	
Au2	0.500000	0.500000	0.000000	0.01182 (10)	
Br4	0.63241 (6)	0.32858 (7)	0.01148 (5)	0.01770 (19)	
Br5	0.35622 (6)	0.34365 (7)	−0.02110 (4)	0.01917 (19)	

### (Bromoselanyl)bis(tert-butyl)(propan-2-yl)phosphonium tetrabromidoaurate(III) (23b) Atomic displacement parameters (Å^2^)

**Table:**

	*U*^11^	*U*^22^	*U*^33^	*U*^12^	*U*^13^	*U*^23^
P1	0.0157 (13)	0.0116 (10)	0.0107 (10)	0.0009 (8)	0.0023 (9)	−0.0006 (8)
Se1	0.0256 (5)	0.0129 (4)	0.0191 (4)	0.0043 (4)	−0.0032 (3)	0.0009 (3)
Br1	0.0270 (5)	0.0136 (4)	0.0336 (5)	−0.0007 (4)	−0.0005 (4)	−0.0034 (3)
C2	0.020 (4)	0.026 (4)	0.019 (4)	−0.003 (3)	0.004 (3)	−0.004 (3)
C1	0.028 (4)	0.009 (4)	0.015 (3)	0.002 (3)	−0.002 (3)	−0.004 (3)
C3	0.016 (4)	0.013 (3)	0.017 (3)	−0.002 (3)	−0.001 (3)	0.000 (3)
C21	0.055 (6)	0.043 (6)	0.021 (4)	−0.010 (4)	0.011 (4)	0.010 (4)
C22	0.028 (5)	0.029 (4)	0.035 (5)	−0.009 (4)	0.014 (4)	−0.013 (4)
C23	0.038 (5)	0.034 (5)	0.039 (5)	−0.005 (4)	0.026 (4)	−0.016 (4)
C11	0.046 (5)	0.018 (5)	0.025 (4)	0.000 (4)	0.012 (4)	−0.008 (3)
C12	0.035 (5)	0.022 (4)	0.018 (4)	−0.005 (4)	−0.006 (4)	−0.003 (3)
C13	0.043 (5)	0.024 (4)	0.016 (4)	0.002 (4)	−0.014 (3)	0.001 (4)
C31	0.017 (4)	0.021 (4)	0.033 (4)	0.003 (3)	0.004 (4)	−0.002 (3)
C32	0.022 (4)	0.023 (5)	0.037 (5)	0.004 (3)	−0.010 (4)	0.011 (3)
Au1	0.0126 (2)	0.0117 (2)	0.0160 (2)	0.0003 (2)	0.00209 (17)	−0.0027 (2)
Br2	0.0375 (6)	0.0214 (4)	0.0256 (4)	−0.0115 (4)	0.0142 (4)	−0.0048 (4)
Br3	0.0203 (5)	0.0212 (5)	0.0456 (6)	0.0078 (4)	−0.0036 (4)	−0.0058 (4)
Au2	0.0129 (2)	0.0111 (2)	0.0116 (2)	−0.0027 (2)	0.00226 (16)	−0.00071 (19)
Br4	0.0162 (5)	0.0147 (4)	0.0224 (4)	0.0009 (3)	0.0028 (4)	−0.0025 (3)
Br5	0.0163 (4)	0.0153 (4)	0.0256 (5)	−0.0061 (4)	−0.0020 (3)	0.0010 (3)

### (Bromoselanyl)bis(tert-butyl)(propan-2-yl)phosphonium tetrabromidoaurate(III) (23b) Geometric parameters (Å, º)

**Table:**

P1—C3	1.828 (7)	C11—H11A	0.9800
P1—C2	1.851 (7)	C11—H11B	0.9800
P1—C1	1.880 (7)	C11—H11C	0.9800
P1—Se1	2.2534 (19)	C12—H12A	0.9800
Se1—Br1	2.3255 (10)	C12—H12B	0.9800
Br1—Br2	3.3416 (11)	C12—H12C	0.9800
C2—C22	1.528 (10)	C13—H13A	0.9800
C2—C21	1.533 (10)	C13—H13B	0.9800
C2—C23	1.533 (9)	C13—H13C	0.9800
C1—C13	1.528 (10)	C31—H31A	0.9800
C1—C12	1.538 (9)	C31—H31B	0.9800
C1—C11	1.543 (10)	C31—H31C	0.9800
C3—C31	1.524 (9)	C32—H32A	0.9800
C3—C32	1.536 (9)	C32—H32B	0.9800
C3—H3	1.0000	C32—H32C	0.9800
C21—H21A	0.9800	Au1—Br3^i^	2.4142 (8)
C21—H21B	0.9800	Au1—Br3	2.4142 (8)
C21—H21C	0.9800	Au1—Br2^i^	2.4249 (8)
C22—H22A	0.9800	Au1—Br2	2.4249 (8)
C22—H22B	0.9800	Au2—Br5^ii^	2.4201 (7)
C22—H22C	0.9800	Au2—Br5	2.4201 (7)
C23—H23A	0.9800	Au2—Br4	2.4221 (7)
C23—H23B	0.9800	Au2—Br4^ii^	2.4221 (7)
C23—H23C	0.9800		
			
C3—P1—C2	109.3 (3)	C1—C11—H11A	109.5
C3—P1—C1	113.6 (3)	C1—C11—H11B	109.5
C2—P1—C1	115.9 (3)	H11A—C11—H11B	109.5
C3—P1—Se1	110.2 (2)	C1—C11—H11C	109.5
C2—P1—Se1	107.9 (3)	H11A—C11—H11C	109.5
C1—P1—Se1	99.4 (2)	H11B—C11—H11C	109.5
P1—Se1—Br1	101.91 (6)	C1—C12—H12A	109.5
Se1—Br1—Br2	172.85 (4)	C1—C12—H12B	109.5
C22—C2—C21	109.8 (6)	H12A—C12—H12B	109.5
C22—C2—C23	108.0 (6)	C1—C12—H12C	109.5
C21—C2—C23	107.2 (6)	H12A—C12—H12C	109.5
C22—C2—P1	110.7 (5)	H12B—C12—H12C	109.5
C21—C2—P1	112.4 (6)	C1—C13—H13A	109.5
C23—C2—P1	108.5 (5)	C1—C13—H13B	109.5
C13—C1—C12	108.9 (6)	H13A—C13—H13B	109.5
C13—C1—C11	110.4 (6)	C1—C13—H13C	109.5
C12—C1—C11	110.3 (6)	H13A—C13—H13C	109.5
C13—C1—P1	109.9 (5)	H13B—C13—H13C	109.5
C12—C1—P1	109.4 (5)	C3—C31—H31A	109.5
C11—C1—P1	107.9 (5)	C3—C31—H31B	109.5
C31—C3—C32	109.3 (6)	H31A—C31—H31B	109.5
C31—C3—P1	115.4 (5)	C3—C31—H31C	109.5
C32—C3—P1	114.3 (5)	H31A—C31—H31C	109.5
C31—C3—H3	105.6	H31B—C31—H31C	109.5
C32—C3—H3	105.6	C3—C32—H32A	109.5
P1—C3—H3	105.6	C3—C32—H32B	109.5
C2—C21—H21A	109.5	H32A—C32—H32B	109.5
C2—C21—H21B	109.5	C3—C32—H32C	109.5
H21A—C21—H21B	109.5	H32A—C32—H32C	109.5
C2—C21—H21C	109.5	H32B—C32—H32C	109.5
H21A—C21—H21C	109.5	Br3^i^—Au1—Br3	180.00 (3)
H21B—C21—H21C	109.5	Br3^i^—Au1—Br2^i^	89.64 (3)
C2—C22—H22A	109.5	Br3—Au1—Br2^i^	90.36 (3)
C2—C22—H22B	109.5	Br3^i^—Au1—Br2	90.36 (3)
H22A—C22—H22B	109.5	Br3—Au1—Br2	89.64 (3)
C2—C22—H22C	109.5	Br2^i^—Au1—Br2	180.0
H22A—C22—H22C	109.5	Au1—Br2—Br1	82.51 (3)
H22B—C22—H22C	109.5	Br5^ii^—Au2—Br5	180.0
C2—C23—H23A	109.5	Br5^ii^—Au2—Br4	89.96 (2)
C2—C23—H23B	109.5	Br5—Au2—Br4	90.04 (2)
H23A—C23—H23B	109.5	Br5^ii^—Au2—Br4^ii^	90.04 (2)
C2—C23—H23C	109.5	Br5—Au2—Br4^ii^	89.96 (2)
H23A—C23—H23C	109.5	Br4—Au2—Br4^ii^	180.0
H23B—C23—H23C	109.5		
			
C3—P1—Se1—Br1	−40.8 (2)	Se1—P1—C1—C13	−43.0 (5)
C2—P1—Se1—Br1	78.4 (3)	C3—P1—C1—C12	80.4 (6)
C1—P1—Se1—Br1	−160.4 (2)	C2—P1—C1—C12	−47.3 (7)
C3—P1—C2—C22	−173.5 (5)	Se1—P1—C1—C12	−162.6 (5)
C1—P1—C2—C22	−43.7 (6)	C3—P1—C1—C11	−39.6 (6)
Se1—P1—C2—C22	66.7 (5)	C2—P1—C1—C11	−167.3 (5)
C3—P1—C2—C21	−50.2 (6)	Se1—P1—C1—C11	77.4 (5)
C1—P1—C2—C21	79.6 (6)	C2—P1—C3—C31	−153.5 (5)
Se1—P1—C2—C21	−170.0 (5)	C1—P1—C3—C31	75.4 (6)
C3—P1—C2—C23	68.2 (6)	Se1—P1—C3—C31	−35.2 (5)
C1—P1—C2—C23	−162.0 (5)	C2—P1—C3—C32	78.5 (6)
Se1—P1—C2—C23	−51.6 (6)	C1—P1—C3—C32	−52.6 (6)
C3—P1—C1—C13	−160.0 (5)	Se1—P1—C3—C32	−163.2 (4)
C2—P1—C1—C13	72.2 (6)		

Symmetry codes: (i) −*x*+1, −*y*+1, −*z*+1; (ii) −*x*+1, −*y*+1, −*z*.

### (Bromoselanyl)bis(tert-butyl)(propan-2-yl)phosphonium tetrabromidoaurate(III) (23b) Hydrogen-bond geometry (Å, º)

**Table:**

*D*—H···*A*	*D*—H	H···*A*	*D*···*A*	*D*—H···*A*
C13—H13*A*···Se1	0.98	2.60	3.163 (8)	116
C31—H31*B*···Br1	0.98	2.78	3.551 (7)	137
C23—H23*B*···Br1	0.98	2.98	3.476 (8)	112
C3—H3···Br3	1.00	3.13	4.108 (7)	166
C23—H23*C*···Br3	0.98	3.05	3.994 (9)	161
C11—H11*C*···Br4	0.98	3.23	4.182 (7)	165
C31—H31*C*···Br2^iii^	0.98	3.06	3.825 (7)	136
C23—H23*A*···Br4^iv^	0.98	2.91	3.824 (7)	156
C13—H13*C*···Br4^v^	0.98	3.06	3.998 (8)	160
C32—H32*C*···Br4^iii^	0.98	3.01	3.782 (7)	136
C11—H11*A*···Br5^v^	0.98	3.12	3.874 (7)	135
C32—H32*C*···Br5^vi^	0.98	2.93	3.804 (8)	149

Symmetry codes: (iii) −*x*+3/2, *y*−1/2, −*z*+1/2; (iv) *x*−1/2, −*y*+1/2, *z*+1/2; (v) −*x*+1, −*y*, −*z*; (vi) *x*+1/2, −*y*+1/2, *z*+1/2.
